# Dinuclear Cyclometalated Pincer Nickel(II) Complexes with Metal‐Metal‐to‐Ligand Charge Transfer Excited States and Near‐Infrared Emission

**DOI:** 10.1002/anie.202414411

**Published:** 2024-11-07

**Authors:** Mengyue Gao, Wai‐Pong To, Glenna So Ming Tong, Lili Du, Kam‐Hung Low, Zhou Tang, Wei Lu, Chi‐Ming Che

**Affiliations:** ^1^ Department of Chemistry State Key Laboratory of Synthetic Chemistry CAS-HKU Joint Laboratory on New Materials The University of Hong Kong Pokfulam Road Hong Kong P. R. China; ^2^ HKU Shenzhen Institute of Research and Innovation Shenzhen Guangdong 518057 P. R. China; ^3^ Department of Chemistry Southern University of Science and Technology Shenzhen Guangdong 518055 P. R. China; ^4^ Laboratory for Synthetic Chemistry and Chemical Biology Limited Units 1503-1511, 15/F, Building 17W, Hong Kong Science Park, New Territories Hong Kong P. R. China

**Keywords:** Earth abundant metal, Nickel, NIR emission, Phosphorescence

## Abstract

Facile non‐radiative decay of low‐lying metal‐centered (MC) dd excited states has been well documented to pose a significant obstacle to the development of phosphorescent Ni^II^ complexes due to substantial structural distortions between the dd excited state and the ground state. Herein, we prepared a series of dinuclear Ni_2_^II,II^ complexes by using strong σ‐donating carbene‐phenyl‐carbene (C_NHC_ C_phenyl_ C_NHC_) pincer ligands, and prepared their dinuclear Pt_2_^II,II^ and Pd_2_^II,II^ analogues. Dinuclear Ni_2_^II,II^ complexes bridged by formamidinate/α‐carbolinato ligand exhibit short Ni−Ni distances of 2.947–3.054 Å and singlet metal‐metal‐to‐ligand charge transfer (^1^MMLCT) transitions at 500–550 nm. Their ^1^MMLCT absorption energies are red‐shifted relative to the Pt_2_^II,II^ and Pd_2_^II,II^ analogues at ~450 nm and ≤420 nm respectively. One‐electron oxidation of these Ni_2_^II,II^ complexes produces valence‐trapped dinuclear Ni_2_^II,III^ species, which are characterized by EPR spectroscopy. Upon photoexcitation, these Ni_2_^II,II^ complexes display phosphorescence (τ=2.6–8.6 μs) in the NIR (800–1400 nm) spectral region in 2‐MeTHF and in the solid state at 77 K, which is insensitive to π‐conjugation of the coordinated [C_NHC_ C_phenyl_ C_NHC_] ligand. Combined with DFT calculations, the NIR emission is assigned to originate from the ^3^dd excited state. Studies have found that the dinuclear Ni_2_^II,II^ complex can sensitize the formation of singlet oxygen and catalyze the oxidation of cyclo‐dienes under light irradiation.

## Graphical abstract

Dinuclear pincer‐type Ni(II) complexes bridged by formamidinate/α‐carbolinato ligands and the dinuclear Pt(II) and Pd(II) analogues were studied. These Ni(II) complexes show short Ni−Ni distances, resulting in a singlet metal‐metal‐to‐ligand charge transfer transition. At 77 K, they show phosphorescence (2.6–8.6 μs) in the NIR (800–1400 nm) spectral region in 2‐MeTHF and in the solid state, which originates from the ^3^dd excited state as supported by DFT calculations.

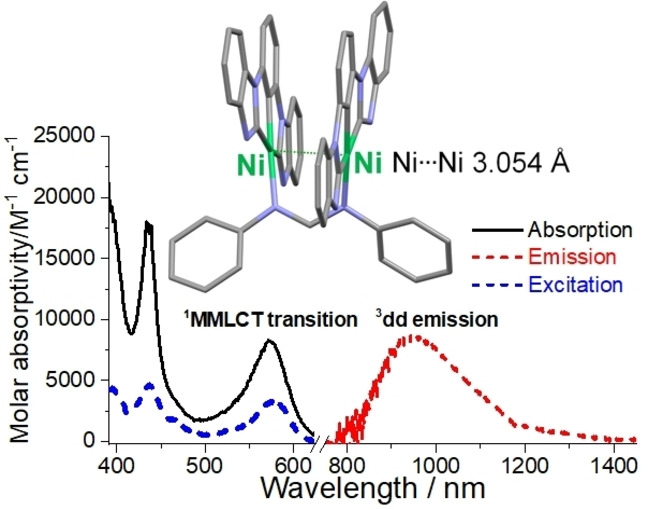

## Introduction

**Figure 1:**
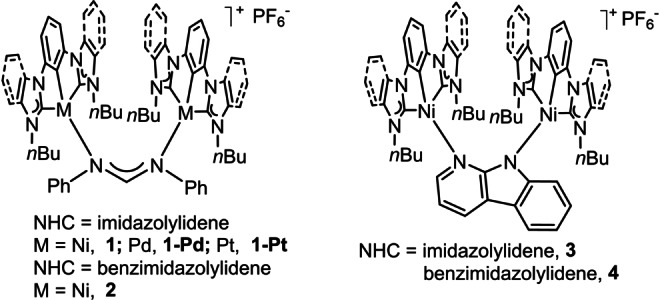
Chemical structures of complexes studied herein.

In recent years, there has been a surge of interest in developing photo‐functional/photoactive molecular materials based on earth‐abundant metals, such as first‐row transition metals, to alleviate the reliance on noble metals in technologies using luminescent transition metal complexes.[^1^, ^2^, ^3^, ^4^, ^5^] However, successful examples of such luminescent molecular materials are largely confined to Cu^I^ complexes with a d^10^ electronic configuration,[^6^, ^7^, ^8^, ^9^, ^10^, ^11^] and more recently, Cr^III^ complexes that exhibit spin‐flip emission.[^3^, ^12^, ^13^, ^14^, ^15^] Challenges in this area of research, such as development of luminescent Fe^II^ and Ni^II^ complexes, are often attributed to the presence of low‐lying metal‐centered (MC) dd excited states of first‐row transition metal complexes,^[5]^ which provides an efficient non‐radiative decay channel for excited states through significant metal‐ligand bond elongation, so that first‐row transition metal complexes are usually non‐emissive in the UV/Visible spectral region. We note that sporadic Ni^II^ complexes, for instance, Ni^II^ benzitripyrrine and Ni^II^ Schiff base complexes,[^16^, ^17^] have been reported to exhibit ligand‐centered fluorescence at room temperature/77 K and the excitation spectrum (if provided) show partial overlap with the corresponding absorption spectrum of the Ni^II^ complexes. However, in some cases, the excitation spectra match the absorption spectra of the free ligand instead, casting doubt on the origin of the emission.[^16^, ^18^] Wenger and co‐workers recently reported a class of cyclometalated pincer‐type Ni^II^ complexes which bear isocyanide as ancillary ligands.^[19]^ Ultrafast time‐resolved spectroscopic measurements show that the complex with the sterically bulky terphenyl isocyanide ligand exhibits a triplet metal‐to‐ligand charge transfer (^3^MLCT) excited state with a lifetime of 48 ps, and the ^3^MLCT excited state relaxes to the ^3^MC dd excited state. Regarding the MC dd excited states of Ni^II^ complexes, we note that [Ni(CN)_4_]^2−^,^[20]^ with four strong‐field cyanide ligands, shows the lowest‐lying MC ^1^A_1g_→^3^B_1g_ transition at ≈550 nm, which implies phosphorescence of the Ni^II^ complex in the deep‐red to NIR region. Indeed, cyclometalated Fe^II^ complexes have been reported to show detectable emission with an emission peak maximum (λ_max_) at ≈1200 nm.^[21]^ Our past efforts to develop Pd^II^ emitters that exhibit strong blue phosphorescence suggest that the use of strong σ‐donors such as carbanions and NHCs (NHC=*N*‐heterocyclic carbene) as ligands may be beneficial in the pursuit of luminescent Ni^II^ complexes by increasing the energy level of the MC dd excited state.^[22]^ Nonetheless, as reported in our previous work on [Ni(O C C O)] complexes^[23]^ and the work of Wenger mentioned above,^[19]^ even using strong‐field chelating ligands (for example, containing two/three carbon‐donor atoms), the resulting Ni^II^ complex remains non‐emissive at room temperature and 77 K. Inspired by the successful phosphorescence turn‐on of dinuclear Pd^II^ complexes with close intramolecular M⋯ M contacts,[^24^, ^25^, ^26^] we prepared dinuclear Ni^II^ complexes in which the bridging ligand supports the close intramolecular Ni⋯ Ni distance and has a chelating pincer carbene ligand to increase the dd excited state energy level. Here, we report the synthesis and characterization of a series of dinuclear Ni^II^ complexes (Figure 1). Notably, all these complexes exhibit phosphorescence in the NIR spectral region at 77 K.

## Results and Discussion

### Synthesis and Characterization

**Figure 2:**
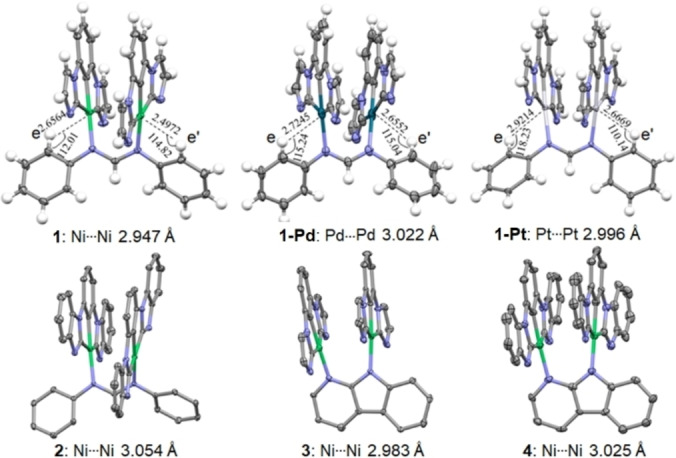
Top: Perspective view of crystal structure of **1** (left), **1**‐**Pd** (middle) and **1**‐**Pt** (right) with the metal‐H distances (Å), metal‐H−C angles (°) and metal‐metal distances (Å) shown. Bottom: Perspective view of crystal structure of **2**, **3** and **4** with their Ni⋯ Ni distances shown. The butyl chains on the ligands and the anions are not shown for clarity in these structures.

The [CCC−M‐X] (“M” stands for metal, “X” stands for halide) complex was prepared following literature procedures.[^27^, ^28^, ^29^] We used deprotonated *N*,*N’*‐diphenylformamidine (NN) and α‐carboline (Cb) as bridging ligands because these ligands can support short metal‐metal contacts (<3 Å) of dinuclear metal complexes, which favors low‐lying metal‐metal bonded excited states.[^30^, ^31^, ^32^] The corresponding CCC−Ni−Cl complex was treated with *N*,*N’*‐diphenylformamidine and KO*t*Bu, and then metathesized with NH_4_PF_6_ to give complexes **1** and **2** with yields of 65–68 %. Attempts to prepare dinuclear α‐carbolinato‐bridged Ni^II^ complexes **3** and **4** in one step using CCC−Ni−Cl gave a mixture of mononuclear and dinuclear complexes that was difficult to purify. Alternatively, dinuclear α‐carbolinato‐bridged complexes were prepared by treating CCC−Ni−Cb with [CCC−Ni−NCCH_3_]PF_6_, affording **3** and **4** in 69 % and 48 % yields, respectively (Figure S1). Dinuclear Pd^II^ and Pt^II^ analogues (**1**‐**Pd** and **1**‐**Pt**) were prepared by treating the corresponding [CCC−M‐X] complexes with *N,N’*‐diphenylformamidine, base, and NH_4_PF_6_ (for M=Pd: base=KO*t*Bu, X=Br, yield=14 %; for M=Pt: base=K_2_CO_3_, X=Cl, yield=68 %). All these dinuclear complexes are stable in CH_3_CN for at least one month. They are stable in solid state in air in the absence of light at room temperature (rt). Dinuclear Ni^II^ complexes **1** and **2** are orange‐red solids, **1**‐**Pd** and **1**‐**Pt** are pale yellow and yellow solids, respectively. ^1^H NMR spectra of **1** recorded at different temperatures (−35 to 25 °C) show a slight down‐field shift on cooling, while **1**‐**Pd** and **1**‐**Pt** show obvious changes. The broad NMR signals of **1**‐**Pd** observed at δ=7.60 ppm and **1**‐**Pt** at δ=8.01 ppm at room temperature become sharper and shift down‐field on cooling. This may be the result of enhanced intramolecular metal⋯ H−C interaction (Figure S2–S4).^[33]^ At 25 °C, the protons e and e’ of the NN ligand of **1** (Figure 2) are particularly down‐field (δ=8.82 ppm) compared to **1**‐**Pt** (δ=8.01 ppm) and **1**‐**Pd** (δ=7.60 ppm) (Figure S2–S6). This finding can be related to the strength of the M⋯ H−C interaction, whose order is Ni^II^>Pt^II^>Pd^II^, which is the same as previously reported.^[34]^ The photostability of the dinuclear Ni^II^ complex was examined in degassed acetonitrile (Figure S7). Upon LEDs irradiation for 30 hours (**1** and **3**, 500 nm (6 W); **2** and **4**, 550 nm (6 W)), the absorption of **1** and **3** in CH_3_CN decreased by 6–8 %, while the absorption of **3** and **4** decreased by 12–13 %. The X‐ray crystal structures of these dinuclear metal complexes are shown in Figure 2,^[36]^ and more information can be found in Table S1–S7.

All Ni atoms in these structures exhibit distorted planar coordination geometries with C_NHC_−Ni−C_NHC_ angles of 158.94–161.53°. Ni−C_NHC_ and Ni−C_phenyl_ bond lengths are 1.896–1.968 Å and 1.827–1.860 Å, respectively, which are comparable to the corresponding distances (1.879–1.954 Å and 1.850–1.870 Å) of mononuclear [CCC−Ni−X]^n+^ (X=Cl, CH_3_, or (trimethylsilyl)methyl, *n*=0; X=NCCH_3_ or CNAr, *n*=1).[^19^, ^28^, ^37^] For the dinuclear Ni^II^ complexes, the two [CCC−Ni] moieties are almost parallel to each other and the intramolecular Ni⋯ Ni distance is short (2.947–3.054 Å), indicating the presence of intramolecular metal‐metal interactions. The crystal structures of **1**‐**Pd** and **1**‐**Pt** are similar to that of **1**, and the intramolecular Pd⋯ Pd and Pt⋯ Pt distances are 3.022 and 2.996 Å, respectively. Short Ni⋯ H distances (2.4972 and 2.6564 Å) and Ni−H−C angles of 114.82° and 112.01° were observed in the crystal structure of **1** (Figure 2, top left), indicating the presence of anagostic Ni⋯ H interactions. For dinuclear Pd^II^ and Pt^II^ complexes, the corresponding M⋯ H distances are longer (for **1**‐**Pd**: 2.6552 and 2.7245 Å; for **1**‐**Pt**: 2.6669 and 2.9214 Å; Figure 2 top middle and right).

### Electrochemistry

**Figure 3:**
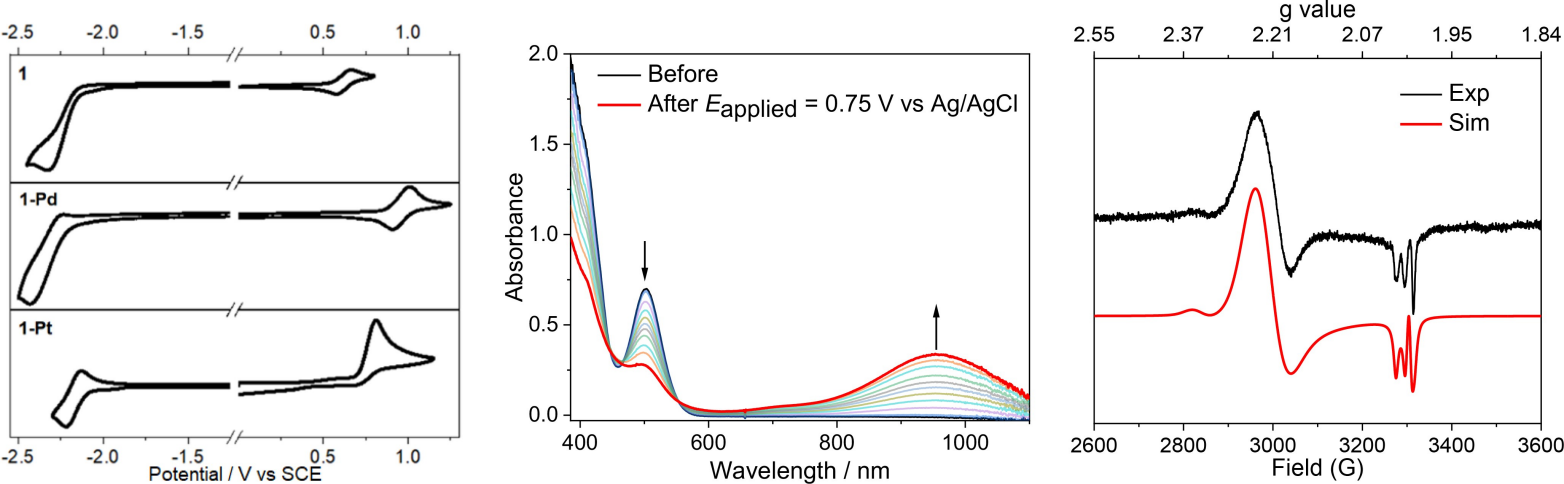
Left: Cyclic voltammograms of **1**, **1**‐**Pd** and **1**‐**Pt** measured in CH_3_CN with [*n*Bu_4_N]PF_6_ (0.1 M) as supporting electrolyte at a scan rate of 100 mV/s; *E*_1/2_(Me_10_Fc^+/0^) range from −0.111 to −0.105 V vs. SCE. *E*_1/2_(Me_10_Fc^+/0^)=−0.51 V vs. *E*_1/2_(Cp_2_Fe^+/0^) in CH_3_CN. Middle: Spectro‐electrochemical study of oxidation of **1** in CH_3_CN with 0.1 M [*n*Bu_4_N]PF_6_ as electrolyte (concentration of **1**≈9.1×10^−4^ M, path length=0.167 cm) at rt. Right: Experimental (black) and simulated (red) X‐band EPR spectra of the reaction mixture of **1** (1×10^−3^ M) and AgBF_4_ in frozen dichloromethane at 100 K, freq. = 9.2779 GHz; another axial signal at g_⊥_=2.342, g_∥_=2.008, with ~4 % concentration of **1^+^**, attributed to side‐product species **II**, is also discernible.

The cyclic voltammograms of **1**, **1**‐**Pd** and **1**‐**Pt** are shown in Figure 3, and the cyclic voltammograms of other complexes are shown in Figure S8. Both dinuclear Ni^II^ complexes **1** and **3** with ^Im^CCC ligand show irreversible reduction waves with E_pc_ at −2.33 to −2.16 V vs SCE. On the other hand, the reduction of complexes **2** and **4** (with ^BIm^CCC ligands) is reversible with *E*_1/2_ at −1.75 to −1.69 V (vs SCE). Complex **1**‐**Pd** shows an irreversible reduction wave with E_pc_ at −2.43 V while complex **1**‐**Pt** shows a reversible reduction couple with *E*_1/2_ at −2.18 V. All these reduction signals are attributed to the reduction of CCC ligands. For oxidation, reversible couples were observed for dinuclear Ni^II^ complexes, with *E*_1/2_ ranging from 0.62 to 0.65 V for **1** and **3**, and from 0.73 to 0.86 V for **2** and **4**. The oxidation potential of **1**, **1**‐**Pd** and **1**‐**Pt** shows anodic shift following the order: Ni (*E*_1/2_=0.62 V)<Pt (irreversible wave; E_pc_=0.81 V)<Pd (reversible couple; *E*_1/2_=0.94 V). UV/Visible spectro‐electrochemical spectra, which show the spectral changes related to the oxidation of complexes **1**–**4**, were obtained (Figure 3 for **1**, Figure S10–S12 for **2**–**4**). The absorption spectra produced by the electrochemical oxidation of these dinuclear Ni^II^ complexes show a broad band peaking at ≈950 nm (see Figure 3, middle, for the oxidation of **1** to **1^+^**). NIR‐absorption of the oxidation product of **1** shows the formation of mixed valence Ni_2_^II,III^ species. The frozen solution EPR spectrum (at 100 K) of complex **1** oxidized with AgBF_4_ in CH_2_Cl_2_ at −78 °C shows an axial signal of g_⊥_=2.225, g_∥_=2.012 (Figure 3, right). Assuming super‐hyperfine splitting arising from one ^14^N nucleus with A_∥_=21 gauss, a nearly 1 : 1 : 1 triplet feature at g_∥_=2.012 can be fitted. This fitting parameter indicates that at the EPR time scale and temperature of 100 K, the oxidized complex is best described as a valence trapped species. This result is consistent with the NIR absorption feature showing **1^+^** as a Class II species (already at ambient temperature).[^38^, ^39^] A minor signal from an unidentified organic radical slightly distorts the line shape at g=2.0043. For more details about EPR results, see the Supporting Information.

### Absorption Spectroscopy and ^1^MMLCT Transitions

**Figure 4:**
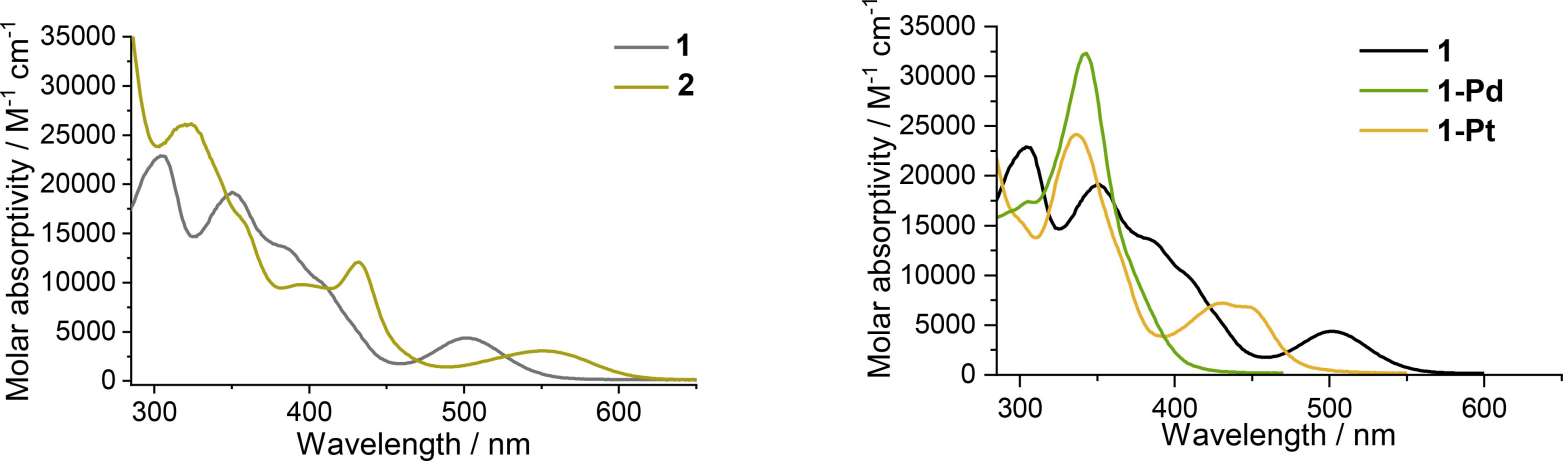
Left: UV/Vis absorption spectra of **1** and **2** in CH_3_CN at rt. Right: UV/Vis absorption spectra of **1**, **1**‐**Pd** and **1**‐**Pt** in CH_3_CN at rt.

The photo‐physical properties of the dinuclear complexes are summarized in Table 1, and the absorption spectra of complexes **1** and **2** are shown in Figure 4. Complexes **1** and **2** show strong absorption bands (ϵ=1.5–3.5×10^4^ M^−1^ cm^−1^) at 285–330 nm (band III) and strong absorption bands/shoulders at 410–440 nm (ϵ=0.97–1.2×10^4^ M^−1^ cm^−1^; band II). In addition, they show low‐intensity broad absorption bands (band I) at ≈500 nm and 550 nm, respectively. Band III is also observed in free CCC ligands (Figure S14), and is therefore attributed to intraligand (IL) ππ* transitions of CCC ligands. Band II is absent in free ligand but is also observed in a wavelength region similar to that of the mononuclear counterpart (CCC−Ni−Cl complex; Figure S14). Thus, this band is tentatively assigned to the d(Ni)→π*(CCC) ^1^MLCT transition. This spectral assignment is consistent with the absorption spectral data of **1**‐**Pd**, where the absorption is not significant at wavelengths above 400 nm and the MLCT transition of the Pd(II) complex occurs at higher energy levels. In addition, complexes **1** and **2** have additional bands at 330–400 nm (Figure 4), which are attributed to transitions involving bridging NN ligand molecular orbitals (MOs).

**Table 1:** Photophysical properties of the metal complexes.

Complex	Absorption λ_abs_/nm (*ϵ*/10^3^ M^−1^ cm^−1^)^[a]^	Emission			
		CH_3_CN (rt)	2‐MeTHF (77 K)	Solid state (rt)	Solid state (77 K)
		λ_em_/nm (τ/μs; Φ)^[b]^	λ_em_/nm (τ/μs)^[c]^	λ_em_/nm (τ/ μs)	λ_em_/nm (τ/ μs)
**1**	306 (22.8), 352 (19.1), 385 (13.5, sh), 410 (9.7, sh), 501 (4.4)	Non‐emissive	1020 (2.6)	Non‐emissive	1030 (−)^[d]^
**1**‐**Pd**	306 (17.5), 344 (32.7), 370 (11.8, sh)	489 (12.9, 0.2 %)	478 (2630), 509 (2620), 550 (2940)	484 (8.6)	480 (131), 515 (110), 551 (110)
**1**‐**Pt**	337 (24.5), 365 (11.9, sh), 429 (7.5), 448 (7.1, sh)	584 (2.3; 68 %)	580 (3.1)	558 (1.0)	550 (1.9)
**2**	322 (26.1), 357 (16.3, sh), 395 (9.8), 432 (12.1), 550 (3.1)	Non‐emissive	950 (3.5)	Non‐emissive	980 (−)^[d]^
**3**	291 (37.0), 325 (13.7, sh), 415 (11.2), 499 (5.5)	Non‐emissive	960 (7.8)	Non‐emissive	975 (−)^[d]^
**4**	319 (19.1), 360 (3.9, sh), 432 (10.0), 543 (2.5)	Non‐emissive	915 (8.6)	Non‐emissive	900 (−)^[d]^

[a] The absorption spectra of Ni^II^ complexes were recorded in CH_3_CN at 8×10^−5^ M at rt. The absorption spectra of Pd^II^ and Pt^II^ complexes were recorded in CH_3_CN at 2×10^−5^ M at rt. [b] Emission quantum yields (Φ) were measured with [Ru(bpy)_3_](PF_6_)_2_ in degassed CH_3_CN as the reference (Φ=0.062). [c] Ni^II^ complexes at 1×10^−4^ M. [d] The signal is too weak to be detected.

**Figure 5:**
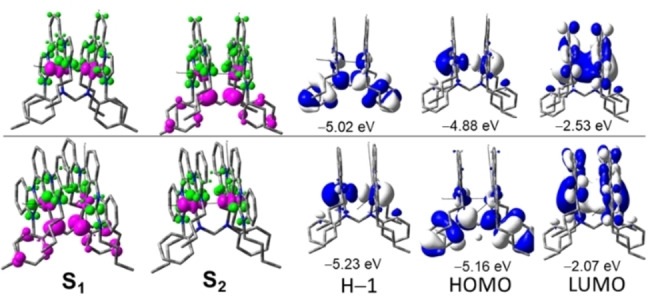
Charge density difference maps (CDDMs; colour code: magenta, decrease in charge density; green, increase in charge density; surface isovalue: 0.025 a. u.) of the S_0_→S_1_ (column 1) and S_0_→S_2_ (column 2) transitions and frontier molecular orbitals (MOs; columns 3–5) of complexes **1** (top) and **2** (bottom) at their respective optimized ground state geometries in THF solution (surface isovalue=0.02 a. u.; orbital energies are also included below the respective MOs).

The lowest‐energy absorption band (band I) of complexes **1** or **2** is not present in the mononuclear Ni^II^ counterparts and is assigned to a singlet metal‐metal‐to‐ligand charge transfer (^1^MMLCT) transition. This assignment is consistent with the energy of band I: with more extended π‐conjugation in the ^BIm^CCC ligand of **2** than that in the ^Im^CCC ligand of **1**, the LUMO of **2** is lower‐lying than that of **1**, and hence, band I is located at a lower energy in **2** (λ~550 nm) than that in **1** (λ~500 nm). Similarly, the lowest‐energy absorption band (band I) for **1**‐**Pd** (λ<420 nm) and **1**‐**Pt** (λ=448 nm) is assigned to the ^1^MMLCT absorption as the energy of this band is consistent with the trend of the oxidation potential, **1**‐**Pd**>**1**‐**Pt**>**1** (Figure 3, left). The absorption spectrum of **1** is insensitive to solvent polarity (shift <4 nm, Figure S15). The spectral assignment of band I is further supported by time‐dependent density functional theory (TDDFT) calculations of complexes **1** and **2** at their respective optimized ground state geometries (note that the PF_6_^−^ counterion was not involved in the calculations throughout). The lowest‐energy absorption bands from the simulated spectra are located at ~500 nm for **1** and ~530 nm for **2** (Figure S27), which are in excellent agreement with the experimental observations. For both complexes, the first absorption band consists of two transitions: S_0_→S_1_ and S_0_→S_2_ transitions; the former is derived from the HOMO→LUMO transition and the latter from the H‐1→LUMO transition. For complex **1**, the HOMO is mainly the anti‐bonding combination between the two Ni($d_{z^{2}}$ ) orbitals (90 %), while the H‐1 is dominantly localized on the bridging NN ligand (74 %) with ~20 % Ni character and is slightly lower in energy (0.14 eV) than the HOMO. For complex **2**, the HOMO and H‐1 are very close in energy and the order is reversed in tetrahydrofuran (THF) solution; i.e., H‐1 is the anti‐bonding combination of the two Ni($d_{z^{2}}$ ) orbitals and the HOMO is NN‐based (Figure 5). For both complexes, the lowest unoccupied molecular orbital (LUMO) is localized mainly on the tridentate CCC ligand (74–76 %), with a minor contribution (20–24 %) from Ni. Therefore, the first absorption band (band I) comes from a combination of ^1^MMLCT and ^1^LLCT/^1^MLCT transitions, with the former being more intense (Table S10 and S13; charge density difference maps for the S_0_→S_1_ and S_0_→S_2_ transitions shown in Figure 5). Further examination of the optimized ^1^MMLCT geometries of **1** and **2** shows that their intramolecular Ni−Ni distances are shrunk by 0.178–0.356 Å relative to their respective optimized ground state geometries in THF solution, which is as expected due to the removal of an electron from anti‐bonding Ni−Ni orbital. The absorption shoulders at ~400 nm (**1**) and ~440 nm (**2**) are derived mainly from the H‐4→LUMO transition for **1** and the H‐5→LUMO transition for **2** where the H‐4 (**1**) and the H‐5 (**2**) are localized on Ni (50–64 %) and CCC ligand (35–47 %; Figure S28 and S29). Since there is no strong interaction between the two Ni ions in the H‐4 and H‐5, this band (~400 nm for **1** and ~440 nm for **2**) is best described as a ^1^MLCT transition.

### fs‐Time‐Resolved Spectroscopy

**Figure 6:**
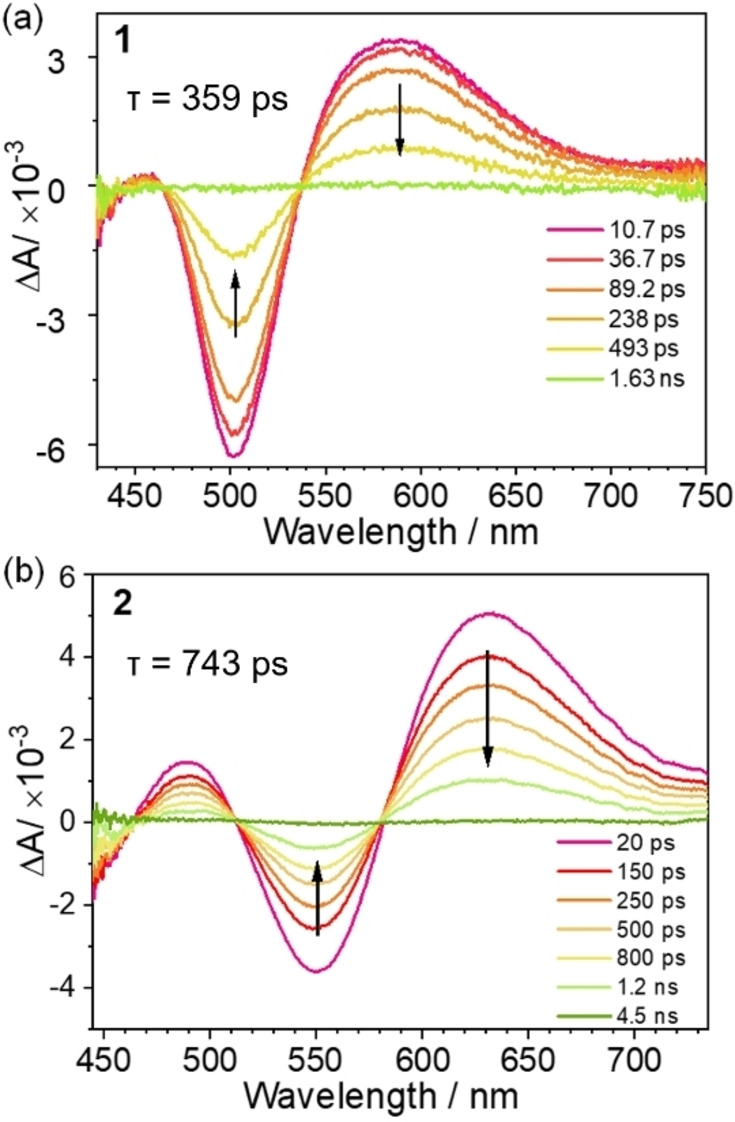
fs‐TA spectrum of (a) **1** in CH_3_CN and (b) **2** in CHCl_3_ at rt.

Femtosecond time‐resolved absorption difference (fs‐TA) and time‐resolved fluorescence (fs‐TRF) spectra were recorded for all dinuclear Ni^II^ complexes in CH_3_CN under 400 nm excitation (Figure 6a and Figure S16–S17). The fs‐TA spectra of dinuclear Ni^II^ complexes **1** and **3** (supported by ^Im^CCC ligand) show a ground state bleaching (GSB) signal at 470–530 nm and a positive excited‐state absorption difference (ESA) signal at 540–750 nm. For complexes **2** and **4** (supported by ^BIm^CCC ligand), their fs‐TA spectra show GSB signals at 520–580 nm and positive ESA signals at 460–510 and 580–750 nm. For NN‐bridged dinuclear Ni^II^ complexes **1** and **2**, the decay of the fs‐TA spectrum is composed of two processes: (i) a fast (1.4–1.9 ps) process, which may be due to stabilization of the excited state via solvent orientation and/or vibrational energy relaxation, and (ii) a slower process (with isosbestic spectral changes), exhibiting a monoexponential decay to baseline with a time constant of 324–359 ps (in CH_3_CN), which is longer than that of Wenger's recently reported mononuclear CCC−Ni complexes with bulky terphenyl isocyanide ligands (up to 133 ps, biexponential; excited state evolution assigned as ^3^MLCT→^3^dd excited state→S_0_).^[19]^ The excited state lifetime of **2** in CHCl_3_ is even longer, with an ESA signal decay time constant of 743 ps at 630 nm (Figure 6b). ESA signals with hundreds of picosecond lifetimes were observed in Ni^II^ porphyrin and phthalocyanine complexes, even as long as 4–7 ns for the ^3^dd excited state of the [Ni^II^(bpy)(Ar)(X)] complex (bpy=2,2’‐bipyridine; Ar=aryl; X=Cl, Br).^[40]^ The ESA signal of the Cb‐bridged dinuclear Ni^II^ complex decays to baseline with a time constant of up to 72 ps (see the Supporting Information for details). fs‐TRF measurements show that dinuclear Ni^II^ complexes **1** to **4** display weak fluorescence with lifetimes of <1 ps (Figure S17), indicating their intersystem crossing rates are >10^12^ s^−1^. Therefore, the fs‐TA ESA signal originates from the triplet excited state. The fs‐TA signals of NN‐bridged and Cb‐bridged complexes with the same CCC ligand are similar. This is not conducive to the fs‐TA signal originating from the ^3^LLCT excited state, which should be affected by the bridging ligand. Compared with the [^Im^CCC−Ni−X]^n+^ (X=CNAr, *n*=1; X=Cl, *n*=0) complex, the ESA signal of the dinuclear Ni^II^ complex is significantly stronger. This finding is different from the ESA signal of the ^3^dd excited state of mononuclear metal complexes reported in the literature, which, to our knowledge, does not exhibit a strong ESA signal.[^19^, ^44^] A significant increase in the excited state lifetime of **2** (from 324 ps in CH_3_CN, to 743 ps in CHCl_3_) was observed when changing the solvent polarity. This is different from the ^3^dd excited state lifetime of the [Ni^II^(bpy)(Ar)(X)] complex, which is not sensitive to solvent polarity.^[43]^ Nonetheless, the isosbestic spectral changes of fs‐TA decay show a state‐to‐state transformation, presumably from a lower energy triplet excited state to the ground state. Our calculations (see below) show that the NIR emission of the dinuclear Ni^II^ complex at 77 K originates from the ^3^dd excited state, and therefore the fs‐TA decay at room temperature can be tentatively attributed to the ^3^dd excited state of the dinuclear Ni^II^ complex. The finding of the strong temperature dependence of the emission lifetime (Figure S21, also see below) can be attributed to the effect of temperature on the non‐radiative decay of the ^3^dd excited state. However, this finding can also be explained by a close‐lying thermally accessible triplet excited state above the ^3^dd excited state. A possible candidate for this close‐lying triplet excited state is the ^3^MMLCT excited state, since the strong ESA signal of complex **1** (Figure 6a) is similar to the fs‐TA signal of the ^3^MMLCT excited state of its dinuclear Pt^II^ analogue **1**‐**Pt** (Figure S16). At this point, this possibility cannot be ruled out.

### Emission and Excited State Reactivity

**Figure 7:**
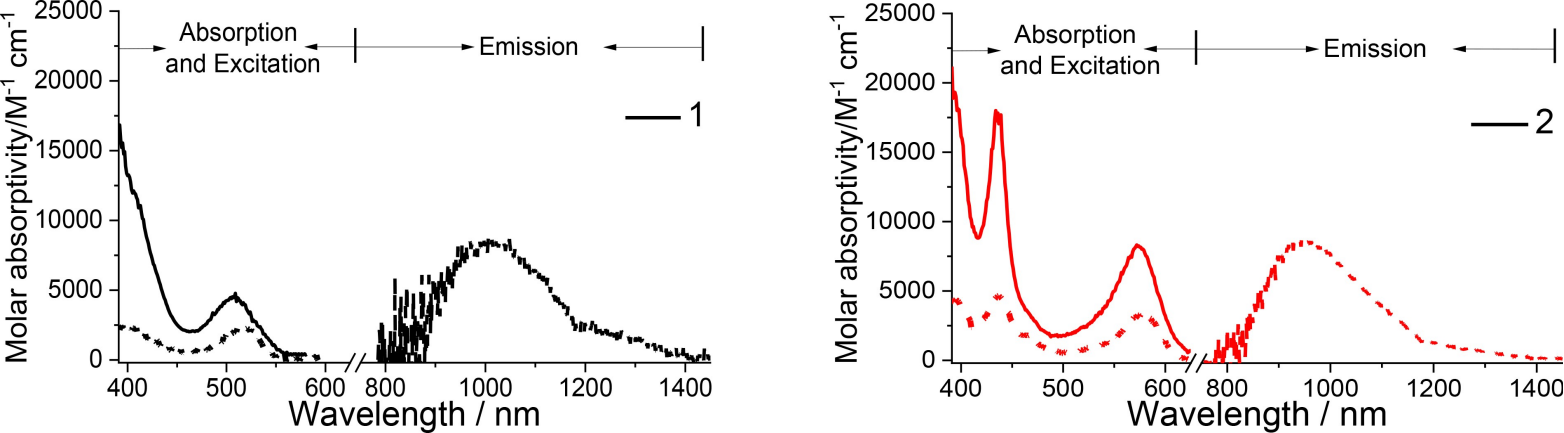
Left: UV/Vis absorption spectra (solid line) of **1** in 2‐MeTHF (10^−4^ M, 173 K), emission (dash line), and excitation spectra (short dash line) of **1** in 2‐MeTHF (10^−4^ M, 77 K). Right: UV/Vis absorption spectra (solid line) of **2** in 2‐MeTHF (10^−4^ M, 173 K), emission (dash line), and excitation spectra (short dash line) of **2** in 2‐MeTHF (10^−4^ M, 77 K).

**Figure 8:**
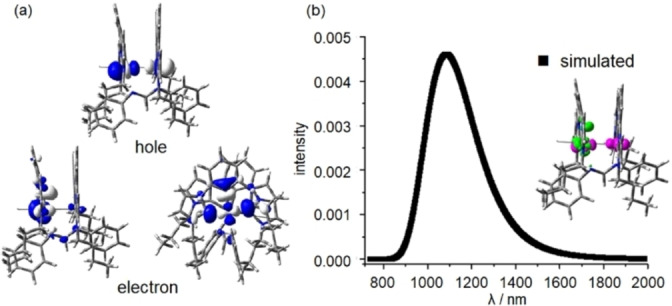
(a) Natural transition orbitals (NTOs; surface isovalue=0.02 a. u.) of the optimized triplet excited state (^3^dd) of **1**; (b) the simulated emission spectrum of **1** based on the optimized ^3^dd excited state in THF; the inset shows the charge density difference map of the ^3^dd excited state (surface isovalue=0.005 a. u.).

Strikingly, at 77 K, these dinuclear Ni^II^ complexes display broad structureless emission in the NIR spectral region (λ_max_=915–1020 nm in 2‐MeTHF; λ_max_=900–1030 nm in solid state), as shown in Figure 7 and S18–S20. The excitation spectrum monitored at emission λ_max_ in glassy solution is similar to the corresponding UV/Visible absorption spectrum recorded at 173 K, confirming that the emission originates from the dinuclear Ni^II^ complex. The emission lifetime at 77 K ranges from 2.6 to 8.6 μs, suggesting that the emission has a triplet parentage. As the temperature increases from 77 K to 108 K, the emission lifetimes of both complexes **1** and **2** shorten (Figure S21) and the emission intensity becomes too weak to be measured at temperatures above 108 K. This finding suggests the presence of a close‐lying, thermally accessible non‐emissive decay pathway/excited state. To examine the nature of the emissive excited state, geometry optimizations of the low‐lying triplet excited states of **1** were performed using DFT/TDDFT methods. The lowest triplet excited state of **1** shows significant elongation of the coordination bonds (0.08–0.14 Å) between one of the Ni ions (Ni2) and the corresponding coordinating atoms of the CCC and NN ligands (Table S18), with more pronounced changes in the peripheral Ni2‐C_NHC_ distances and a noticeable folding of the CCC ligand (the angle ∠C_NHC_−Ni2−C_NHC_ changes from 178° at the optimized ground (S_0_) state structure to 164° at the optimized triplet excited state structure). The structure around the other Ni ion (Ni1) changes only slightly (<0.01 Å), except for one of the Ni1−C_NHC_ distances, which increases from 1.961 Å at the optimized S_0_ state to 1.979 Å at the optimized triplet excited state. Both the bond length and angle distortions in **1** are smaller than those of reported mononuclear [^Im^CCC−Ni−CNAr]^+^ complexes (the ∠C_NHC_−Ni−C_NHC_ change from ~160° (optimized S_0_ geometry) to 142–144° (optimized T_1_ geometry) and the Ni−C_NHC_ distance elongated by 0.094–0.187 Å).^[19]^ Moreover, the change in the torsion, δC_NHC_−Ni−C_Ph_−C_NHC_, is significantly suppressed in **1** (from 175° at the optimized S_0_ geometry to 162° at the optimized triplet excited state geometry in **1**; cf. from 179–180° at the optimized S_0_ geometry to 149–154° at the optimized triplet excited state geometry for the mononuclear [^Im^CCC−Ni−CNAr]^+^ complexes).^[19]^ Furthermore, the Ni1−Ni2 distance of **1** is significantly shortened: from ~2.904 Å (optimized S_0_ geometry) to ~2.764 Å (optimized triplet excited state geometry).The natural transition orbital (NTO) analysis and charge density difference map (Figure 8) of the optimized triplet excited state show that this excited state is mainly a ^3^dd excited state as the “hole” NTO is the anti‐bonding combination of d_z_^2^(Ni)‐orbitals and the “electron”‐NTO is mainly localized on the d_x_^2^−_y_^2^ orbital of Ni2. Figure 8 also depicts the simulated emission spectrum of the ^3^dd excited state of **1** in THF at 77 K; the calculated emission λ_max_ is at ~1085 nm, which is comparable to the emission peak observed (λ_max_=1020 nm) in 2‐MeTHF at 77 K. For more details on the absorption, excitation and emission spectra of dinuclear Ni^II^ complexes, see the Supporting Information.

**Figure 9:**
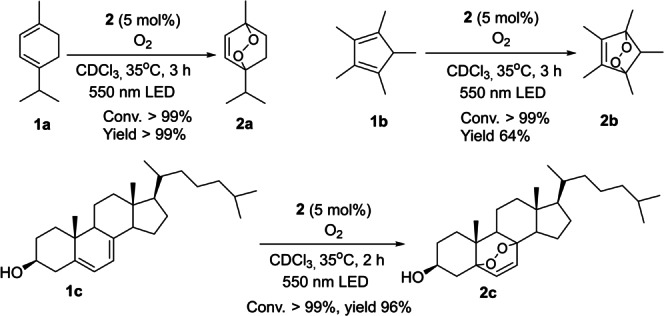
Photo‐induced oxidation of cyclo‐dienes using **2** as a photosensitizer.

The sub‐nanosecond excited state lifetime of the dinuclear NN‐bridged Ni^II^ complex prompted us to examine its excited state reactivity. When the CDCl_3_ solution of **2** was excited at 400 nm in an oxygen atmosphere, an emission signal with λ_max_ at ≈1270 nm was detected, indicating that complex **2** successfully sensitized the formation of singlet oxygen. Using H_2_TPP as a reference, the quantum yield of singlet oxygen (Φ_1O2_) sensitization was estimated to be 0.04 % (Figure S30).^[45]^ Despite the rather low Φ_1O2_, we found that **2** (5 mol %) catalyzed the conversion of cyclo‐dienes to the corresponding endo‐peroxides under 550 nm LED (6 W) irradiation in CDCl_3_ under an oxygen atmosphere.^[46]^ (Figure 9). Control experiments indicate that Ni complex and light are critical for the reaction to occur (except for the reaction of **1** **c** where 9 % yield of **2** **c** was produced in the absence of **2**; Figure S31).

## Conclusions

In summary, we synthesized and structurally characterized a series of dinuclear Ni^II^ complexes with short intramolecular metal‐metal distances. Through electrochemical and chemical oxidation, we also generated and spectroscopically characterized valence‐trapped dinuclear Ni_2_^II,III^ complexes (Class II). These newly synthesized dinuclear Ni^II^ complexes display low‐energy absorption bands that can be assigned to the ^1^MMLCT transition. Comparative study of isostructural Pd(II) and Pt(II) analogues (**1**‐**Pd** and **1**‐**Pt**) shows that the dinuclear Ni^II^ complex has the lowest energy ^1^MMLCT transition. At 77 K, these dinuclear Ni^II^ complexes show emission (lifetime=2.6–8.6 μs) in the NIR spectral region in the solid state and in 2‐MeTHF, which is found to originate from the ^3^dd excited state. The excitation spectrum monitored at the emission wavelength in 77 K glassy solution is similar to the corresponding absorption spectrum, unambiguously confirming that the emission comes from the dinuclear Ni^II^ complex. Using a dinuclear Ni^II^ complex as a photosensitizer, the photo‐oxidation of cyclo‐dienes is achieved by generating singlet oxygen. DFT/TDDFT calculations support absorption assignments for these dinuclear Ni^II^ complexes and suggest the 77 K glassy emission to be of ^3^dd origins. More importantly, as revealed from DFT/TDDFT calculations, the suppressed excited state structural distortion in these dinuclear Ni^II^ complexes, when compared with the reported mononuclear Ni^II^ analogues, is likely the reason why emission could be observed. The findings of this work reveal new structures that reduce excited‐state distortion of Ni^II^ complexes, thereby extending the excited state lifetime and thus enabling photochemical reactivity.

## Conflict of Interests

The authors declare no conflict of interest.

1

## Supporting information

As a service to our authors and readers, this journal provides supporting information supplied by the authors. Such materials are peer reviewed and may be re‐organized for online delivery, but are not copy‐edited or typeset. Technical support issues arising from supporting information (other than missing files) should be addressed to the authors.

Supporting Information

## Data Availability

The data that support the findings of this study are available from the corresponding author upon reasonable request.
