# Supplementary material for: Dinuclear Cyclometalated Pincer Nickel(II) Complexes with Metal‐Metal‐to‐Ligand Charge Transfer Excited States and Near‐Infrared Emission
Source: Angew Chem Int Ed Engl. 2024 Nov 7;64(2):e202414411. doi: 10.1002/anie.202414411 (PMC11720376; doi:10.1002/anie.202414411)
Supplement: Supplementary file 1 — Supporting Information [file ANIE-64-e202414411-s001.pdf]

Angewandte Chemie
Eine Zeitschrift der Gesellschaft Deutscher Chemiker

Supporting Information

Dinuclear Cyclometalated Pincer Nickel(II) Complexes with Metal-Metal-to-Ligand Charge Transfer Excited States and Near-Infrared Emission

*M. Gao, W.-P. To, G. S. M. Tong, L. Du, K.-H. Low, Z. Tang, W. Lu, C.-M. Che**

Supporting Information

©Wiley-VCH 2021

69451 Weinheim, Germany

Dinuclear Cyclometalated Pincer Nickel(II) Complexes with Metal-Metal-to-Ligand Charge Transfer Excited States and Near-Infrared Emission

Mengyue Gao, Wai-Pong To, Glenna So Ming Tong, Lili Du, Kam-Hung Low, Zhou Tang, Wei Lu, and Chi-Ming Che*

Table of Contents

General experimental section S2
Synthesis and characterization S3
Electrochemical studies S16
Photophysical properties S22
DFT calculations S31
Photooxidation of cyclo-dienes S62
References S65
NMR spectra S66

General experimental section

Chemicals and instrumentation

All chemicals were commercially available and used without further purification. Unless otherwise noted, solvents of analytical grade were used for the synthesis. Dry solvent was purchased from J&K Scientific and used as received. Solvents for photophysical measurements and cyclic voltammetric measurements are of HPLC grade. NMR spectra were measured on Bruker Advance 400, DRX-500 or Avance 600M spectrometers; chemical shifts (δ) were reported in ppm with solvent residual peak(s) as internal reference. High resolution electrospray ionization (ESI) mass spectra were recorded on a Bruker Q-Tof Maxis II mass spectrometer. High-resolution EI mass spectra were recorded on a Thermo Scientific DFS high resolution magnetic sector MS. Elemental analyses were conducted at the Institute of Chemistry of the Chinese Academy of Sciences, Beijing. The light source conditions for photosability and photo-induced oxidation reactions: 6 W blue LED (500 or 550 nm, WP-TEC-1020HSL, made in WATTCAS, China).

Photophysical measurements

Ultraviolet-visible (UV-vis) absorption spectra were recorded on a Hewlett-Packard 8453 A diode array spectrophotometer. The spectra were generally obtained with concentration of 8 x 10^{-5} M unless specified. Steady-state emission, excitation spectra and lifetime of samples in glassy sate and solid state were measured via Edinburgh FLS-980 spectrometer equipped with MCP-PMT and NIR-PMT detectors. Femtosecond time-resolved transient absorption (fs-TA) measurements were performed based on a commercial Ti:Sapphire regenerative amplifier laser system (800 nm, 120 fs, 1 kHz, and 3.5 mJ/pulse). A 400 nm excitation wavelength was used for all measurements. The 400 nm excitation pulse was produced from the second harmonic of the 800 nm fundamental laser pulse. In the fs-TA, the samples were probed by a white light continuum pulse created from sapphire pumped by the 800 nm laser. The temporal delay of probe to pump pulse was varied by a computer controlled optical delay line. The fs-TA signals were collected by a monochromator and detected with an air-cooled CCD detector. The instrument response function (IRF) for the fs-TA is ~200-400 fs varying slightly with the spectral wavelength. Femtosecond time-resolved fluorescence (fs-TRF) measurements were performed on the same setup as fs-TA. The output 800 nm laser pulse (200 mW) was used as gate pulse while the 400 nm laser pulse (10 mW) (second harmonic) was used as the pump laser. After excitation by the pump laser, the sample fluorescence was focused into the nonlinear crystal (BBO) mixing with the gate pulse to generate the sum frequency signal. Broadband fluorescence spectra were obtained by changing the crystal angles and the spectra were detected by the air-cooled CCD. For the present experiments, the compound in solution was excited by a 400 nm pump beam (the second harmonic of the fundamental 800 nm from the regenerative amplifier). The solutions were studied in a 2 mm path-length cuvette with an absorbance of 2 at 400 nm throughout the data acquisition.

X-ray crystal structure determination

A Bruker D8 VENTURE Duo FIXED-CHI X-ray diffractometer was used for crystal screening, unit cell determination, and data collection. Crystals suitable for X-ray diffraction were mounted on a MiTeGen dualthickness micro-mounts and placed under a cold nitrogen stream (Oxford). IμS (Mo Kα = 0.71073 Å , 50 kV, 0.8 mA) X-ray source was used. Bruker AXS APEX3 (v2018.7-2) software suite was used for data collection and reduction. Absorption corrections were applied using the program SADABS. Structure solutions were obtained using XT and refined by XL in APEX3. Hydrogen atoms were placed in idealized positions and were set riding on the respective parent atoms. All nonhydrogen atoms were refined with anisotropic thermal parameters. The structure was refined by weighted least squares refinement on F2 to convergence. The X-ray crystallographic coordinates for structures reported in this article have been deposited at Cambridge Crystallographic Data Centre (CCDC), under deposition number CCDC 2234001 for **1**, CCDC 2262899 for **1-Pd**, CCDC 2218392 for **1-Pt**, CCDC 2225768 for **2**, CCDC 2180663 for **3**, and CCDC 2226863 for **4**.

Electrochemical measurements

Cyclic voltammetric measurement was performed on a CH Instruments Electrochemical Analyzer CHI620E using a three-compartment electrochemical cell, with samples containing 0.1 M [$n$$Bu_4$N]$PF_6$ as supporting electrolyte in CH_3CN or CH_2Cl_2 (DCM). The solutions were degassed with argon prior to measurements. Saturated calomel electrode (SCE), glassy carbon, and platinum wire were used as the reference electrode, working electrode, and counter electrode, respectively. All measurements were conducted at room temperature. The ferrocenium/ferrocene ($Cp_2Fe^{+/0}$) couple and decamethylferrocenium/decamethylferrocene ($Me_{10}Fc^{+/0}$) were used as the internal standards.

Spectroelectrochemical measurements

Spectroelectrochemical measurements of **1**-**4** were recorded using "Honeycomb Spectroelectrochemistry Cell Kit" purchased from PINE research. A solution of sample (in CH_3CN, conc. ≈9.1 x 10^{-4} M with 0.1 M [$n$$Bu_4$N]$PF_6$) was added to the cell. Constant potential electrolysis was performed by using a potentiometer. The UV-vis spectral changes of the solution were recorded using the kinetic mode with a time interval of 5 s.

Photostability measurements

The photostability of dinuclear Ni^{II} complexes was examined by UV-vis absorption analysis after light irradiation in a photoreactor. Samples were placed in two-compartment cells consisting of a 10 mL Pyrex bulb and quartz curvette with 1 cm path length. The cells were sealed from the atmosphere with Rotaflo stopcocks. Solutions were degassed in a high-vacuum line by five freeze-thaw-pump cycles.

DFT calculations

In this work, the hybrid density functional, M06L,[1] was employed for all calculations (except otherwise stated) using the program package G16.[2] The basis sets used are 6-31G* [3-4] for main group elements (i.e., H, C, and N) and def2tzvpp for the transition metal, Ni [5] respectively and are obtained from the basis set exchange python library.[6-8] Solvent effect was included by means of the polarizable continuum model (PCM)[9] and default parameters are used for the solvent, tetrahydrofuran (THF). No symmetry constraints were applied in geometry optimizations. The ground state was optimized using the restricted density functional theory (RDFT) method while low-lying triplet excited states were optimized using the unrestricted DFT (UDFT) and TDDFT formalism with the Tamm-Dancoff approximation (TDA)[10] to avoid the triplet instability problems.[11-12] Frequency calculations were performed on all the optimized structures to ensure that they are minimum energy structures by the absence of imaginary frequency (i.e. NImag = 0). Stability calculations were also performed for all the optimized structures to ensure that all the wavefunctions obtained are stable.
Charge density difference maps and Natural Transition Orbitals (NTOs) were obtained using the program Multiwfn [13] and the surfaces are created using the software, GaussView.[14] The absorption spectra and the atom/group contributions (%) to the MOs were obtained using the GaussSum software.[15]

Synthesis and characterization

The [M(C_{NHC}^C_{phenyl}^C_{NHC})X] complexes and NCN ligand were synthesized by following the reported work.[16]

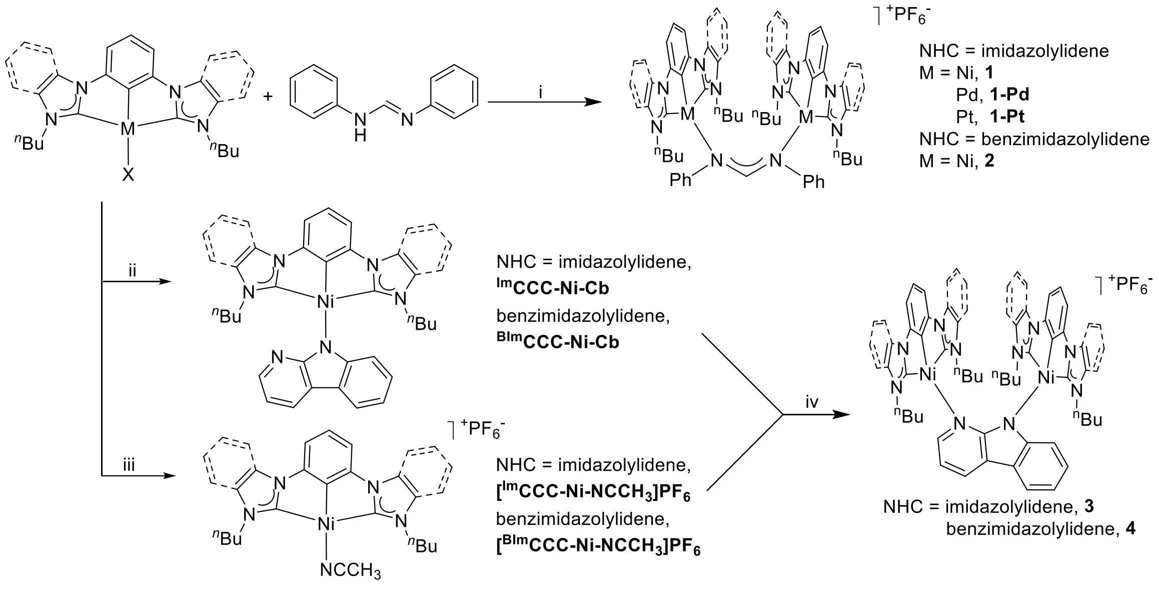

i) For Ni, KO*t*Bu, CH_3CN, NH_4PF_6 room temperature, 6 h; For Pd, X = Br, KO*t*Bu, NH_4PF_6, DMF, reflux, 12 h; For Pt, X = Cl, K_2CO_3, NH_4PF_6, DMF, reflux, 24 h. ii) carboline, KO*t*Bu, THF, room temperature, overnight. iii) $AgPF_6$, CH_3CN, room temperature, 2 h; iv) DCM, room temperature, 6 h.

Figure S1. Synthesis of dinuclear complexes.

Synthesis of 1 [(^{Im}CCC-Ni)$_2$(μ-NN)]PF_6

A mixture of nickel CCC-NHC chloride pincer complex (40.0 mg, 0.08 mmol), NCN ligand (7.8 mg, 0.04 mmol), NH_4PF_6 (excess) and KO*t*Bu (14.0 mg, 0.13 mmol) in dry CH_3CN (4.0 mL), under argon, was stirred for 6 h; then the mixture was filtered. The filtrate was evaporated; the resulting crude product was purified by recrystallization from DCM/Et_2O to give the desired product as a red solid. Yield: 68%. ^{1}H NMR (400 MHz, CD_3CN): δ 8.94 (s, 1H), 8.81 (d, *J* = 8.1 Hz, 2H), 7.35 – 7.23 (m, 6H), 7.11 – 7.00 (m, 6H), 6.83 (t, *J* = 7.7 Hz, 2H), 6.70 – 6.68 (m, 4H), 6.45 (d, *J* = 7.6 Hz, 4H), 3.72 (td, *J* = 12.5, 12.0, 4.9 Hz, 4H), 3.11 – 2.98 (m, 4H), 1.33 – 1.20 (m, 4H), 0.98 – 0.83 (m, 8H), 0.64 – 0.55 (m, 16H). ^{13}C NMR (101 MHz, CD_3CN): δ 174.73, 164.49, 152.62, 148.48, 146.70, 130.29, 129.95, 126.37, 125.13, 123.65, 121.84, 118.51, 115.64, 107.92, 48.86, 34.62, 20.41, 13.85. HR-ESI-MS: [M-PF_6]$^+$ for [$C_{53}H_{61}N_{10}Ni_2$]$^+$, calcd m/z 953.3782, found at m/z 953.3769. Anal. Calcd for $C_{53}H_{61}F_6N_{10}Ni_2P$: C, 57.85; H, 5.59; N, 12.73. Found: C, 57.46; H, 5.58; N, 12.45.

Synthesis of 2 [(^{BIm}CCC-Ni)$_2$(μ-NN)]PF_6

This procedure is the same as synthesis of **1**, except using ^{BIm}CCC-Ni-Cl instead of ^{Im}CCC-Ni-Cl (the synthesis procedure of ^{BIm}CCC-Ni-Cl is the same as ^{Im}CCC-Ni-Cl, except using ^{BIm}CCC ligand instead of ^{Im}CCC ligand). The desired product is a dark red solid. Yield: 65%. ^{BIm}CCC-Ni-Cl ^{1}H NMR (400 MHz, CD_2Cl_2): δ 7.86 (d, *J* = 8.0 Hz, 2H), 7.47 (d, *J* = 8.0 Hz, 2H), 7.43 – 7.30 (m, 4H), 7.22 (s, 3H), 5.00 (t, *J* = 7.8 Hz, 4H), 2.00 – 1.90 (m, 4H), 1.55 – 1.47 (m, 4H), 1.05 – 0.98 (m, 6H). ^{13}C NMR (101 MHz, CD_2Cl_2): δ 148.03, 136.05, 130.32, 125.76, 124.52, 123.36, 111.67, 111.53, 108.36, 46.16, 33.11, 20.51, 14.17.

2 ^{1}H NMR (400 MHz, CD_3CN): δ 9.44 (s, 1H), 8.99 (d, *J* = 8.1 Hz, 2H), 7.66 (d, *J* = 8.5 Hz, 2H), 7.43 (t, *J* = 8.1 Hz, 2H), 7.35 (dd, *J* = 8.2, 2.3 Hz, 4H), 7.30 – 7.22 (m, 6H), 7.18 – 7.07 (m, 6H), 6.95 (td, *J* = 7.9, 2.3 Hz, 2H), 6.82 (dd, *J* = 8.2, 2.3 Hz, 4H), 6.71 – 6.65 (m, 4H), 4.29 – 4.15 (m, 4H), 3.27 (t, *J* = 12.8 Hz, 4H), 1.37 – 1.25 (m, 4H), 0.98 – 0.86 (m, 4H), 0.84 – 0.70 (m, 4H), 0.62 – 0.48 (m, 16H). ^{13}C NMR (101 MHz, CD_3CN): δ 183.94, 164.19, 152.10, 148.20, 147.11, 135.02, 130.59, 130.32, 130.15, 126.74, 126.69, 125.40, 124.39, 124.26, 118.50, 112.48, 111.84, 108.94, 46.82, 32.95, 20.44, 13.85. HRESI-MS: $[M-PF_6]^+$ for $[C_{69}H_{69}N_{10}Ni_2]^+$, calcd m/z 1155.4393, found at m/z 1155.4384. Anal. Calcd for $C_{69}H_{70}F_6N_{10}Ni_2P$: C, 63.67; H, 5.42; N, 10.76. Found: C, 63.51; H, 5.34; N, 10.57.

Synthesis of 3 [(^{Im}CCC-Ni)$_2$(μ-Cb)]PF_6

^{Im}CCC-Ni-Cl (50.0 mg, 0.12 mmol), carboline (20.2 mg, 0.12 mmol) and KO*t*Bu (134.6 mg, 1.20 mmol) were added in dry THF (50 mL) under argon. The mixture was stirred overnight, and then filtered; the filtrate was evaporated to give a crude product, which was purified by recrystallization from DCM/Et_2O to give **^{Im}CCC-Ni-Cb** (yield: 52%). Separately, ^{Im}CCC-Ni-Cl (50.0 mg, 0.12 mmol) and $AgPF_6$ (33.0 mg, 0.13 mmol) were added to dry DCM (20 mL) under argon; then 6 drops of dry CH_3CN were added to the mixture. The reaction mixture was stirred for 2 h at room temperature, and then filtered; the filtrate was evaporated to give crude product **[^{Im}CCC-Ni-NCCH$_3$]PF$_6$** (yield: 84%). A mixture of **^{Im}CCC-Ni-Cb** (22 mg, 0.04 mmol) and crude **[^{Im}CCC-Ni-NCCH$_3$]PF$_6$** (21 mg, 0.04 mmol) was added to dry DCM (20 mL) and stirred for 6 h at room temperature. Solvent was removed under reduced pressure. The crude product was purified by recrystallization from DCM/Et_2O to afford **3** as a red solid. Yield: 69%.

^{Im}CCC-Ni-Cb ^{1}H NMR (500 MHz, CD_2Cl_2): δ 8.42 (dd, *J* = 4.7, 1.8 Hz, 1H), 8.27 (dd, *J* = 7.6, 1.8 Hz, 1H), 8.05 (d, *J* = 7.7 Hz, 1H), 7.98 (d, *J* = 8.1 Hz, 1H), 7.33 – 7.28 (m, 3H), 7.12 (t, *J* = 7.8 Hz, 1H), 7.02 (t, *J* = 7.4 Hz, 1H), 6.88 (dd, *J* = 7.5, 4.7 Hz, 1H), 6.83 (d, *J* = 7.8 Hz, 2H), 6.64 (d, *J* = 2.0 Hz, 2H), 2.69 – 2.58 (m, 4H), 0.90 – 0.83 (m, 4H), 0.32 (t, *J* = 7.3 Hz, 6H), 0.20 – 0.10 (m, 4H). ^{13}C NMR (101 MHz, CD_2Cl_2): δ 174.77, 161.93, 150.16, 149.18, 147.96, 145.58, 126.68, 125.44, 124.66, 124.36, 121.53, 120.34, 118.75, 115.93, 115.54, 113.89, 110.62, 107.00, 48.58, 34.33, 19.51, 13.56. Anal. Calcd for $C_{31}H_{32}N_2Ni$: C, 68.03; H, 5.89; N, 15.35. Found: C, 67.67; H, 5.97; N, 14.88.

3 ^{1}H NMR (500 MHz, CD_3CN): δ 8.87 (d, *J* = 5.3 Hz, 1H), 8.53 (d, *J* = 6.0 Hz, 1H), 8.17 (d, *J* = 7.9 Hz, 1H), 8.14 (d, *J* = 8.2 Hz, 1H), 7.46 (t, *J* = 7.6 Hz, 1H), 7.19 (t, *J* = 7.5 Hz, 1H), 7.14 (dd, *J* = 7.5, 5.4 Hz, 1H), 7.10 (t, *J* = 2.4 Hz, 4H), 6.83 (q, *J* = 8.2 Hz, 2H), 6.58 (s, 2H), 6.54 (d, *J* = 1.9 Hz, 2H), 6.47 (d, *J* = 7.8 Hz, 2H), 6.44 (d, *J* = 7.7 Hz, 2H), 2.73 – 2.64 (m, 2H), 2.46 (td, *J* = 12.1, 5.3 Hz, 2H), 2.33 – 2.25 (m, 4H), 1.50 – 1.33 (m, 4H), 1.09 – 0.87 (m, 8H), 0.80 (t, *J* = 7.3 Hz, 6H), 0.46 – 0.35 (m, 8H), -0.09 – -0.21 (m, 2H). ^{13}C NMR (101 MHz, CD_3CN): δ 175.83, 175.79, 161.53, 147.09, 146.97, 145.58, 128.89, 127.08, 126.12, 125.60, 123.97, 122.43, 122.16, 121.81, 121.56, 118.93, 115.41, 112.51, 107.86, 107.68, 48.80, 35.31, 35.03, 20.32, 19.72, 13.87, 13.66, 1.88, 1.67. Anal. Calcd for $C_{51}H_{57}F_6N_{10}Ni_2P$: C, 57.12; H, 5.36; N, 13.06. Found: C, 57.08; H, 5.37; N, 12.69.

Synthesis of 4 [(^{BIm}CCC-Ni)$_2$(μ-Cb)]PF_6

The procedure is the same as synthesis of **3**, except using **^{BIm}CCC-Ni-Cb** and **[^{BIm}CCC-Ni-NCCH$_3$]PF$_6$** instead of **^{Im}CCC-Ni-Cb** and **[^{Im}CCC-Ni-NCCH$_3$]PF$_6$** (**^{BIm}CCC-Ni-Cb** was difficult to purify; its crude product was directly used). Yield: 48%. ^{1}H NMR (400 MHz, CD_3CN): δ 9.04 (d, *J* = 5.4 Hz, 1H), 8.73 (d, *J* = 7.5 Hz, 1H), 8.30 (d, *J* = 8.0 Hz, 1H), 8.17 (d, *J* = 8.1 Hz, 1H), 7.49 (t, *J* = 7.6 Hz, 1H), 7.35 – 7.25 (m, 6H), 7.16 – 7.09 (m, 4H), 7.05 – 6.98 (m, 2H), 6.97 – 6.88 (m, 4H), 6.81 (d, *J* = 7.8 Hz, 2H), 6.75 (d, 4H), 6.62 (d, *J* = 8.2 Hz, 2H), 3.19 – 3.13 (m, 2H), 2.87 (td, *J* = 12.7, 4.6 Hz, 2H), 2.44 (td, *J* = 12.8, 5.0 Hz, 2H), 2.35 (td, *J* = 12.9, 12.3, 5.8 Hz, 2H), 1.61 – 1.39 (m, 4H), 1.14 – 1.02 (m, 4H), 0.98 – 0.78 (m, 10H), 0.46 – 0.33 (m, 8H), -0.36 – -0.51 (m, 2H). ^{13}C NMR (101 MHz, CD_3CN): δ 184.88, 184.81, 160.97, 150.88, 149.13, 148.12, 147.53, 147.44, 145.71, 134.81, 134.69, 130.11, 129.98, 129.69, 127.56, 127.53, 127.01, 125.61, 125.34, 124.48, 124.21, 122.73, 121.89, 119.66, 113.33, 111.97, 111.94, 111.42, 111.29, 109.04, 46.49, 46.22, 33.73, 33.70, 20.37, 19.47, 13.90, 13.77. Anal. Calcd for $C_{67}H_{65}F_6N_{10}Ni_2P \cdot H_2O$: C, 62.35; H, 5.23; N, 10.85. Found: C, 62.07; H, 5.01; N, 10.42.

Synthesis of 1-Pd [(^{Im}CCC-Pd)$_2$(μ-NN)]PF_6

^{Im}CCC-Pd-Br (50.0 mg, 0.1 mmol), NCN ligand (9.6 mg, 0.05 mmol) , KO*t*Bu (28.0 mg, 0.25 mmol) and NH_4PF_6 (excess) were added to dry DMF (5.0 mL); the mixture was refluxed under argon and stirred for 12 h. The solvent was removed under reduced pressure. The crude product was purified by column chromatography on Al_2O_3 with DCM/methanol (v/v = 20:1) as eluent to give the **1-Pd** as a white solid. Yield: 14%. ^{1}H NMR (400 MHz, CD_3CN): δ 9.04 (s, 1H), 7.82 – 7.59 (br, 4H), 7.24 (t, *J* = 7.8 Hz, 4H), 7.18 (s, 4H), 7.02 – 6.91 (m, 4H), 6.82 (s, 4H), 6.64 (d, *J* = 7.8 Hz, 4H), 3.91 (td, *J* = 11.9, 5.8 Hz, 4H), 3.24 (td, *J* = 11.7, 5.1 Hz, 4H), 1.39 – 1.26 (m, 4H), 1.17 – 1.05 (m, 4H), 0.96 – 0.84 (m, 4H), 0.71 – 0.61 (m, 4H), 0.57 (t, *J* = 7.0 Hz, 12H). ^{13}C NMR (151 MHz, CD_3CN) δ 178.74, 164.74, 153.66, 146.83, 145.99, 129.86, 125.19, 123.20, 120.37, 116.60, 109.22, 49.90, 34.51, 20.30, 13.81. HRESI-MS: $[M-PF_6]^+$ for $[C_{53}H_{61}N_{10}Pd_2]^+$, calcd m/z 1051.3171, found at m/z 1051.3122. Anal. Calcd for $C_{53}H_{61}F_6N_{10}PPd_2$: C 53.23, H 5.14, N 11.71. Found: C 53.12, H 5.22, N 12.07.

Synthesis of 1-Pt [(^{Im}CCC-Pt)$_2$(μ-NN)]PF_6

^{Im}CCC-Pt-Cl (20.0 mg, 0.03 mmol), NCN ligand (2.8 mg, 0.015 mmol), K_2CO_3 (4.0 mg, 0.03 mmol) and NH_4PF_6 (excess) were added to dry DMF (5.0 mL). The mixture was refluxed under argon and stirred for 24 h, and then filtered and washed with DCM. Solvent was removed under reduced pressure. The crude product was purified by recrystallization from DCM/Et_2O to afford the **1-Pt** as a yellow solid. Yield: 68%. ^{1}H NMR (400 MHz, CD_3CN): δ 9.42 – 9.28 (m, 1H), 8.02 (br, 2H), 7.35 (br, 6H), 7.22 (s, 4H), 7.09 (t, *J* = 7.4 Hz, 2H), 6.98 (t, *J* = 7.9 Hz, 2H), 6.89 (s, 4H), 6.63 (d, *J* = 7.8 Hz, 4H), 4.02 – 3.90 (m, 4H), 3.39 – 3.27 (m, 4H), 1.41 – 1.30 (m, 4H), 1.19 – 1.08 (m, 4H), 1.00 – 0.88 (m, 4H), 0.77 – 0.54 (m, 16H). ^{13}C NMR (126 MHz, CD_3CN): δ 173.48, 164.66, 153.55, 144.93, 135.97, 124.06, 123.82, 120.57, 117.23, 108.52, 49.78, 34.32, 20.27, 13.79. HRESI-MS: $[M-PF_6]^+$ for $[C_{53}H_{61}N_{10}Pt_2]^+$, calcd m/z 1227.4367, found at m/z 1227.4377. Anal. Calcd for $C_{53}H_{61}F_6N_{10}PPt_2$: C, 46.36; H, 4.48; N, 10.20. Found: C, 46.12; H, 4.48. N, 9.89.

Variable temperature ^{1}H NMR spectroscopy of **1**, **1-Pd** and **1-Pt**

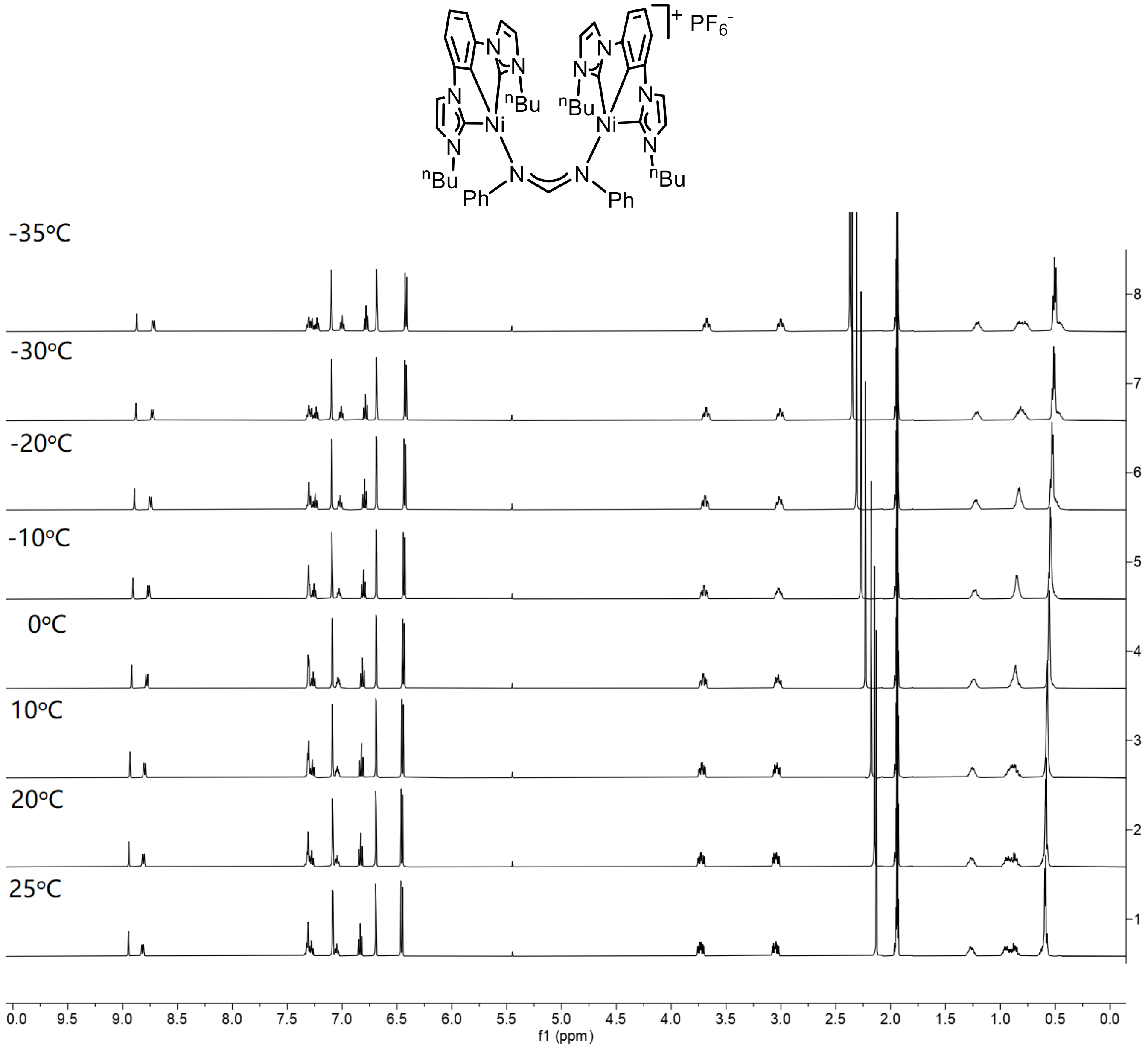

Figure S2. ^{1}H NMR spectra of **1** at various temperatures (500 MHz, CD_3CN).

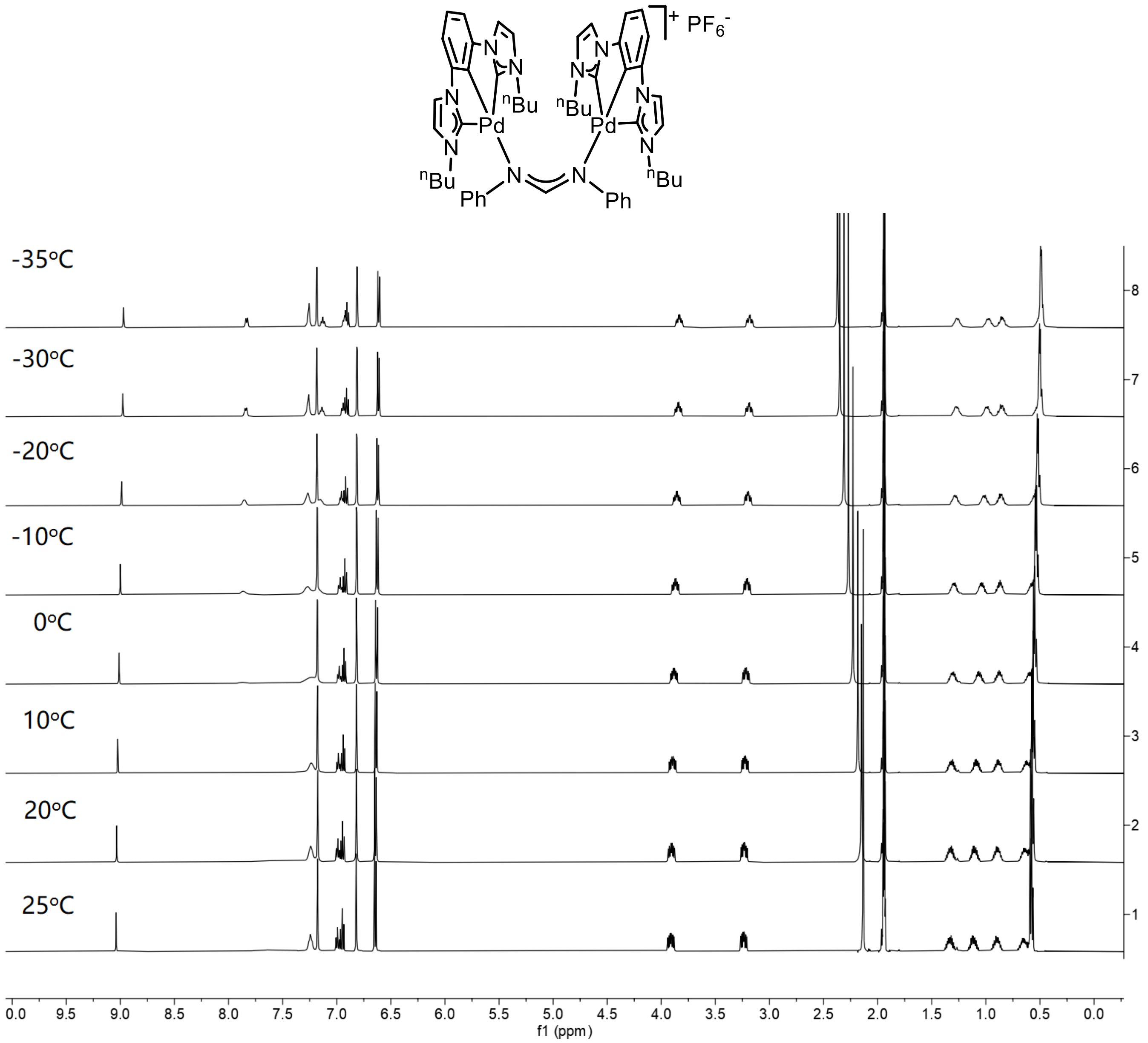

Figure S3. ^{1}H NMR spectra of **1-Pd** at various temperatures (500 MHz, CD_3CN).

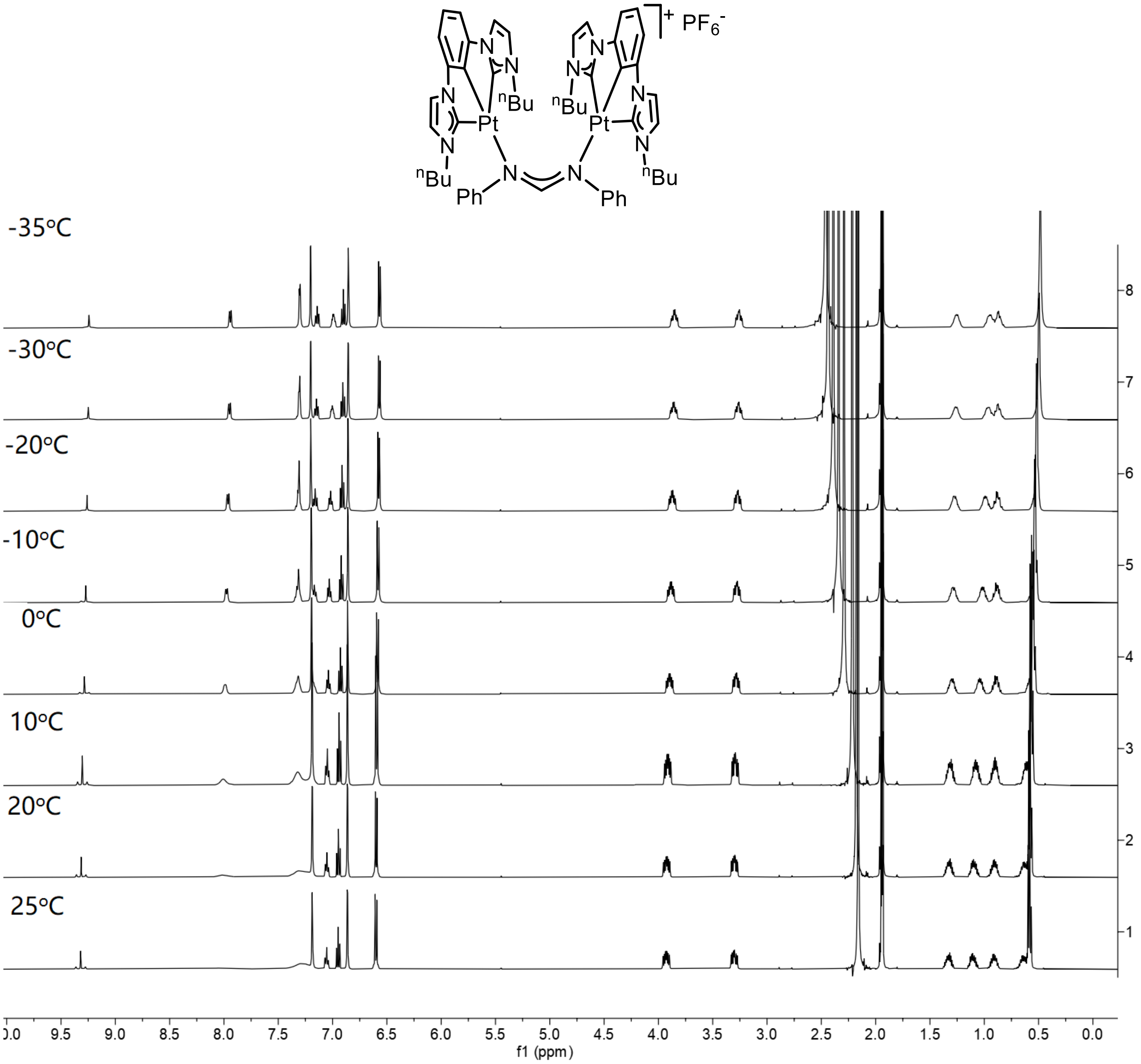

Figure S4. ^{1}H NMR spectra of **1-Pt** at various temperatures (500 MHz, CD_3CN).

2D ^{1}H NMR spectroscopy of 1

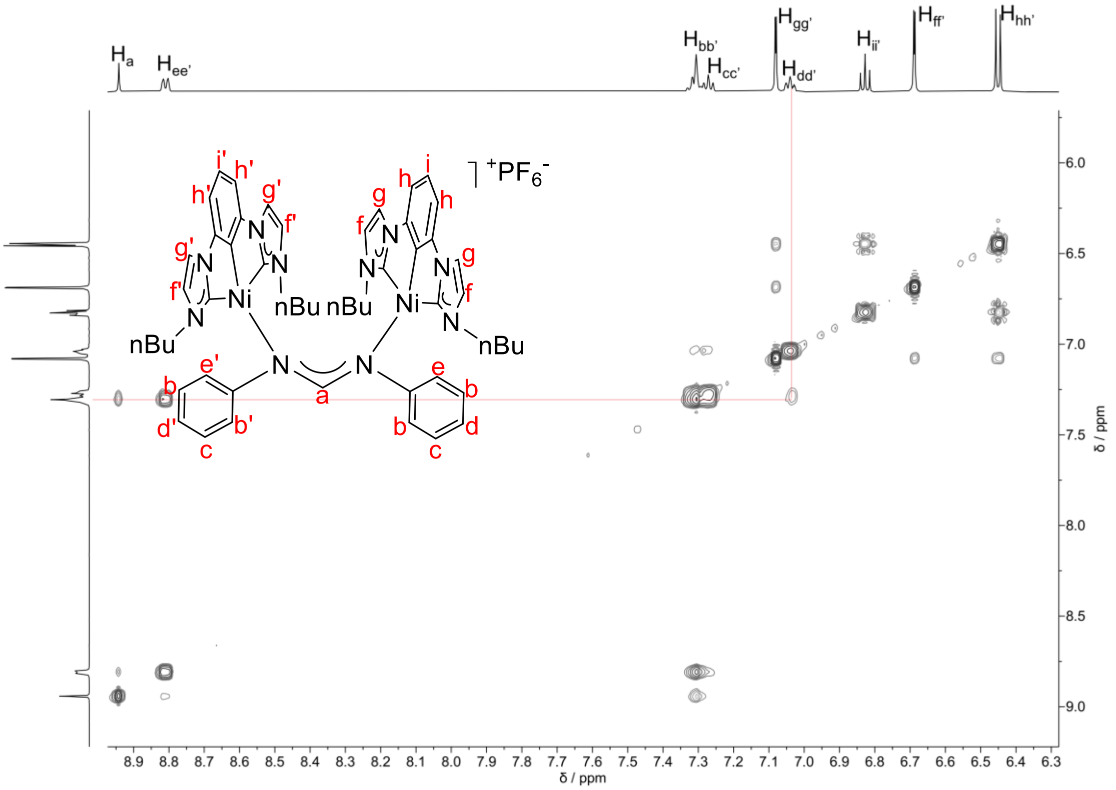

Figure S5 Partial ^{1}H–^{1}H NOESY NMR spectrum of **1** in CD_3CN at 298 K.

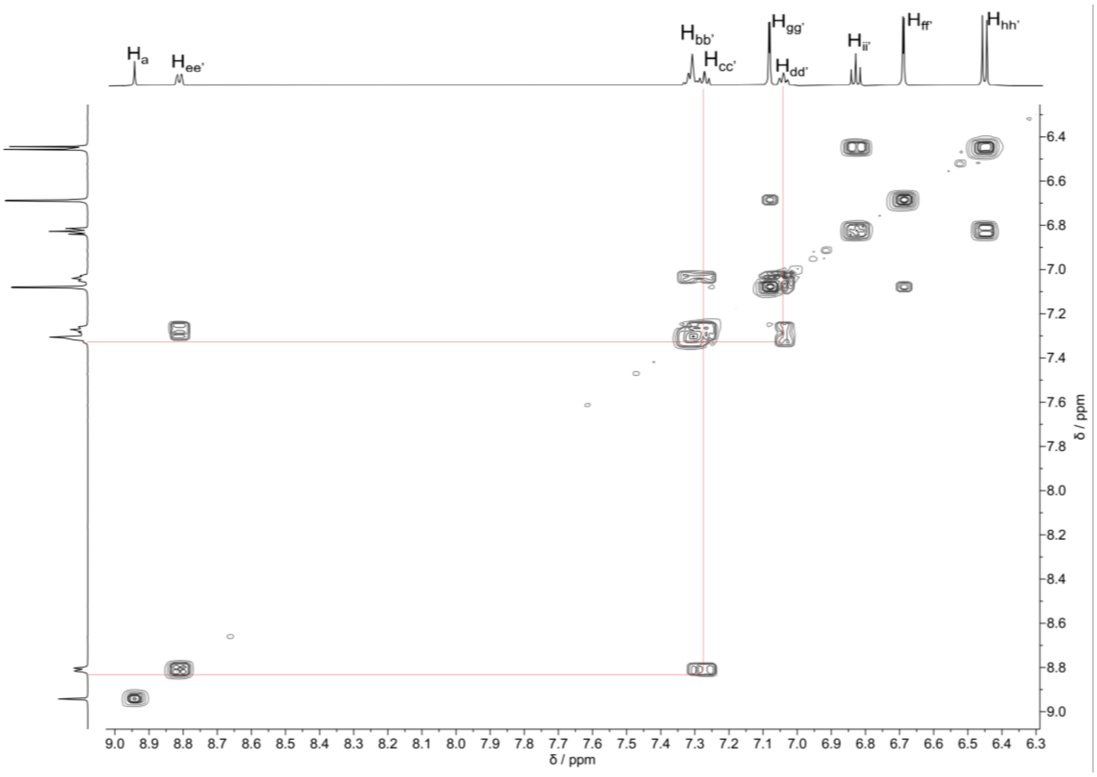

Figure S6 Partial ^{1}H–^{1}H COSY NMR spectrum NMR spectrum of **1** in CD_3CN at 298 K.

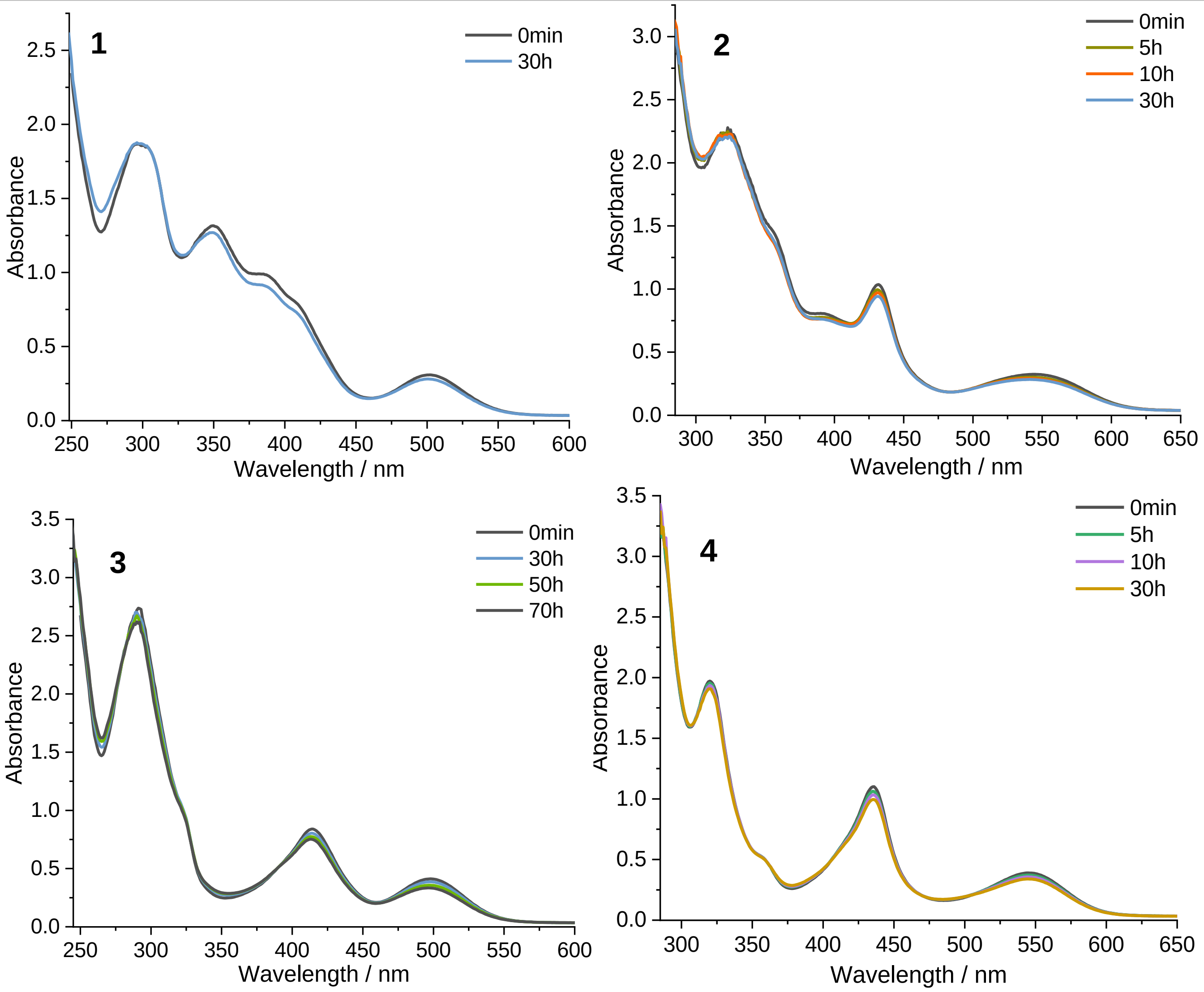

Figure S7. Photostability of dinuclear Ni^{II} complexes tested in degassed CH_3CN, 500 nm (6W) for **1** and **3**, 550 nm (6W) for **2** and **4**.

X-Ray crystallographic data

Table S1. Crystallographic data of dinuclear complexes

Complex	**1**	**2**	**3**	**4**
CCDC	2234001	2225768	2180663	2226863
Empirical formula	4(C53H61N10Ni2)·4(F6P)·2(C7H8)·H2O	C69H69N10Ni2·F6P·3(C2H4Cl2)	C51H57N10Ni2·F6P	C67H65N10Ni2·F6P·C7H8·C2H4Cl2
Formula weight	4604.30	1597.58	1072.45	1463.76
Temperature/ K	173 K	173 K	179 K	173 K
Crystal system	Monoclinic	Monoclinic	Monoclinic	Triclinic
Space group	C2/c	C2/c	$P2_1/n$	*P*1
a / Å	25.009 (3) Å	26.426 (2) Å	12.726 (2)	15.032 (5) Å
b / Å	20.385 (2) Å	15.1019 (13) Å	21.080 (4)	16.515 (6) Å
c / Å	22.734 (3) Å	19.5060 (16) Å	19.040 (3)	16.785 (6) Å
α / °				63.690 (7)°
β / °	104.549 (3)°	109.866 (2)°	103.777 (4)	72.496 (7)°
γ / °				72.496 (7)°
Volumn / $Å^3$	11218 (2)	7321.1 (11)	4961.2 (15)	3431 (2)
Z	2	4	4	2
Radiation	Mo Kα (λ = 0.71073 Å)	Mo *Kα* (λ = 0.71073 Å)	Mo *Kα* (λ = 0.71073 Å)	Mo *Kα* , λ = 0.71073 Å
ρ_{calc}g / cm^3	1.363	1.449	1.436	1.417
F(000)	4812	3312	2232	1524
R_1	0.066	0.046	0.140	0.116
wR_2	0.130	0.097	0.228	0.323
Restrained goodness of fit	1.01	1.02	1.30	1.05

Complex	**1-Pd**	**1-Pt**
CCDC	2262899	2218392
Empirical formula	C53H61N10Pd2·F6P·C6H14	2(C53H61N10Pt2)·2(F6P)·3(C5H12O)
Formula weight	1282.06	3010.96
Temperature/K	173 K	181 K
Crystal system	Monoclinic	Monoclinic
Space group	C2/c	C2/c
a / Å	25.749 (4) Å	34.412 (2) Å
b / Å	20.532 (3) Å	18.3179 (13) Å
c / Å	22.810 (4) Å	21.3690 (13) Å
α / º		
β / º	101.461 (3)°	114.415 (2)°
γ / º		
Volumn / $Å^3$	11818 (3)	12265.7 (14)
Z	8	4
Radiation	Mo *Kα*, (*λ* = 0.71073 Å)	Mo Kα (*λ* = 0.71073 Å)
ρ_{calc}g / cm^3	1.441	1.631
F(000)	5280	5992
R_1	0.074	0.052
wR_2	0.22	0.096
Restrained goodness of fit	0.96	1.02

Table S2. Selected bond lengths (Å) and angles (º) of **1**

Bond lengths (Å)				
Ni1—Ni2 2.9468 (7)	C17—Ni1 1.860 (4)	C23—Ni1 1.948 (4)	N2—Ni2 1.959 (3)	C34—Ni2 1.942 (4)
N1—Ni1 1.971 (3)	C14—Ni1 1.968 (4)	C43—Ni2 1.956 (4)	C37—Ni2 1.857 (4)	

Bond angles (º)			
C37—Ni2—C43 80.50 (16)	C22—C17—Ni1 121.3 (3)	C1—N2—Ni2 123.8 (3)	C37—Ni2—C34 79.99 (17)
C34—Ni2—C43 159.87 (16)	C18—C17—Ni1 121.0 (3)	C8—N2—Ni2 117.1 (2)	N1—Ni1—Ni2 79.64 (9)
C37—Ni2—N2 179.31 (16)	N6—C23—Ni1 143.0 (3)	C17—Ni1—C23 79.76 (17)	C2—N1—Ni1 119.7 (2)
C34—Ni2—N2 100.69 (14)	N5—C23—Ni1 114.8 (3)	C17—Ni1—C14 79.87 (17)	N4—C14—Ni1 114.3 (3)
C43—Ni2—N2 98.82 (14)	N7—C34—Ni2 141.7 (3)	C23—Ni1—C14 158.94 (17)	C14—Ni1—Ni2 104.22 (11)
C37—Ni2—Ni1 100.53 (12)	N8—C34—Ni2 114.9 (3)	C17—Ni1—N1 175.59 (15)	C1—N1—Ni1 123.2 (3)
C34—Ni2—Ni1 84.79 (11)	C38—C37—Ni2 121.2 (3)	C23—Ni1—N1 101.14 (15)	N3—C14—Ni1 143.0 (3)
C43—Ni2—Ni1 103.65 (12)	C42—C37—Ni2 120.9 (3)	C14—Ni1—N1 99.57 (15)	C23—Ni1—Ni2 83.28 (11)
N2—Ni2—Ni1 79.56 (9)	N10—C43—Ni2 142.7 (3)	C17—Ni1—Ni2 96.22 (12)	N9—C43—Ni2 114.3 (3)
N3—C26—C27 110.0 (4)			

Table S3. Selected bond lengths (Å) and angles (º) of **2**

Bond lengths (Å)			
Ni1—C15 1.855 (3)	Ni1—C21 1.929 (3)	Ni1—C8 1.959 (3)	Ni1—N1 1.959 (2)

Bond angles (º)			
C15—Ni1—C21 80.31 (11)	N2—C8—Ni1 140.9 (2)	C20—C15—Ni1 120.7 (2)	N5—C21—Ni1 140.4 (2)
C15—Ni1—C8 80.07 (11)	N3—C8—Ni1 114.49 (18)	C16—C15—Ni1 121.2 (2)	N4—C21—Ni1 115.19 (18)
C21—Ni1—C8 159.77 (11)	C1—N1—Ni1 121.43 (18)	C21—Ni1—N1 100.65 (10)	C8—Ni1—N1 99.15 (10)
C15—Ni1—N1 177.50 (10)	C2—N1—Ni1 120.03 (16)		

Table S4. Selected bond lengths (Å) and angles (º) of **3**

Bond lengths (Å)			
Ni1—C20_1	1.850 (7)	Ni2—C20_2	1.858 (7)
Ni1—N1_3	1.931 (13)	Ni2—N2_4	1.87 (2)
Ni1—C12_1	1.934 (8)	Ni2—C21_2	1.924 (8)
Ni1—C21_1	1.936 (8)	Ni2—C12_2	1.950 (8)
Ni1—N1_4	1.955 (17)	Ni2—N2_3	2.030 (17)
Ni1—Ni2	2.9827 (18)		
Bond angles (º)			
C20_1—Ni1—N1_3	178.9 (7)	N2_4—Ni2—C21_2 99.7 (17)	89.89 (19)
C20_1—Ni1—C12_1	80.2 (3)	C20_2—Ni2—C12_2 79.7 (3)	79.70 (14)
N1_3—Ni1—C12_1	99.1 (9)	N2_4—Ni2—C12_2	100.5 (17)
C20_1—Ni1—C21_1	80.1 (3)	C21_2—Ni2—C12_2	159.6 (3)
N1_3—Ni1—C21_1	100.6 (9)	C20_2—Ni2—N2_3	173.5 (7)
C12_1—Ni1—C21_1	159.8 (3)	C21_2—Ni2—N2_3	103.5 (10)
C20_1—Ni1—N1_4	173.2 (11)	C12_2—Ni2—N2_3	96.8 (10)
C12_1—Ni1—N1_4	105.2 (13)	C20_2—Ni2—Ni1	92.8 (3)
C21_1—Ni1—N1_4	94.7 (13)	N2_4—Ni2—Ni1	85.3 (7)
C20_1—Ni1—Ni2	100.4 (3)	C21_2—Ni2—Ni1	85.3 (3)
N1_3—Ni1—Ni2	78.7 (5)	C12_2—Ni2—Ni1	99.3 (3)
C12_1—Ni1—Ni2	88.0 (3)	N2_3—Ni2—Ni1	82.4 (5)
C21_1—Ni1—Ni2	100.4 (3)	C11_3—N1_3—Ni1	128.4 (9)
N1_4—Ni1—Ni2	76.0 (6)	C1_3—N1_3—Ni1	124.4 (9)
C20_2—Ni2—N2_4	178.1 (8)	C10_3—N2_3—Ni2	121.8 (10)
C20_2—Ni2—C21_2	80.2 (3)	C11_3—N2_3—Ni2	120.1 (9)

Table S5. Selected bond lengths (Å) and angles (º) of **4**

Bond lengths (Å)			
Ni1—C12_1 1.848 (4)	Ni1—C18_1 1.896 (3)	Ni1—N1 1.925 (11)	Ni1—C25_1 1.942 (3)
Ni2—C12_2 1.827 (3)	Ni2—C18_2 1.884 (3)	Ni2—C25_2 1.943 (3)	Ni2—N2 1.947 (11)
**Bond angles (º)**			
C12_1—Ni1—C18_1 80.71 (13)	C12_1—Ni1—N1 179.1 (4)	C18_1—Ni1—N1 99.1 (4)	C12_1—Ni1—C25_1 79.75 (13)
C18_1—Ni1—C25_1 159.65 (17)	N1—Ni1—C25_1 100.5 (4)	C12_2—Ni2—C18_2 81.56 (13)	C12_2—Ni2—C25_2 80.23 (13)
C18_2—Ni2—C25_2 161.53 (15)	C12_2—Ni2—N2 176.7 (4)	C18_2—Ni2—N2 98.9 (3)	C25_2—Ni2—N2 99.4 (3)
C1—N1—Ni1 125.3 (9)	C11—N1—Ni1 131.5 (9)	C10—N2—Ni2 124.0 (10)	C11—N2—Ni2 120.7 (9)
C17_1—C12_1—Ni1 121.9 (2)	C13_1—C12_1—Ni1 120.54 (19)	N3_1—C18_1—Ni1 141.6 (2)	N4_1—C18_1—Ni1 116.28 (18)
N6_1—C25_1—Ni1 141.1 (2)	N5_1—C25_1—Ni1 115.01 (19)	C17_2—C12_2—Ni2 122.1 (2)	C13_2—C12_2—Ni2 120.15 (19)
N3_2—C18_2—Ni2 142.3 (2)	N4_2—C18_2—Ni2 115.58 (19)	N6_2—C25_2—Ni2 141.8 (2)	N5_2—C25_2—Ni2 114.39 (19)

Table S6. Selected bond lengths (Å) and angles (º) of **1-Pd**

Bond lengths (Å)					
Pd1—C22_7 1.825 (3)	Pd1—C23_7 2.051 (3)	Pd1—C14_4 2.080 (3)	Pd1—N1_3 2.148 (7)	Pd2—C22_5 1.932 (9)	Pd2—C23_5 2.055 (9)
Pd1—C14_7 1.998 (3)	Pd1—C23_4 2.076 (3)	Pd1—C22_4 2.084 (3)	Pd1—Pd2 3.0220 (9)	Pd2—C14_5 2.033 (9)	Pd2—N2_3 2.131 (7)

Bond angles (º)			
C22_7—Pd1—C14_7 82.50 (11)	C22_7—Pd1—C23_7 81.27 (11)	C14_7—Pd1—C23_7 163.45 (11)	C23_4—Pd1—C14_4 149.18 (11)
C23_4—Pd1—C22_4 74.91 (10)	C14_4—Pd1—C22_4 74.62 (10)	C22_7—Pd1—N1_3 173.76 (19)	C14_7—Pd1—N1_3 101.85 (18)
C23_7—Pd1—N1_3 94.59 (17)	C23_4—Pd1—N1_3 106.69 (17)	C14_4—Pd1—N1_3 103.94 (17)	C22_4—Pd1—N1_3 177.24 (19)
C22_7—Pd1—Pd2 97.53 (11)	C14_7—Pd1—Pd2 82.91 (11)	C23_7—Pd1—Pd2 102.45 (11)	C23_4—Pd1—Pd2 103.86 (11)
C14_4—Pd1—Pd2 85.30 (11)	C22_4—Pd1—Pd2 98.76 (9)	N1_3—Pd1—Pd2 78.72 (17)	C22_5—Pd2—C14_5 78.9 (4)
C22_5—Pd2—C23_5 77.4 (4)	C14_5—Pd2—C23_5 156.2 (4)	C22_5—Pd2—N2_3 179.1 (3)	C14_5—Pd2—N2_3 101.8 (3)
C23_5—Pd2—N2_3 101.9 (3)	C22_5—Pd2—Pd1 101.1 (2)	C14_5—Pd2—Pd1 84.7 (2)	C23_5—Pd2—Pd1 102.1 (2)
N2_3—Pd2—Pd1 78.44 (19)	C1_3—N1_3—Pd1 122.2 (6)	C2_3—N1_3—Pd1 121.2 (6)	C1_3—N2_3—Pd2 123.7 (6)
C8_3—N2_3—Pd2 118.3 (6)	N3_5—C14_5—Pd2 143.1 (8)	N4_5—C14_5—Pd2 114.1 (6)	N3_4—C14_4—Pd1 141.13 (19)
N4_4—C14_4—Pd1 117.48 (17)	C21_5—C22_5—Pd2 120.7 (8)	C17_5—C22_5—Pd2 120.3 (7)	N6_5—C23_5—Pd2 141.5 (8)
N5_5—C23_5—Pd2 114.9 (7)	C21_4—C22_4—Pd1 121.30 (18)	C17_4—C22_4—Pd1 121.38 (19)	N6_4—C23_4—Pd1 140.33 (19)
N5_4—C23_4—Pd1 116.49 (18)	N3_7—C14_7—Pd1 147.7 (2)	N4_7—C14_7—Pd1 110.95 (17)	C21_7—C22_7—Pd1 122.11 (19)
C17_7—C22_7—Pd1 121.01 (19)	N6_7—C23_7—Pd1 147.5 (2)	N5_7—C23_7—Pd1 109.27 (17)	

Table S7. Selected bond lengths (Å) and angles (º) of **1-Pt**

Bond lengths (Å)			
Pt1—C9	1.957 (7)	Pt2—C29	1.942 (7)
Pt1—C10	2.030 (7)	Pt2—C30	2.039 (7)
Pt1—C1	2.036 (7)	Pt2—C21	2.044 (7)
Pt1—N9	2.139 (5)	Pt2—N10	2.126 (5)
Pt1—Pt2	2.9955 (4)		
Bond angles (º)			
C9—Pt1—C10	78.8 (3)	C29—Pt2—C30	78.5 (3)
C9—Pt1—C1	78.7 (3)	C29—Pt2—C21	78.5 (3)
C10—Pt1—C1	157.3 (3)	C30—Pt2—C21	156.9 (3)
C9—Pt1—N9	176.1 (2)	C29—Pt2—N10	176.6 (2)
C10—Pt1—N9	99.9 (2)	C30—Pt2—N10	101.2 (3)
C1—Pt1—N9	102.7 (2)	C21—Pt2—N10	101.9 (3)
C9—Pt1—Pt2	95.79 (19)	C29—Pt2—Pt1	97.00 (19)
C10—Pt1—Pt2	95.75 (18)	C30—Pt2—Pt1	95.28 (18)
C1—Pt1—Pt2	89.72 (17)	C21—Pt2—Pt1	89.89 (19)
N9—Pt1—Pt2	80.59 (14)	N10—Pt2—Pt1	79.70 (14)

Electrochemical studies

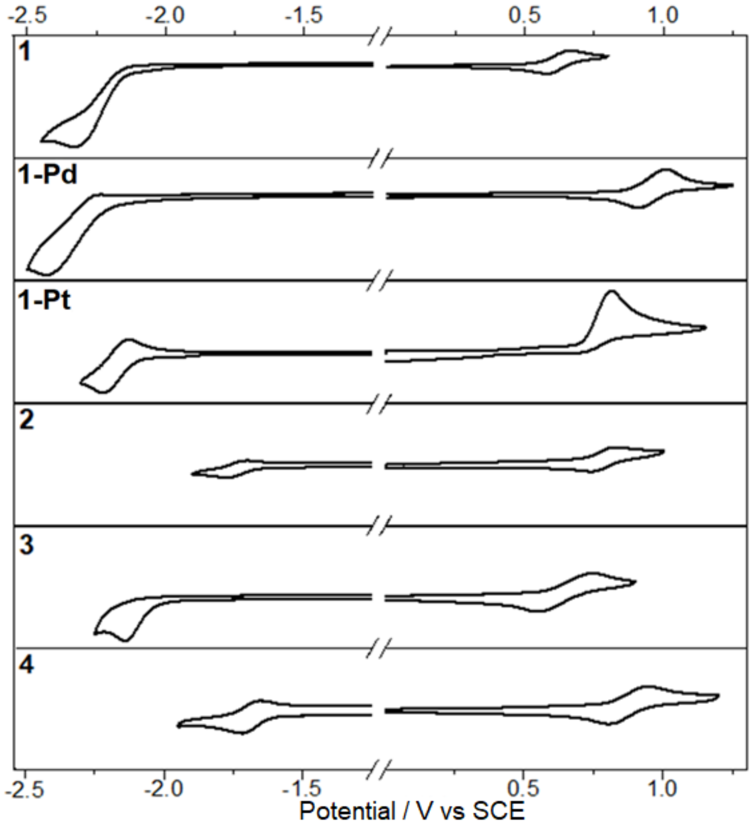

Figure S8. Cyclic voltammograms of **1**, **1-Pd**, **1-Pt** and **2** measured in CH_3CN solution with [$n$$Bu_4N$]$PF_6$ (0.1 M) as supporting electrolyte at a scan rate of 100 mV/s; $E_{1/2}$($Me_{10}Fc^{+/0}$) range from -0.111 to -0.105 V vs. SCE. $E_{1/2}$($Me_{10}Fc^{+/0}$) = -0.51 V vs. $E_{1/2}$($Cp_2Fe^{+/0}$) in CH_3CN. The oxidation waves of **3** and **4** measured in DCM and reduction waves measured in CH_3CN with 0.1 M [$n$$Bu_4N$]$PF_6$ as supporting electrolyte at a scan rate of 100 mV/s; $E_{1/2}$($Me_{10}Fc^{+/0}$) = -0.124 V values vs. SCE. $E_{1/2}$($Me_{10}Fc^{+/0}$) = -0.520 V vs. $E_{1/2}$($Cp_2Fe^{+/0}$) in DCM.

Table S8. Electrochemical properties of complexes.[a]

Complex	$E_{1/2,\ red}$ /V	E_{pc} /V	$E_{1/2,\ ox}$ /V	E_{pa} /V
**1**	-	-2.33	0.62	-
**1-Pd**	-	-2.43	0.94	-
**1-Pt**	-2.18	-	-	0.81
**2**	-1.75	-	0.73	-
**3**	-	-2.16	0.65[b]	-
**4**	-1.69	-	0.86[b]	-

[a] Redox measurements were carried out in CH_3CN solution with 0.1 M [$n$$Bu_4N$]$PF_6$ as supporting electrolyte at a scan rate of 100 mV/s; values *vs*. SCE. $E_{1/2}$($Me_{10}Fc^{+/0}$) = -0.111 to -0.105 V. $E_{1/2}$($Me_{10}Fc^{+/0}$) = -0.51 V vs. $E_{1/2}$($Cp_2Fe^{+/0}$) in CH_3CN. [b] Measured in DCM for oxidation waves with 0.1 M [$n$$Bu_4N$]$PF_6$ as supporting electrolyte at a scan rate of 100 mV/s. $E_{1/2}$($Me_{10}Fc^{+/0}$) = -0.520 V vs. $E_{1/2}$($Cp_2Fe^{+/0}$) in DCM with [$n$$Bu_4N$]$PF_6$ (0.1 M) as supporting electrolyte at a scan rate of 100 mV/s; values *vs*. SCE.

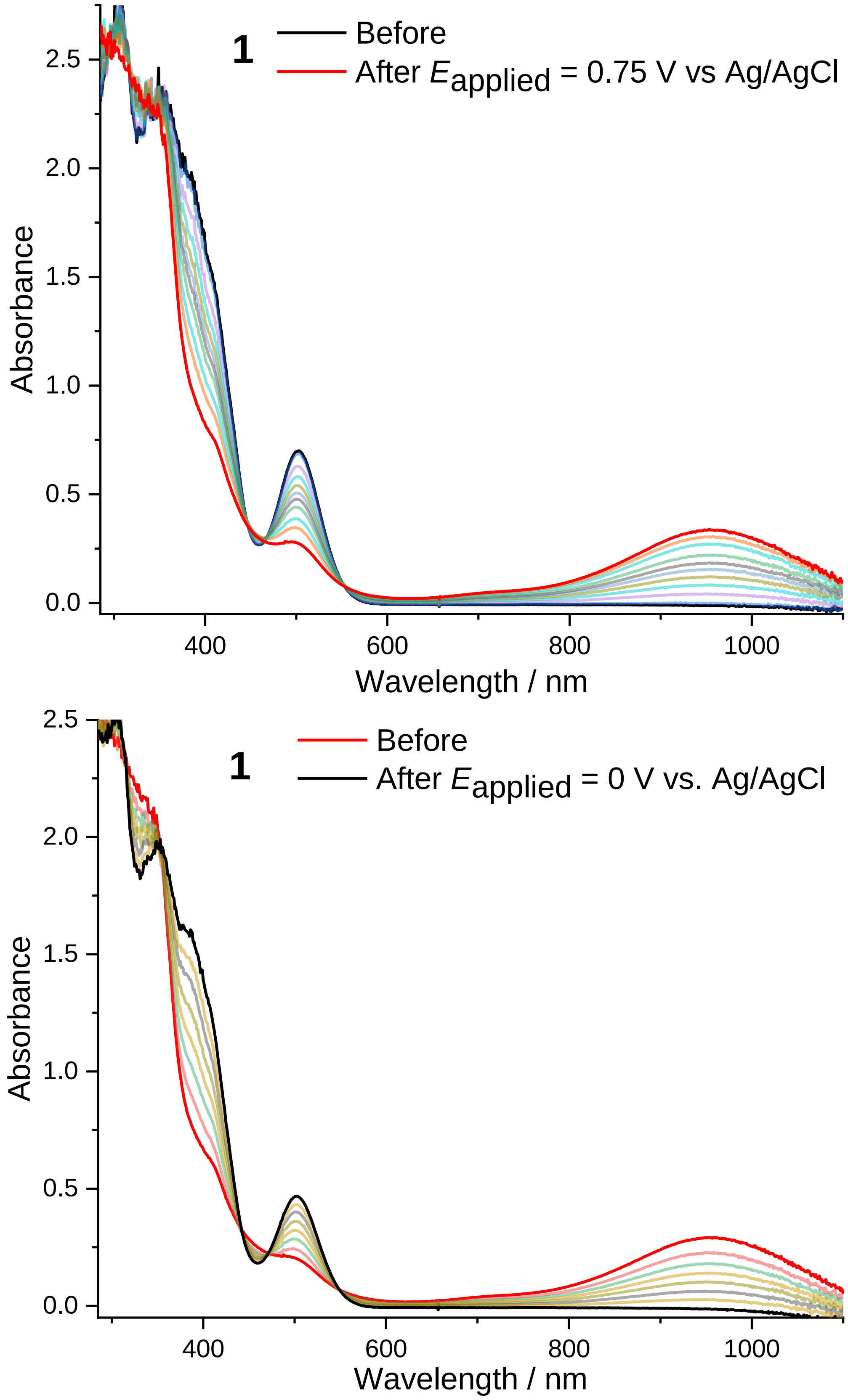

Figure S9. Spectroelectrochemical study of **1** in CH_3CN with 0.1 M [*n*Bu_4N]PF_6 as electrolyte. Top: spectral changes upon oxidation. Bottom: spectral changes associated with the reduction of oxidized species.

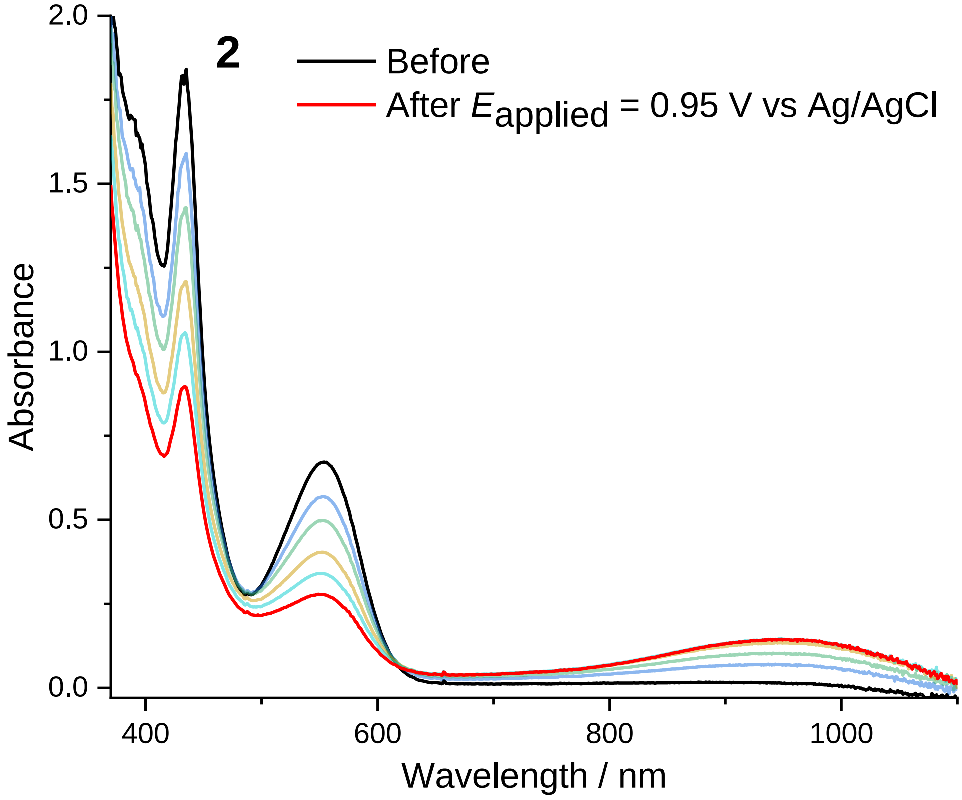

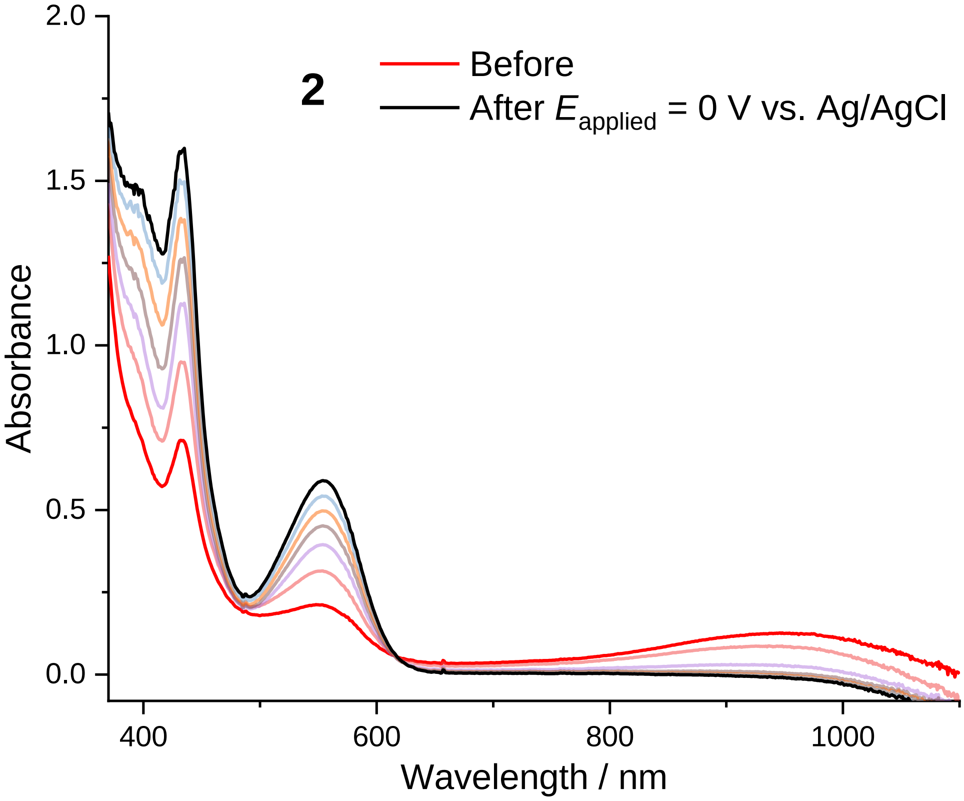

Figure S10. Spectroelectrochemical study of **2** in CH_3CN with 0.1 M [*n*Bu_4N]PF_6 as electrolyte. Top: spectral changes upon oxidation. Bottom: spectral changes associated with the reduction of oxidized species.

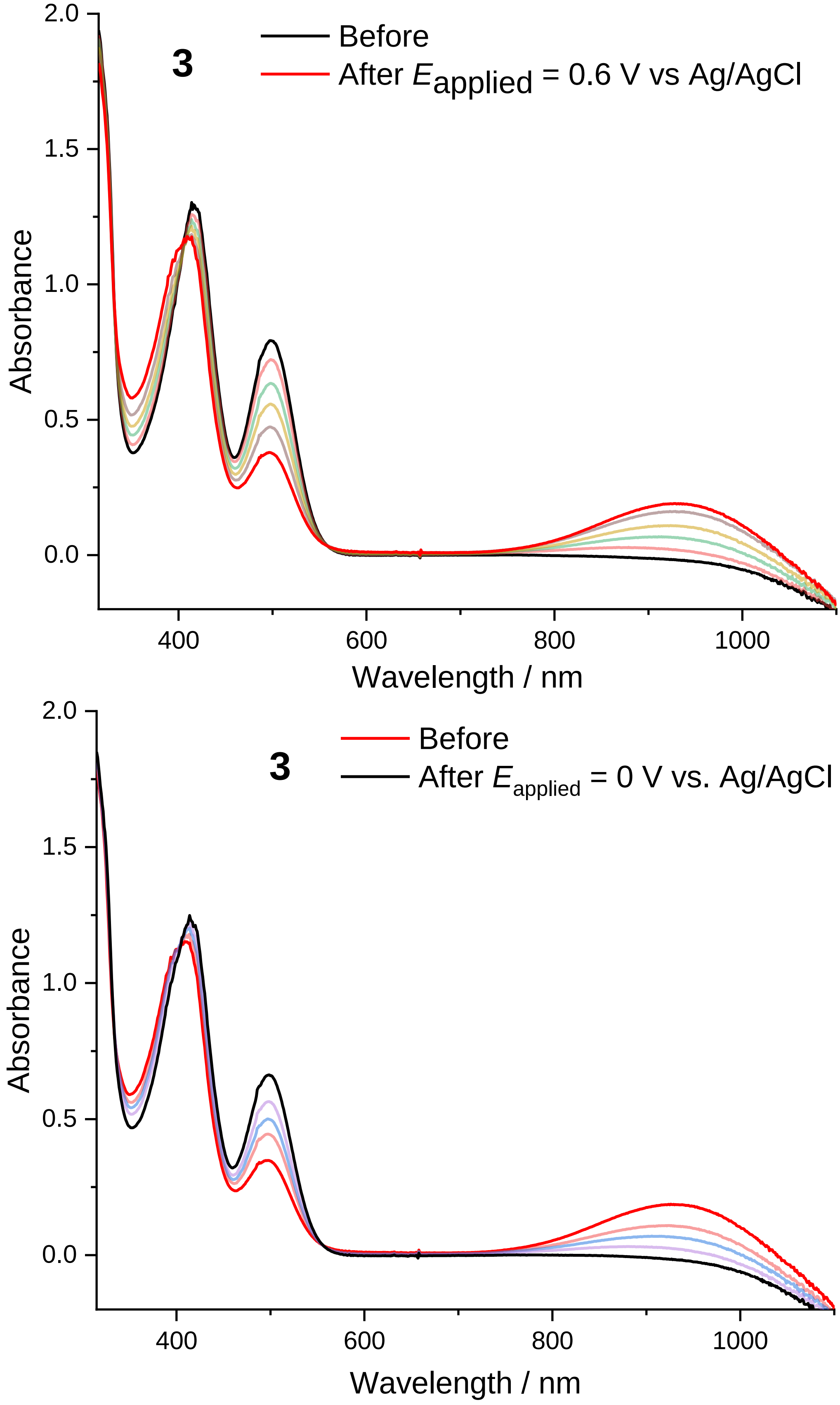

Figure S11. Spectroelectrochemical study of **3** in CH_3CN with 0.1 M [*n*Bu_4N]PF_6 as electrolyte. Top: spectral changes upon oxidation. Bottom: spectral changes associated with the reduction of oxidized species.

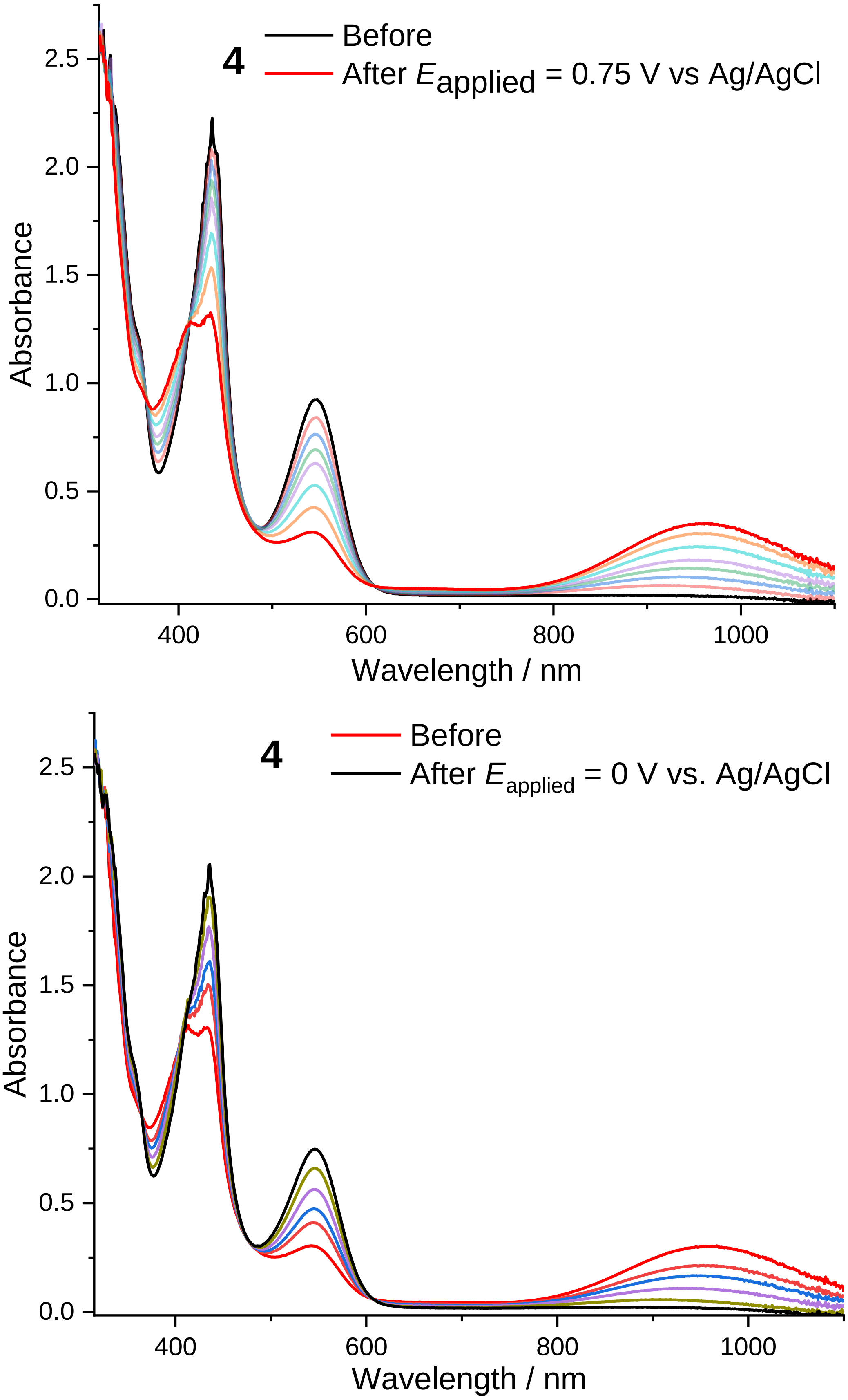

Figure S12. Spectroelectrochemical study of **4** in CH_3CN with 0.1 M [*n*Bu_4N]PF_6 as electrolyte. Top: spectral changes upon oxidation. Bottom: spectral changes associated with the reduction of oxidized species.

EPR spectra

The frozen solution EPR spectrum (at 100 K) of complex **1** upon oxidation, with $AgBF_4$ in DCM at -78 °C, shows an axial signal with $g_{\perp}$ = 2.225, $g_{\parallel}$ = 2.012 (Figure S13, top). The nearly 1:1:1 triplet feature at $g_{\parallel}$ = 2.012 could be fitted assuming super-hyperfine splitting arising from one ^{14}N nucleus with $A_{\parallel}$ = 21 gauss. This fitting parameter indicates that the oxidized complex is best described as a valence trapped species at the EPR time scale at 100 K. A minor signal from an unidentified organic radical slightly distorts the line shape at g = 2.0043. Another axial signal at $g_{\perp}$ = 2.342, $g_{\parallel}$ = 2.008, with ~ 4% concentration of $\mathbf{1}^{+}$, attributed to species **II**, is also discernible. Upon standing at room temperature for four hours, this signal grew in intensity along with another one at $g_{\perp}$ = 2.229, $g_{\parallel}$ = 2.012, attributed to species **III**. In parallel, the signal of $\mathbf{1}^{+}$ dropped markedly in intensity (Figure S13, bottom). Since they did not show appreciable NIR absorptions, we tentatively assigned them as non-coupled Ni(III) species, with or without extra axial ligand coordination.

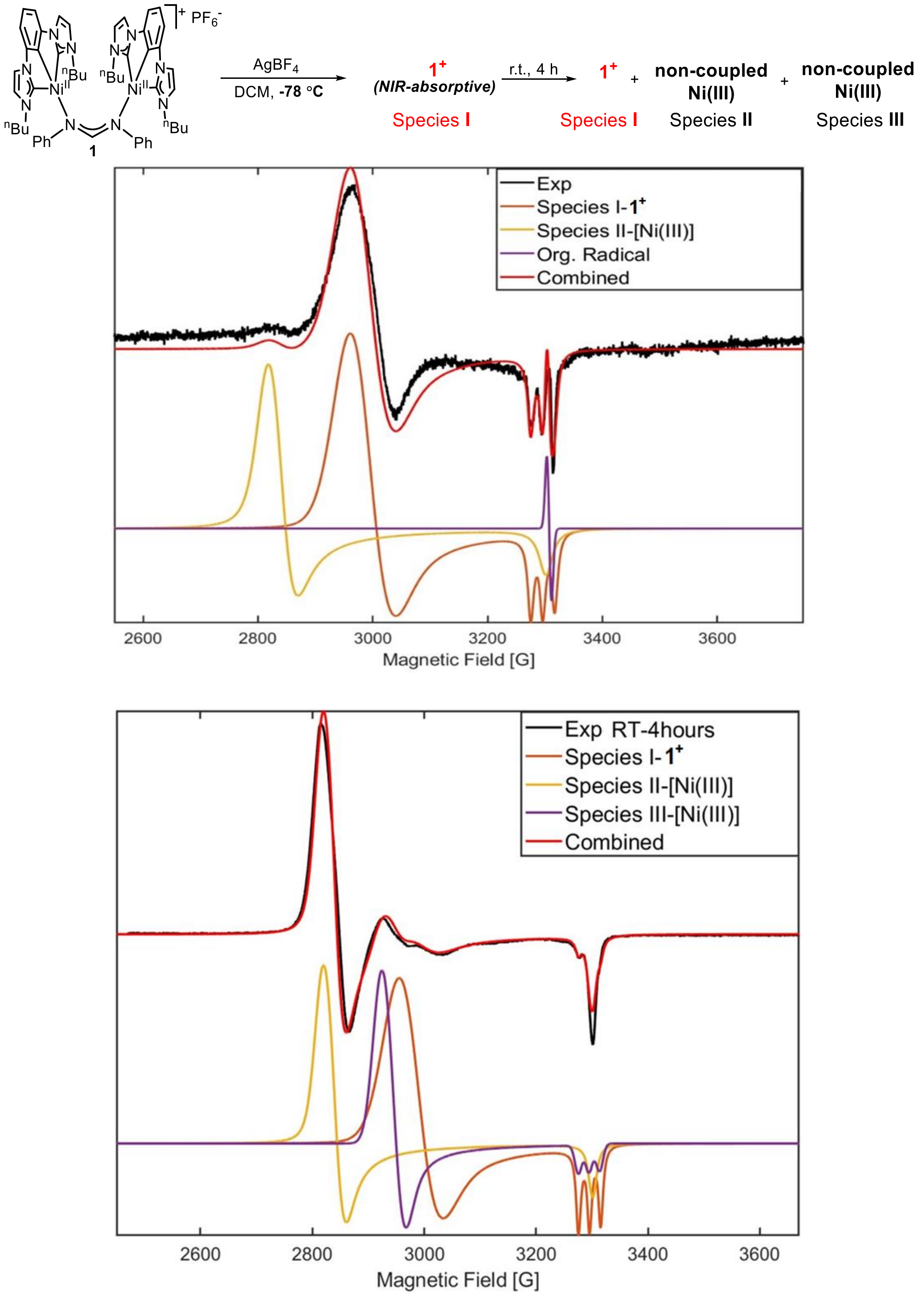

Figure S13. Top: Sample prepared at 195 K, and tested at 100 K. Bottom: the same sample stay at room temperature for 4 hours before tested at 100 K.

Photophysical properties

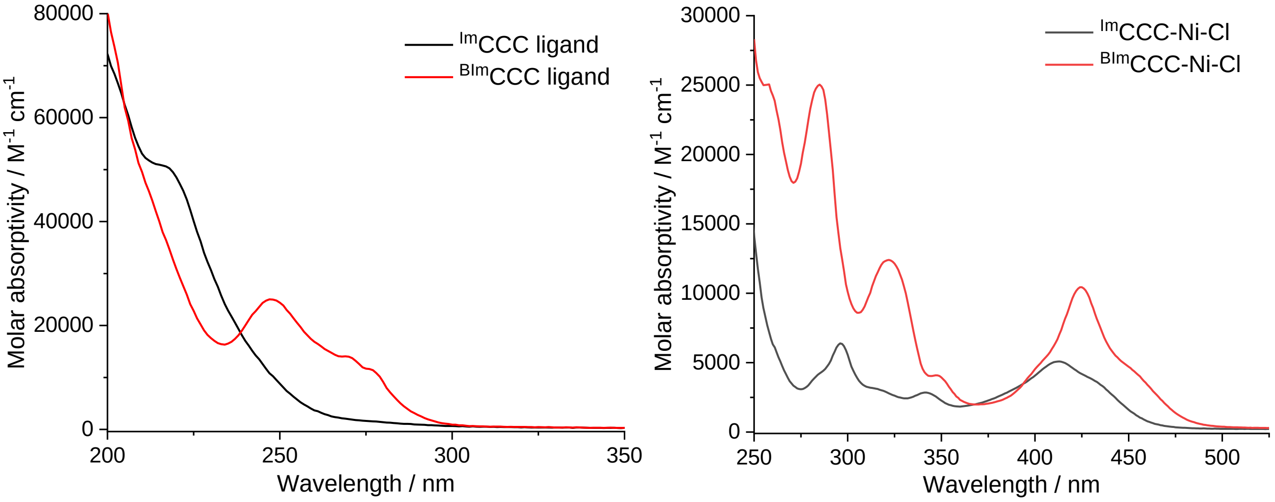

Figure S14. Left: UV-vis absorption spectra of ImCCC and BImCCC ligand in CH_3CN. Right: UV-vis absorption spectra of ImCCC-Ni-Cl and BImCCC-Ni-Cl complexes in DCM.

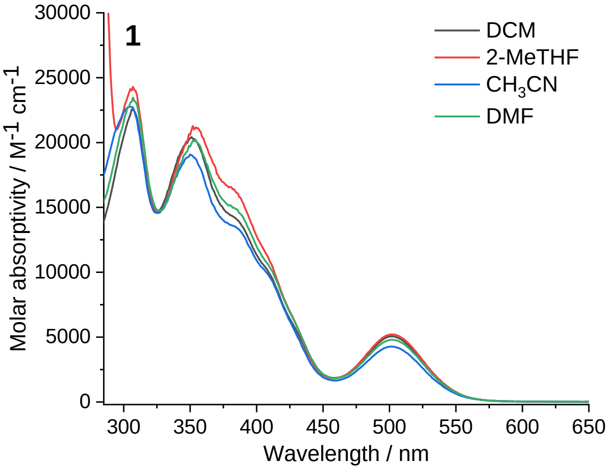

Figure S15. UV-vis absorption spectra of **1** (8×10^{-5} M) in different solvents at room temperature.

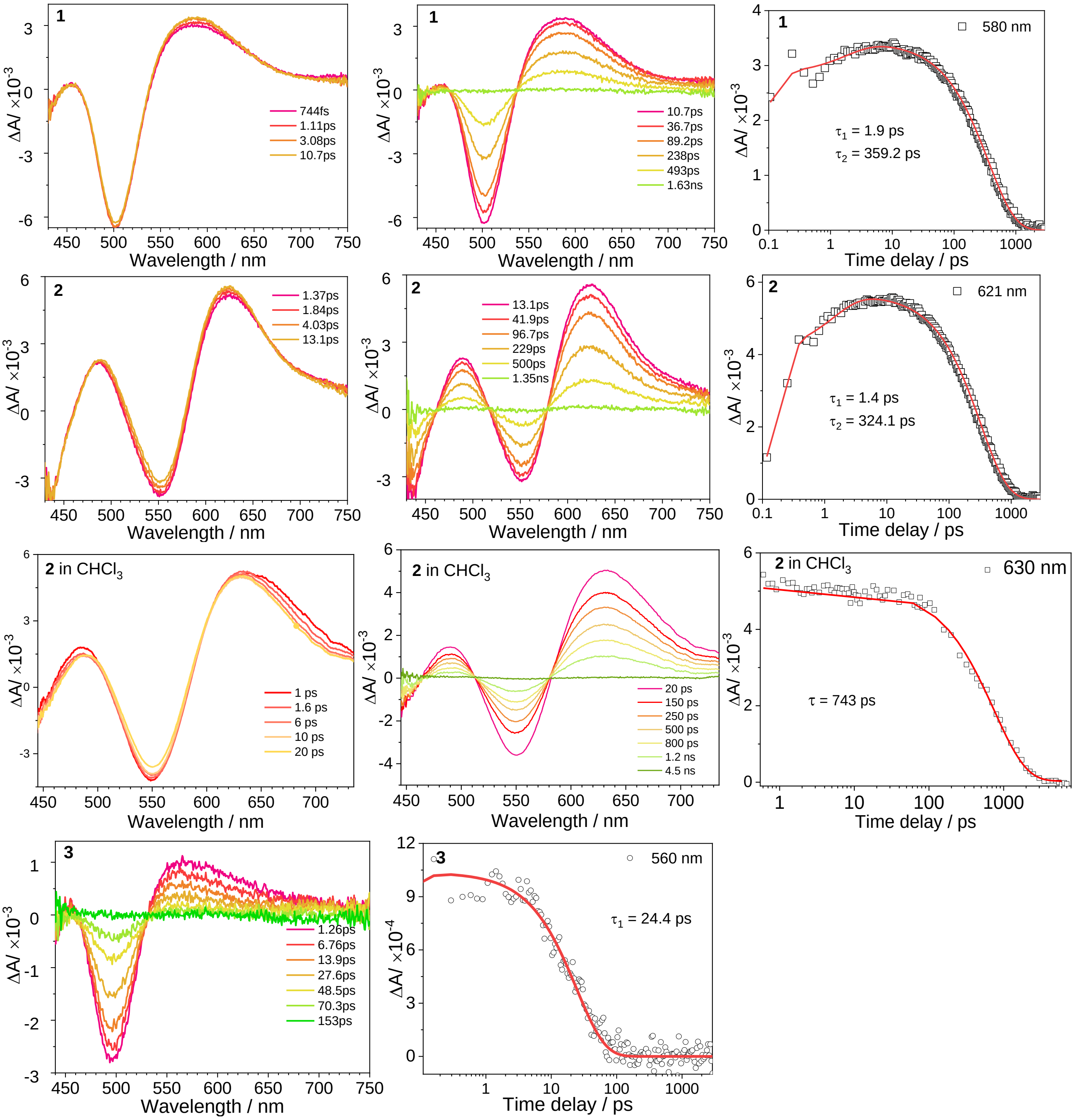
1
744fs
1.11ps
3.08ps
10.7ps
ΔA/ ×10-3
Wavelength / nm
1
10.7ps
36.7ps
89.2ps
238ps
493ps
1.63ns
1
580 nm
τ1 = 1.9 ps
τ2 = 359.2 ps
Time delay / ps
2
1.37ps
1.84ps
4.03ps
13.1ps
2
13.1ps
41.9ps
96.7ps
229ps
500ps
1.35ns
2
621 nm
τ1 = 1.4 ps
τ2 = 324.1 ps
2 in CHCl3
1 ps
1.6 ps
6 ps
10 ps
20 ps
2 in CHCl3
20 ps
150 ps
250 ps
500 ps
800 ps
1.2 ns
4.5 ns
2 in CHCl3
630 nm
τ = 743 ps
3
1.26ps
6.76ps
13.9ps
27.6ps
48.5ps
70.3ps
153ps
3
560 nm
τ1 = 24.4 ps
ΔA/ ×10-4

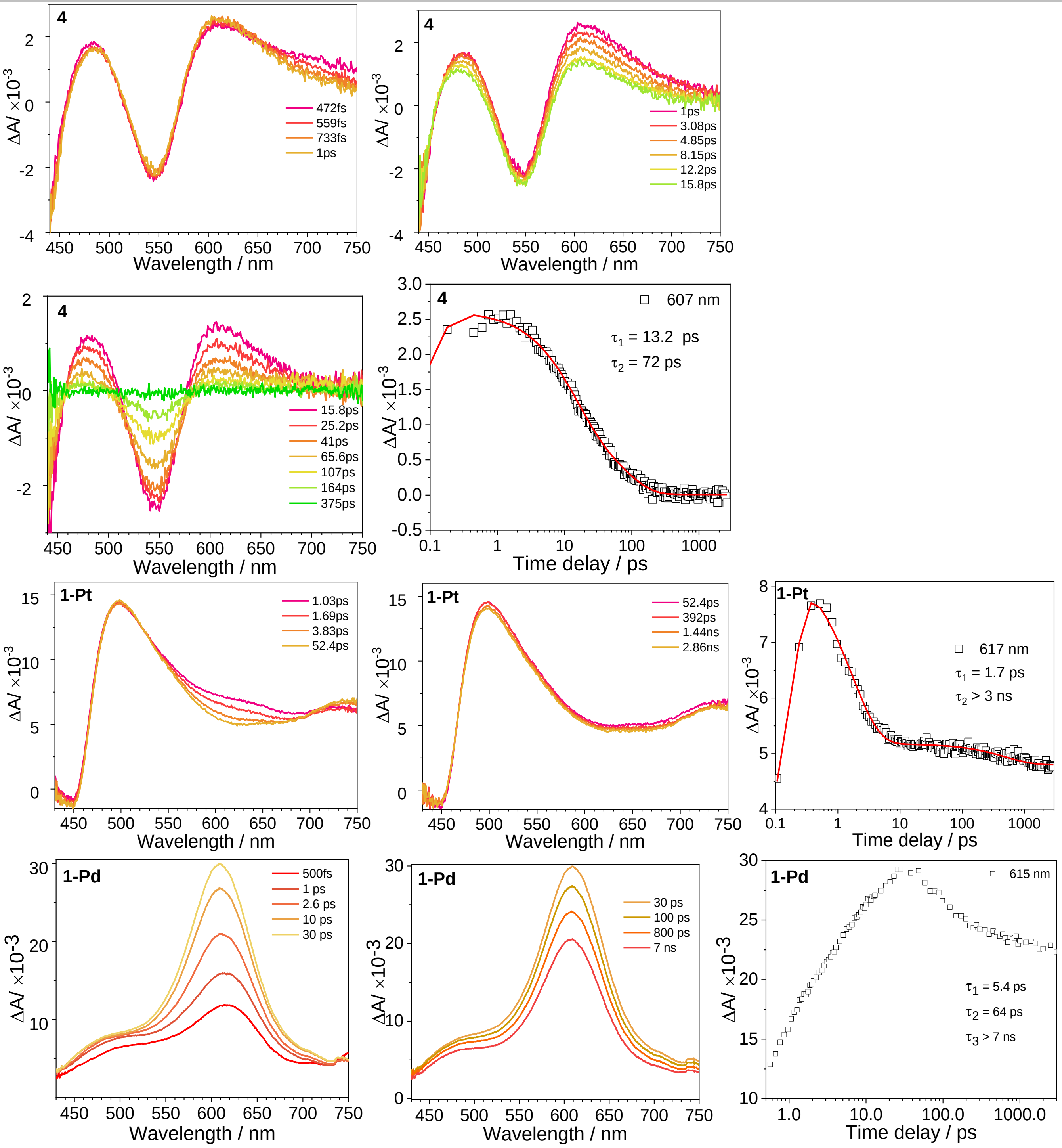

Figure S16. fs-TA spectra and the kinetics of ΔO.D. of all dinuclear complexes in CH_3CN, and those of **2** in $CHCl_3$ ($\lambda_{excitation}$ = 400 nm) at room temperature.

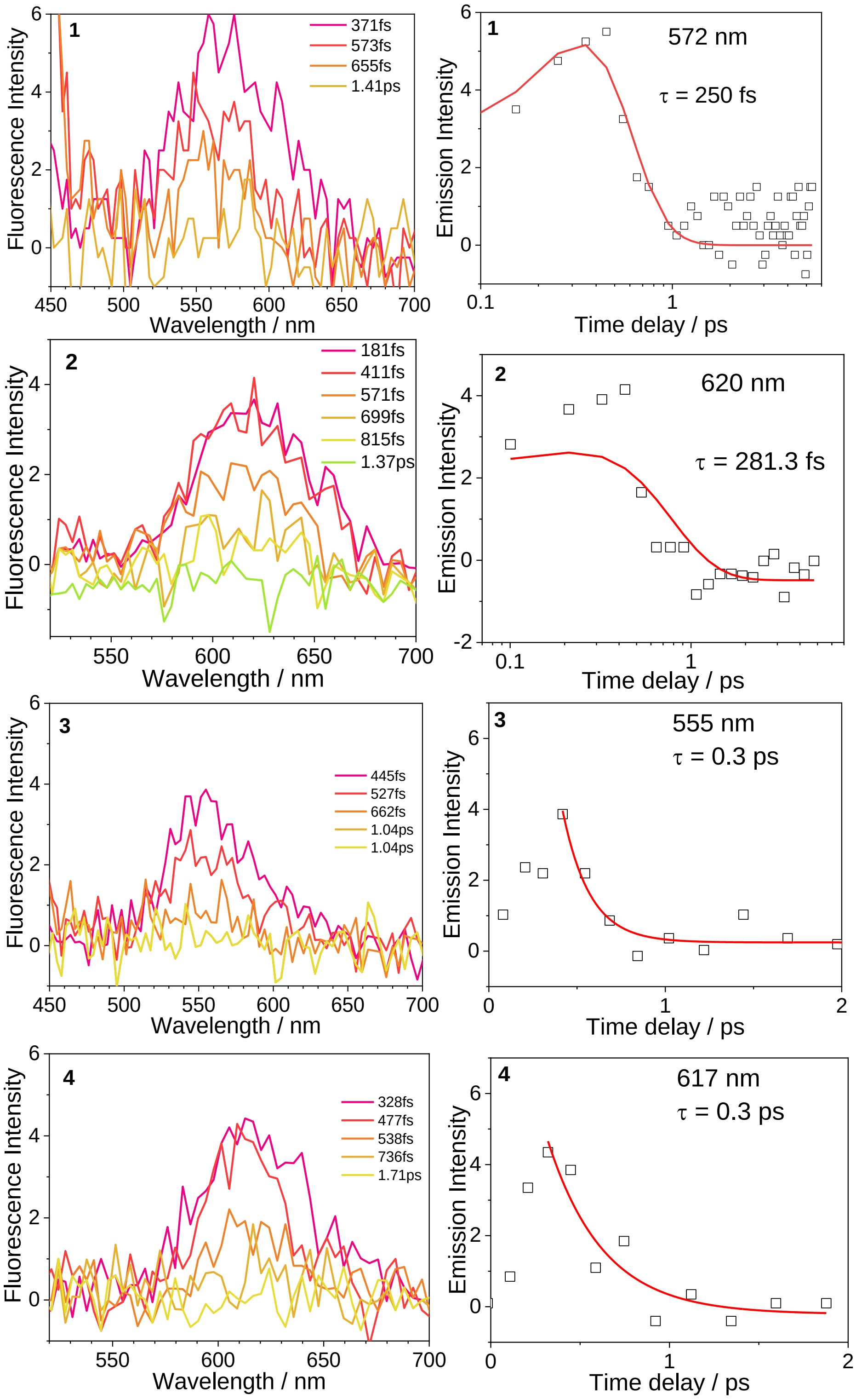

Figure S17. fs-TRF spectra of dinuclear Ni^{II} complexes in CH_3CN ($\lambda_{excitation}$ = 400 nm).

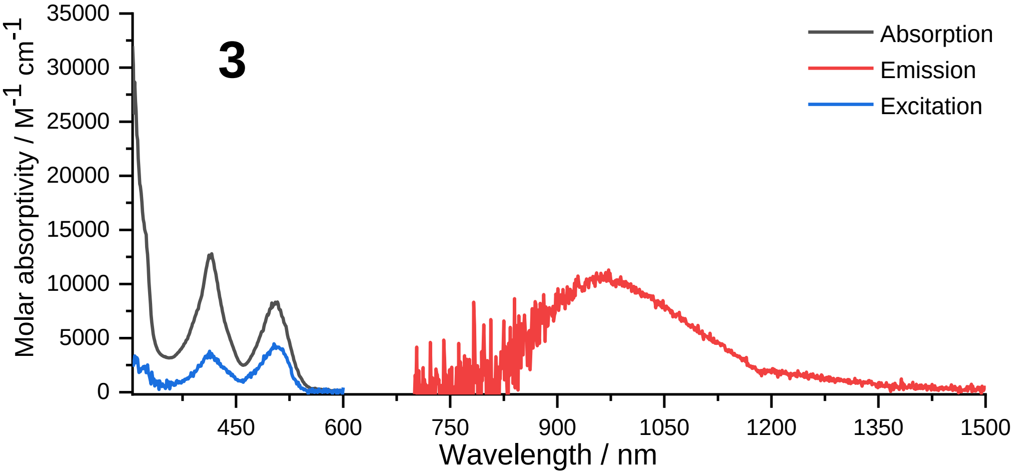

Figure S18. UV-vis absorption spectra of **3** (10^{-4} M) in 2-MeTHF at 173 K, emission and excitation spectra of **3** (10^{-4} M) in 2-MeTHF at 77 K.

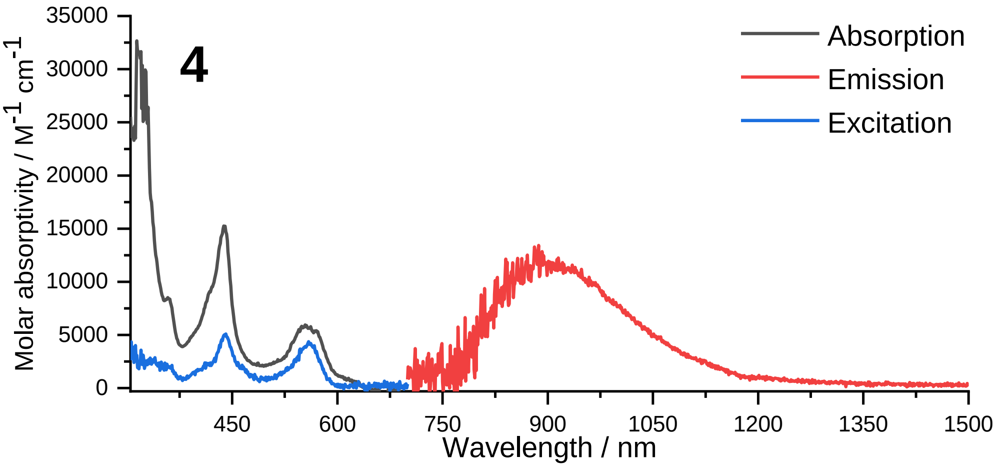

Figure S19. UV-vis absorption spectra of **4** (10^{-4} M) in 2-MeTHF at 173 K, emission and excitation spectra of **4** (10^{-4} M) in 2-MeTHF at 77 K.

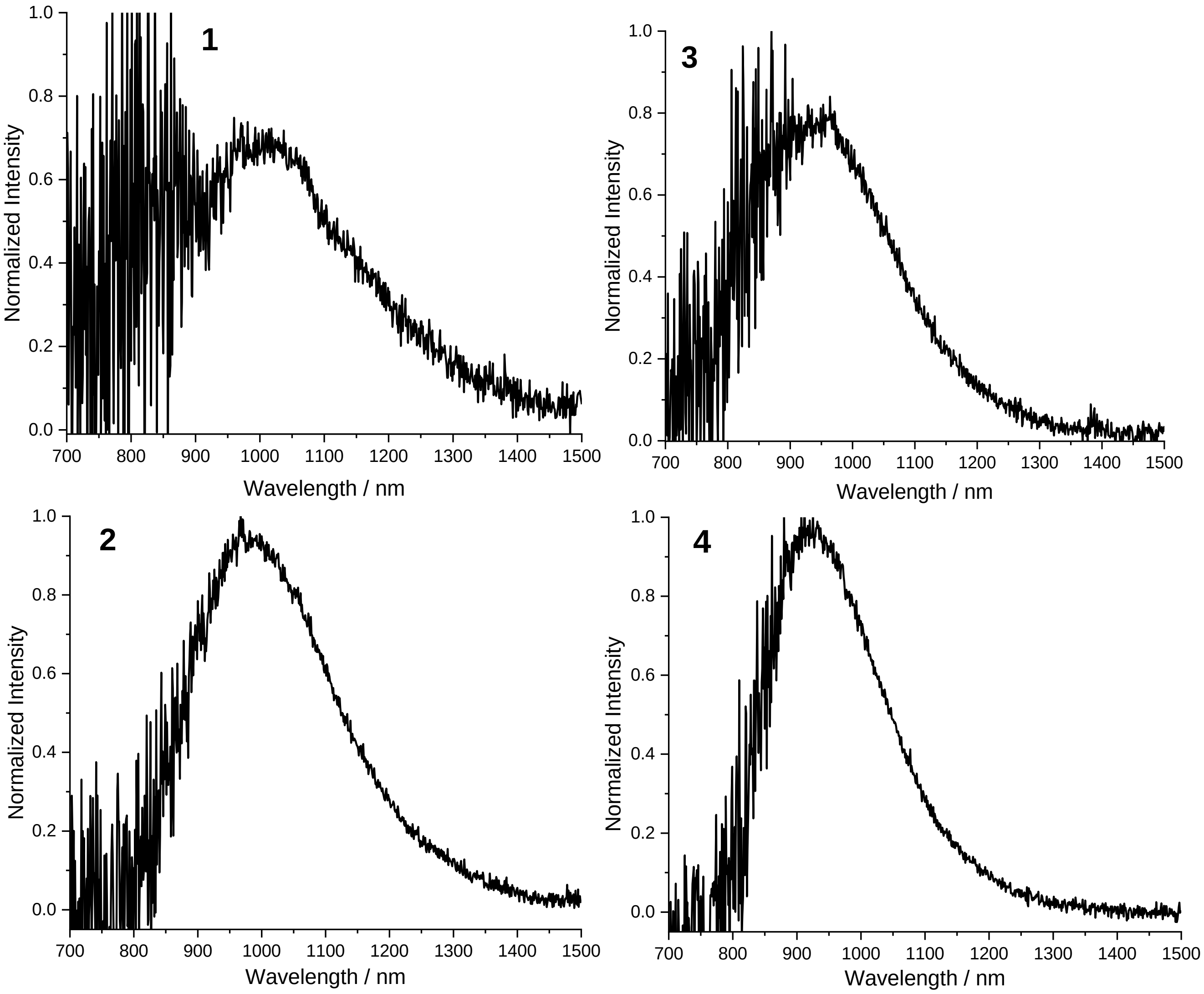

Figure S20. Emission spectra of dinuclear Ni^{II} complexes in the solid state at 77 K.

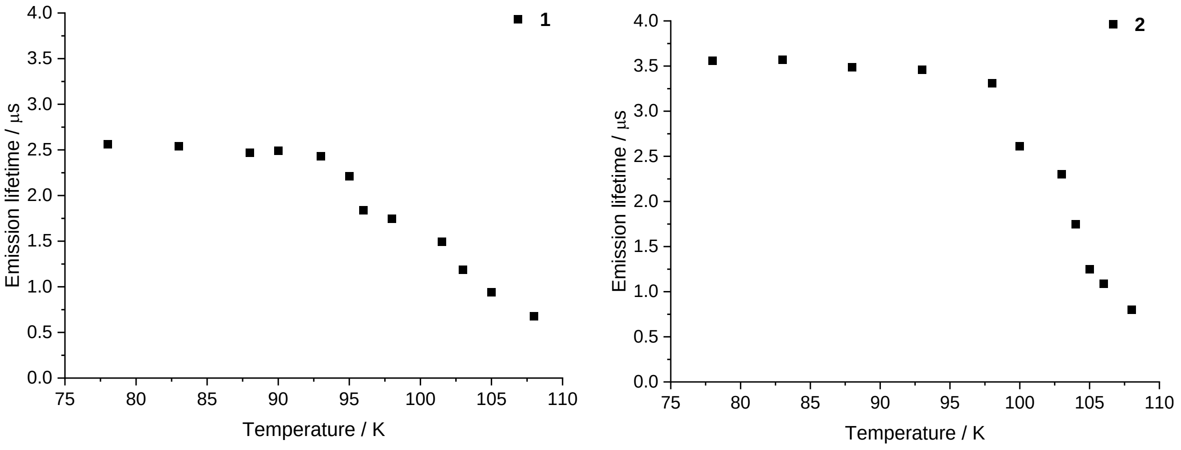

Figure S21. Temperature dependence of the emission lifetime of **1** (1×10^{-4} M) and **2** (1×10^{-4} M) in 2-MeTHF.

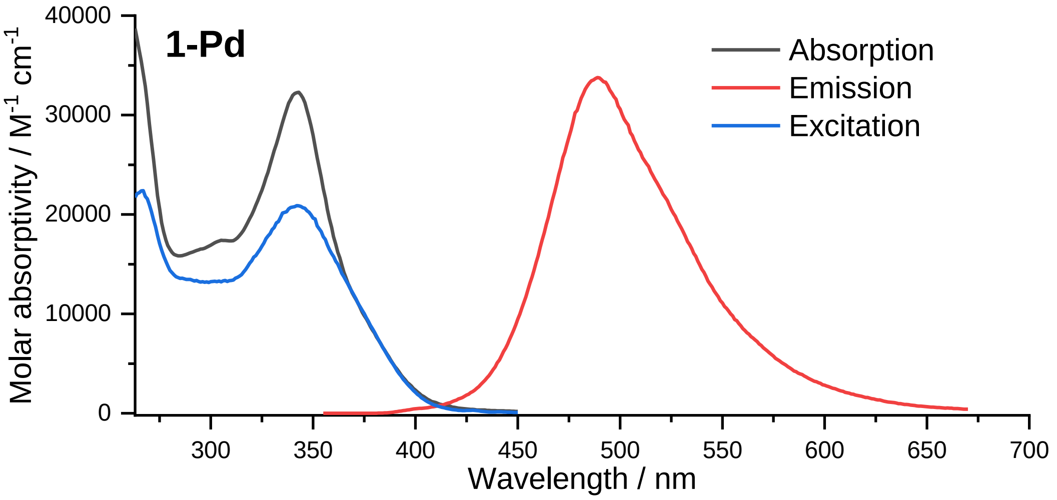

Figure S22. UV-vis absorption spectra, emission and excitation spectra of **1-Pd** (2 x 10^{-5} M) in CH_3CN at room temperature.

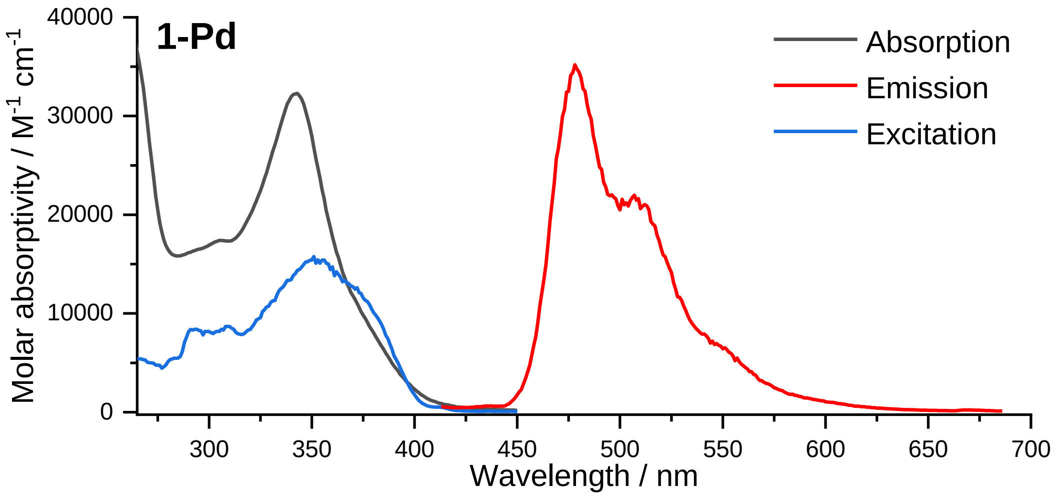

Figure S23. UV-vis absorption spectra of **1-Pd** in CH_3CN (2 x 10^{-5} M, room temperature), emission and excitation spectra of **1-Pd** in 2-MeTHF at 77 K.

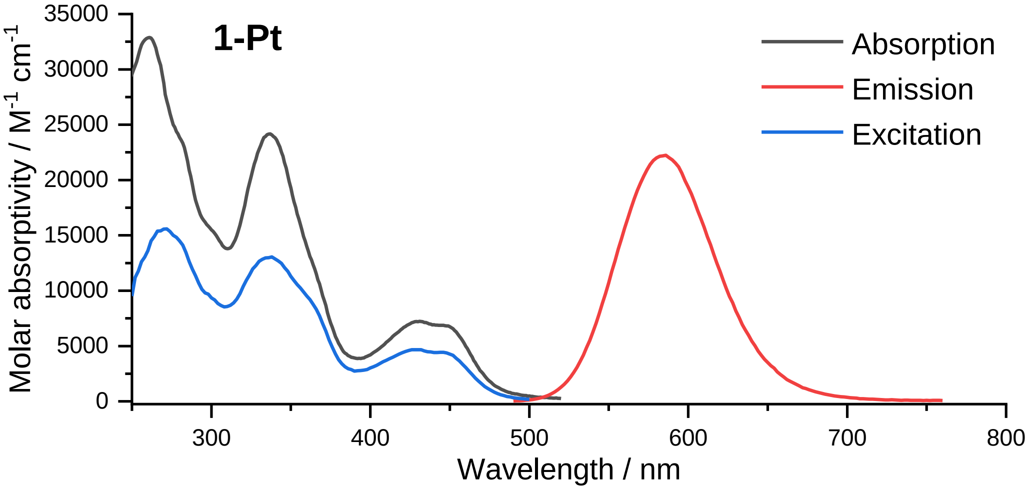

Figure S24. UV-vis absorption spectra, emission and excitation spectra of **1-Pt** (2×10^{-5} M) in CH_3CN at room temperature.

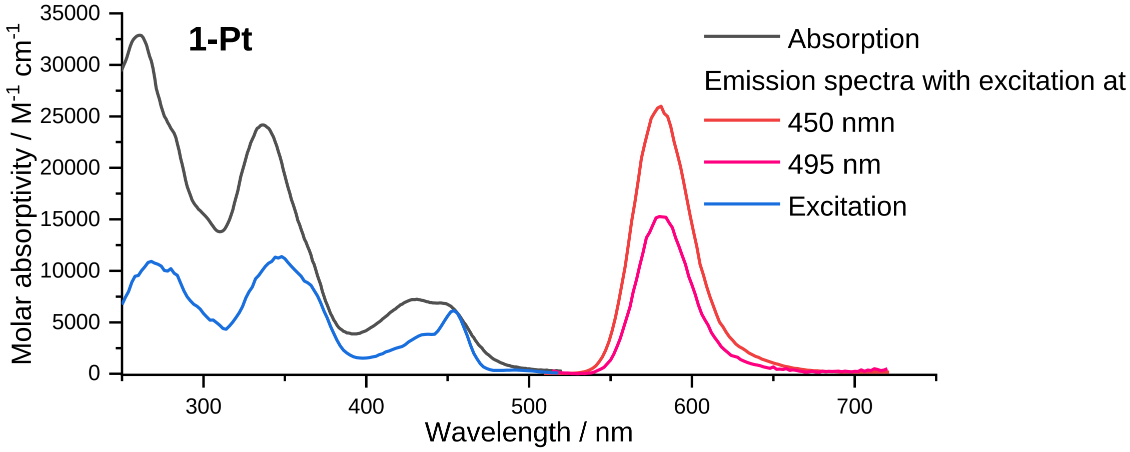

Figure S25. UV-vis absorption spectra of **1-Pt** in CH_3CN (2×10^{-5} M, room temperature), emission and excitation spectra of **1-Pt** in 2-MeTHF at 77 K.

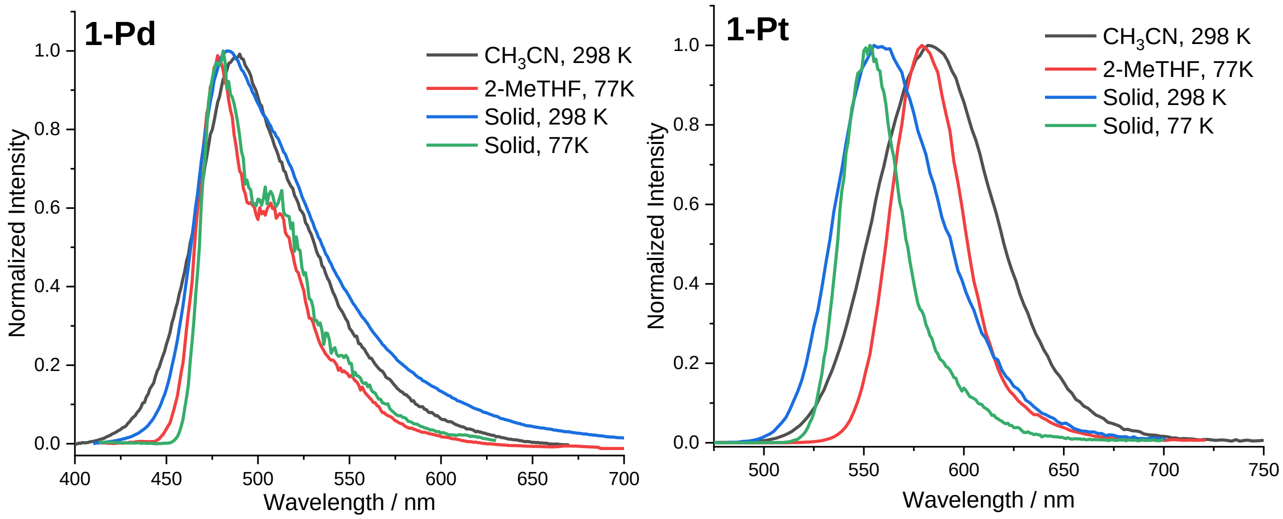

Figure S26. Left, emission spectra of **1-Pd** in CH_3CN (2 x 10^{-5} M, 298 K), 2-MeTHF(77 K), solid state (298 K, 77 K). Right, emission spectra of **1-Pt** in CH_3CN (2 x 10^{-5} M, 298 K), 2-MeTHF(77 K), solid state (298 K, 77 K).

DFT calculations

Ground state geometry optimization

We have used a number of density functionals (DF = M06,[17] M06L,[1] MN15,[18] PBE0-GD3BJ,[19-20] TPSSh,[21-22] and TPSSh-GD3BJ[20-22]) for the geometry optimization of the S_0 state of **1** in acetonitrile solution using the conductor-like polarizable continuum model (CPCM).[23-24] The functional that best matches with the X-ray structure of **1** is M06L. Therefore, we have chosen M06L for geometry optimization of the ground and excited states for both **1** and **2**.

Table S9. The Ni-Ni distance of **1** at the DFT-optimized ground state geometry using different functionals.

DF	M06	M06L	MN15	PBE0-GD3BJ	TPSSh	TPSSh-GD3BJ	Exp.
$r_{Ni1\text{-}Ni2}$ / Å	3.01567	2.90540	2.84866	2.88384	3.07132	2.78133	2.94681

The absorption energies from TDA-M06L calculations were underestimated. Thus, we have used the TPSSh functional,[21-22] together with the D3 version of Grimme's dispersion with Becke-Johnson damping (GD3BJ) [20] to simulate the absorption spectra of **1** and **2** and the results agree nicely with the experimental UV-Vis spectra in 2-MeTHF solution.

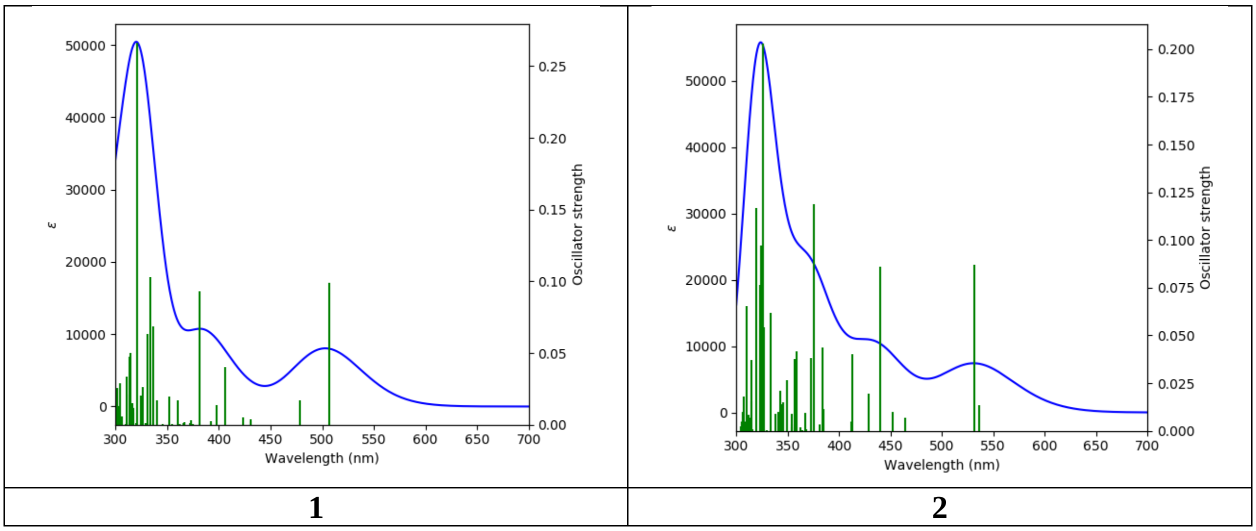

1	2

Figure S27. Simulated absorption spectra of **1** and **2** in THF solution from TDA-TPSSh-GD3BJ calculations at their respective M06L-optimized ground state geometries.

Table S10. Calculated low-lying singlet and triplet excited state energies (λ / nm), the associated oscillator strengths (*f*) at the M06L-optimized *ground* state (S_0) geometry of **1** in THF solution by TDA-TPSSh-GD3BJ method. The values in the parentheses are the % contributions of that particular configuration state function (CSF).

	Energy (cm^{-1})	λ (nm)	*F*	Major contribs
T_1	16839	594	0	HOMO->LUMO (49%), HOMO->L+5 (34%)
T_2	17024	587	0	H-6->L+5 (18%), HOMO->L+1 (22%), HOMO->L+6 (44%)
T_3	17555	570	0	HOMO->LUMO (48%), HOMO->L+5 (36%)
**S_1**	**19741**	**507**	**0.099**	**HOMO->LUMO (96%)**
**S_2**	**20895**	**479**	**0.0169**	**H-1->LUMO (98%)**
**S_3**	**23188**	**431**	**0.0022**	**H-2->LUMO (86%)**
**S_4**	**23193**	**431**	**0.004**	**H-3->LUMO (91%)**
**S_5**	**23599**	**424**	**0.0051**	**H-5->LUMO (16%), HOMO->L+1 (64%)**
**S_6**	**24630**	**406**	**0.0403**	**H-4->LUMO (95%)**
**S_7**	**25138**	**398**	**0.0136**	**H-5->LUMO (67%), HOMO->L+1 (20%)**
**S_8**	**25486**	**392**	**0.0023**	**HOMO->L+5 (77%)**
**S_9**	**26011**	**384**	**0.0001**	**H-6->LUMO (23%), HOMO->L+3 (27%), HOMO->L+6 (14%)**
**S_{10}**	**26210**	**382**	**0.0933**	**H-1->L+1 (45%), H-1->L+6 (23%)**

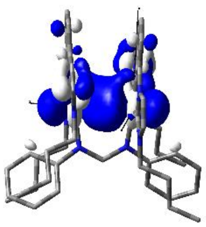<br>MO246 (H-6) -5.83 eV<br>Ni1 (42%) CCC1 (7%)<br>Ni2 (42%) CCC2 (7%)<br>NN (2%)	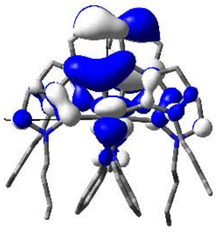<br>MO247(H-5) -5.54 eV<br>Ni1 (25%) CCC1 (24%)<br>Ni2 (25%) CCC2 (23%)<br>NN (2%)	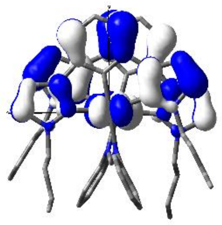<br>MO248 (H-4) -5.53 eV<br>Ni1 (27%) CCC1 (23%)<br>Ni2 (27%) CCC2 (23%)
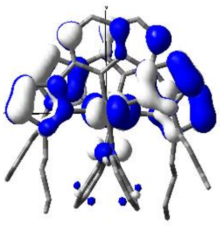<br>MO249 (H-3) -5.38 eV<br>Ni1 (30%) CCC1 (17%)<br>Ni2 (30%) CCC2 (17%)<br>NN (4%)	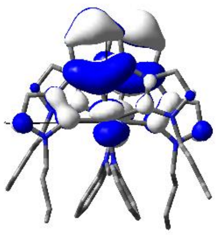<br>MO250 (H-2) -5.35 eV<br>Ni1 (26%) CCC1 (24%)<br>Ni2 (26%) CCC2 (24%)<br>NN (4%)	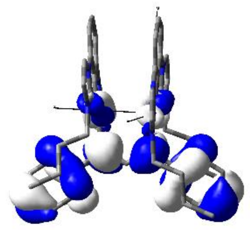<br>MO251 (H-1) -5.02 eV<br>Ni1 (10%) CCC1 (3%)<br>Ni2 (10%) CCC2 (3%)<br>NN (74%)
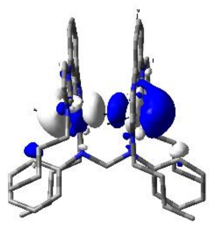<br>MO252 (HOMO) -4.88 eV<br>Ni1 (45%) CCC1 (4%)<br>Ni2 (45%) CCC2 (4%)<br>NN (1%)	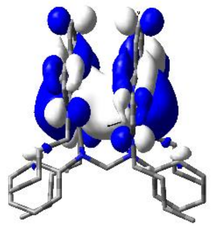<br>MO253 (LUMO) -2.07 eV<br>Ni1 (12%) CCC1 (37%)<br>Ni2 (12%) CCC2 (37%)<br>NN (2%)	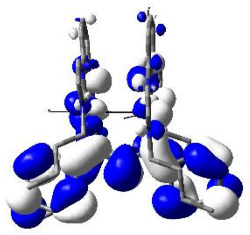<br>MO254 (L+1) -1.45 eV<br>Ni1 (5%) CCC1 (9%)<br>Ni2 (5%) CCC2 (9%)<br>NN (71%)
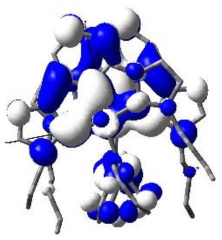<br>MO256 (L+3) -1.15 eV<br>Ni1 (5%) CCC1 (38%)<br>Ni2 (5%) CCC2 (36%)<br>NN (15%)	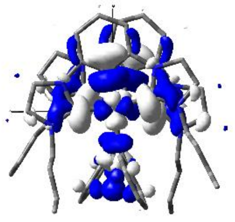<br>MO258 (L+5) -1.02 eV<br>Ni1 (26%) CCC1 (19%)<br>Ni2 (25%) CCC2 (19%)<br>NN (12%)	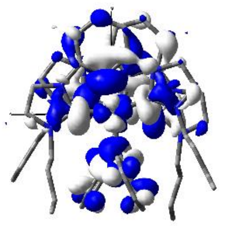<br>MO259 (L+6) -0.91 eV<br>Ni1 (20%) CCC1 (20%)<br>Ni2 (21%) CCC2 (20%)<br>NN (19%)

Figure S28. Frontier molecular orbital (MO) surfaces for **1** obtained from TDA-TPSSh-GD3BJ calculations at the M06L-optimzed ground state geometry (surface isovalue = 0.02 a.u.; CCC1 and CCC2 = carbene-phenyl-carbene pincer ligand coordinated to Ni1 and Ni2 respectively).

Table S11. Cartesian coordinates (Å) of **1** at the M06L-optimized ground state in THF solution.

	X	Y	Z
Ni1	-0.01915	0.107874	-0.00956
Ni2	-0.00832	-0.03277	2.890619
C3	1.899411	0.153976	-0.45009
N4	2.957973	-0.65007	-0.7317
C5	4.099386	0.090624	-0.99994
H6	5.040407	-0.38231	-1.24478
C7	3.763136	1.403048	-0.88278
H8	4.35279	2.300183	-1.00427
N9	2.427682	1.423265	-0.55654
C10	2.909625	-2.10568	-0.82075
H11	1.992576	-2.4169	-0.31548
H12	3.757234	-2.51305	-0.25077
C13	2.918491	-2.61054	-2.25485
H14	3.897089	-2.4161	-2.71868
H15	2.179984	-2.03848	-2.83511
C16	2.579465	-4.09223	-2.32606
H17	3.30331	-4.66735	-1.72871
H18	1.597891	-4.25015	-1.85035
C19	2.547287	-4.61646	-3.75
H20	2.2762	-5.67755	-3.78336
H21	3.5233	-4.50649	-4.23937
H22	1.812289	-4.06777	-4.35278
C23	0.232946	1.948398	-0.16776
C24	1.496532	2.474288	-0.38507
C25	1.727715	3.846784	-0.42831
H26	2.721834	4.258558	-0.594
C27	0.628083	4.695744	-0.25812
H28	0.78343	5.771816	-0.29022
C29	-0.66574	4.203179	-0.05295
H30	-1.50079	4.888877	0.079576
C31	-0.83157	2.822636	-0.00846
C32	-1.87097	0.719391	0.195034
N33	-2.02354	2.089042	0.183045
C34	-3.33412	2.467469	0.353691
H35	-3.6493	3.500166	0.386064
C36	-4.04221	1.312341	0.463335

H37	-5.09938	1.138806	0.610027
N38	-3.14	0.264799	0.36644
C39	-3.55558	-1.13294	0.400722
H40	-4.31825	-1.21934	1.186543
H41	-2.68651	-1.72045	0.715599
C42	-4.11043	-1.63014	-0.92378
H43	-3.30005	-1.67386	-1.66709
H44	-4.84725	-0.90708	-1.30571
C45	-4.76336	-2.99693	-0.77272
H46	-4.0464	-3.69042	-0.307
H47	-5.60709	-2.91773	-0.0697
C48	-5.23965	-3.56727	-2.09581
H49	-5.70748	-4.55005	-1.96844
H50	-4.40066	-3.68462	-2.7938
H51	-5.97612	-2.90774	-2.57229
C52	1.773577	0.778287	2.758532
N53	3.081573	0.473182	2.551859
C54	3.872722	1.610871	2.561143
H55	4.941295	1.55892	2.404852
C56	3.052107	2.672737	2.777195
H57	3.261071	3.730481	2.845367
N58	1.787193	2.148933	2.902836
C59	3.637924	-0.86459	2.384255
H60	4.406791	-0.79385	1.602637
H61	2.834298	-1.50345	2.002718
C62	4.239018	-1.43276	3.659308
H63	3.436265	-1.63393	4.384815
H64	4.896536	-0.6802	4.120666
C65	5.029702	-2.70332	3.380244
H66	4.388111	-3.41736	2.841306
H67	5.860017	-2.4683	2.696373
C68	5.56395	-3.3498	4.644895
H69	6.22844	-2.66802	5.190836
H70	6.131277	-4.26135	4.425449
H71	4.742633	-3.62198	5.320335
C72	-0.44264	1.749681	3.22495
C73	0.528408	2.737208	3.157291
C74	0.225918	4.083269	3.335614

H75	0.988412	4.857851	3.276858
C76	-1.10963	4.422152	3.580541
H77	-1.37137	5.469044	3.716781
C78	-2.11823	3.45523	3.66108
H79	-3.14758	3.748401	3.86092
C80	-1.75141	2.12331	3.485613
C81	-1.92038	-0.22119	3.323531
N82	-2.57215	0.972871	3.549826
C83	-3.89756	0.78817	3.865326
H84	-4.57383	1.60556	4.069363
C85	-4.10114	-0.55622	3.852032
H86	-4.98958	-1.1418	4.043596
N87	-2.89247	-1.15011	3.520451
C88	-2.7015	-2.59537	3.463252
H89	-1.75994	-2.76313	2.935746
H90	-3.50648	-3.0232	2.848477
C91	-2.65862	-3.24249	4.838511
H92	-3.65165	-3.19592	5.310313
H93	-1.98079	-2.662	5.481149
C94	-2.17318	-4.68264	4.761305
H95	-2.83585	-5.26202	4.100463
H96	-1.18061	-4.69288	4.282314
C97	-2.08973	-5.34507	6.12426
H98	-1.41271	-4.79211	6.787959
H99	-1.71536	-6.37222	6.051517
H100	-3.07195	-5.38237	6.612144
N101	-0.26172	-1.86136	0.227623
C102	-0.68958	-2.61621	-0.87593
C103	-1.31459	-3.8708	-0.76057
H104	-1.53427	-4.28045	0.225682
C105	-1.69914	-4.5744	-1.89815
H106	-2.19103	-5.53973	-1.78501
C107	-1.49169	-4.04226	-3.16981
H108	-1.80088	-4.59506	-4.05442
C109	-0.90512	-2.78233	-3.2923
H110	-0.7468	-2.34595	-4.27733
C111	-0.51282	-2.07652	-2.16141
H112	-0.0477	-1.09238	-2.2498

C113	0.116152	-2.51491	1.316899
H114	0.173188	-3.61259	1.263399
N115	0.42935	-1.93524	2.466786
C116	0.935856	-2.74677	3.494308
C117	0.701754	-2.36105	4.825212
H118	0.131233	-1.44785	5.006119
C119	1.168571	-3.13008	5.884266
H120	0.963984	-2.81416	6.906098
C121	1.887921	-4.30088	5.643059
H122	2.255717	-4.90277	6.471402
C123	2.150072	-4.67827	4.326952
H124	2.742754	-5.5693	4.123378
C125	1.691061	-3.90989	3.261164
H126	1.951546	-4.19389	2.241312

Table S12. Cartesian coordinates (Å) of **1** at the TDA-M06L-optimized S_1 excited state in THF solution.

	X	Y	Z
Ni1	-0.11888	0.081558	0.081235
Ni2	0.10203	-0.03786	2.795273
C3	1.803851	0.198853	-0.35724
N4	2.895864	-0.57429	-0.61918
C5	4.017446	0.206429	-0.85252
H6	4.977198	-0.235	-1.08389
C7	3.641399	1.506953	-0.72833
H8	4.208906	2.42128	-0.82136
N9	2.298458	1.48828	-0.43036
C10	2.891712	-2.02399	-0.7683
H11	1.995356	-2.38954	-0.26254
H12	3.761515	-2.42635	-0.22894
C13	2.895199	-2.47212	-2.22171
H14	3.858429	-2.22206	-2.69115
H15	2.127352	-1.90542	-2.76814
C16	2.611595	-3.96176	-2.34713
H17	3.365247	-4.53102	-1.78196
H18	1.643829	-4.17687	-1.86548
C19	2.579812	-4.43189	-3.78987
H20	2.350634	-5.50095	-3.86108
H21	3.543784	-4.26408	-4.28653
H22	1.815607	-3.89047	-4.36209
C23	0.082837	1.940503	-0.05094
C24	1.334873	2.501959	-0.26687
C25	1.513482	3.881343	-0.33308
H26	2.495094	4.323878	-0.49666
C27	0.382145	4.694401	-0.19961
H28	0.499809	5.774417	-0.25061
C29	-0.89913	4.159105	-0.00863
H30	-1.76134	4.816608	0.088356
C31	-1.02264	2.777344	0.060997
C32	-2.00064	0.642546	0.217781
N33	-2.1911	2.011558	0.221054
C34	-3.52069	2.347656	0.34152
H35	-3.86678	3.370554	0.373016

C36	-4.19708	1.172146	0.414775
H37	-5.2535	0.969654	0.525225
N38	-3.26696	0.147027	0.343991
C39	-3.64905	-1.25808	0.359955
H40	-4.42068	-1.37118	1.134938
H41	-2.77203	-1.82917	0.683973
C42	-4.17708	-1.76585	-0.9725
H43	-3.35621	-1.80341	-1.70463
H44	-4.91688	-1.0528	-1.36726
C45	-4.81713	-3.1393	-0.8259
H46	-4.09809	-3.82473	-0.35108
H47	-5.66847	-3.06768	-0.13132
C48	-5.27609	-3.71644	-2.1521
H49	-5.7317	-4.70538	-2.02806
H50	-4.43109	-3.82202	-2.84467
H51	-6.01793	-3.06668	-2.63363
C52	1.91725	0.721389	2.719159
N53	3.22631	0.3696	2.555823
C54	4.049067	1.484231	2.592418
H55	5.120243	1.398264	2.473221
C56	3.258657	2.573766	2.772892
H57	3.500876	3.624326	2.842169
N58	1.969487	2.096412	2.849751
C59	3.749814	-0.98205	2.417869
H60	4.531004	-0.94533	1.645078
H61	2.937162	-1.60897	2.034947
C62	4.323051	-1.54805	3.707633
H63	3.506884	-1.73836	4.421206
H64	4.978351	-0.79709	4.174916
C65	5.109728	-2.82566	3.450777
H66	4.472859	-3.53956	2.9059
H67	5.95235	-2.6004	2.778794
C68	5.620697	-3.46553	4.728277
H69	6.282357	-2.7838	5.277711
H70	6.184306	-4.38314	4.525079
H71	4.788064	-3.72521	5.394739
C72	-0.28281	1.769431	3.112058

C73	0.731058	2.721261	3.079366
C74	0.46855	4.071042	3.275088
H75	1.258909	4.818819	3.241668
C76	-0.85931	4.453914	3.509273
H77	-1.08558	5.507229	3.658283
C78	-1.90162	3.521643	3.562125
H79	-2.92253	3.845546	3.759364
C80	-1.58444	2.179391	3.370589
C81	-1.82209	-0.15707	3.231301
N82	-2.44113	1.063633	3.430153
C83	-3.77658	0.920284	3.727742
H84	-4.43011	1.760653	3.910954
C85	-4.02129	-0.41684	3.723389
H86	-4.93055	-0.97183	3.908729
N87	-2.83022	-1.05636	3.416179
C88	-2.68807	-2.5069	3.412362
H89	-1.75698	-2.73015	2.886897
H90	-3.51021	-2.92912	2.816336
C91	-2.66496	-3.10641	4.809874
H92	-3.6527	-2.99748	5.282424
H93	-1.95981	-2.53397	5.429759
C94	-2.24683	-4.56917	4.784188
H95	-2.94033	-5.14135	4.1493
H96	-1.25938	-4.64495	4.300584
C97	-2.18464	-5.18362	6.170675
H98	-1.47778	-4.63915	6.809801
H99	-1.86008	-6.22942	6.134637
H100	-3.16392	-5.15582	6.665002
N101	-0.29441	-1.89878	0.243032
C102	-0.72332	-2.63584	-0.8727
C103	-1.33677	-3.8966	-0.77378
H104	-1.54109	-4.3269	0.206936
C105	-1.72934	-4.57927	-1.92123
H106	-2.21458	-5.54949	-1.82268
C107	-1.53857	-4.02028	-3.184
H108	-1.85479	-4.55721	-4.07581
C109	-0.95843	-2.75588	-3.28885

H110	-0.8098	-2.30069	-4.26666
C111	-0.55949	-2.07027	-2.14774
H112	-0.09686	-1.0838	-2.22499
C113	0.121729	-2.55779	1.313407
H114	0.178638	-3.65485	1.259254
N115	0.472074	-1.96896	2.446382
C116	0.975369	-2.76897	3.485752
C117	0.737635	-2.37075	4.811258
H118	0.157574	-1.46288	4.987566
C119	1.207649	-3.12676	5.878491
H120	0.999902	-2.8024	6.896864
C121	1.932287	-4.29633	5.64819
H122	2.304358	-4.8874	6.482325
C123	2.192175	-4.6888	4.336089
H124	2.785966	-5.58111	4.141936
C125	1.729184	-3.93396	3.262449
H126	1.984373	-4.22957	2.244579

Table S13. Calculated low-lying singlet and triplet excited state energies (λ / nm), the associated oscillator strengths (*f*) at the M06L-optimized *ground* state (S_0) geometry of **2** in THF solution by TDA-TPSSh-GD3BJ method. The values in the parentheses are the % contributions of that particular configuration state function (CSF).

	Energy (cm^{-1})	λ(nm)	*f*	Major contribs
T_1	16725	598	0	H-1->LUMO (93%)
T_2	17400	575	0	H-6->L+6 (17%), H-1->L+1 (14%), H-1->L+2 (21%), H-1->L+7 (32%)
T_3	18101	552	0	HOMO->LUMO (87%)
T_4	18107	552	0	H-6->L+7 (14%), H-1->L+6 (47%)
**S_1**	**18639**	**537**	**0.0136**	**HOMO->LUMO (99%)**
**S_2**	**18793**	**532**	**0.0872**	**H-1->LUMO (96%)**
**S_3**	**21083**	**474**	**0**	**H-2->LUMO (91%)**
**S_4**	**21533**	**464**	**0.0069**	**H-3->LUMO (94%)**
**S_5**	**22131**	**452**	**0.01**	**H-4->LUMO (91%)**
**S_6**	**22730**	**440**	**0.0859**	**H-5->LUMO (96%)**
**S_7**	**23338**	**428**	**0.0194**	**H-6->LUMO (54%), H-1->L+1 (34%)**
**S_8**	**24218**	**413**	**0.0401**	**HOMO->L+1 (88%)**
**S_9**	**24253**	**412**	**0.0048**	**H-6->LUMO (31%), H-1->L+1 (42%), H-1->L+2 (10%)**
**S_{10}**	**25450**	**393**	**0.0001**	**H-1->L+1 (13%), H-1->L+2 (58%)**

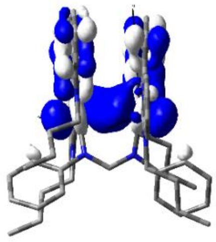

MO298 (H-6) -5.83 eV
Ni1 (36%) CCC1 (13%)
Ni2 (36%) CCC2 (13%)
NN (2%)

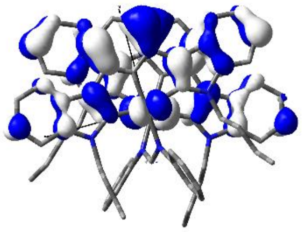

MO299(H-5) -5.70 eV
Ni1 (21%) CCC1 (26%)
Ni2 (24%) CCC2 (28%)

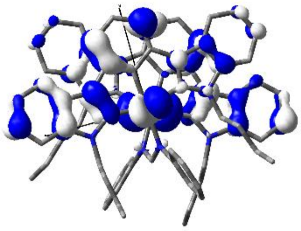

MO300 (H-4) -5.68 eV
Ni1 (33%) CCC1 (19%)
Ni2 (31%) CCC2 (16%)
NN (1%)

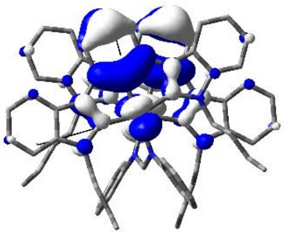

MO301 (H-3) -5.56 eV
Ni1 (25%) CCC1 (25%)
Ni2 (26%) CCC2 (25%)

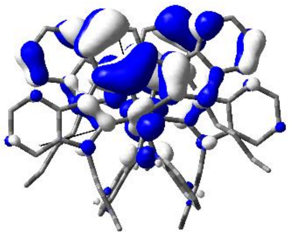

MO302 (H-2) -5.46 eV
Ni1 (12%) CCC1 (35%)
Ni2 (12%) CCC2 (35%)
NN (4%)

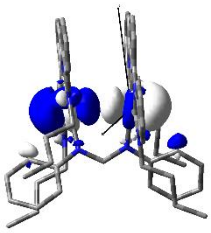

MO303 (H-1) -5.23 eV
Ni1 (45%) CCC1 (4%)
Ni2 (45%) CCC2 (4%)
NN (2%)

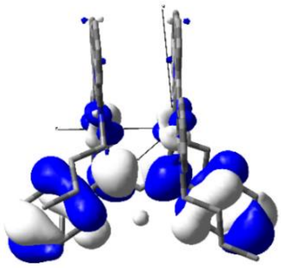

MO304 (HOMO) -5.16 eV
Ni1 (8%) CCC1 (4%)
Ni2 (8%) CCC2 (3%)
NN (77%)

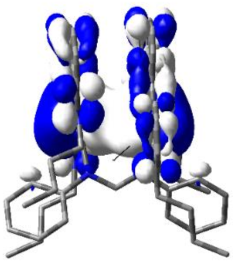

MO305 (LUMO) -2.53 eV
Ni1 (10%) CCC1 (38%)
Ni2 (10%) CCC2 (38%)
NN (2%)

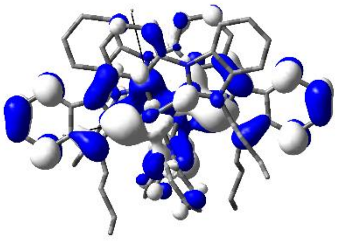

MO306 (L+1) -1.85 eV
Ni1 (6%) CCC1 (37%)
Ni2 (6%) CCC2 (37%)
NN (14%)

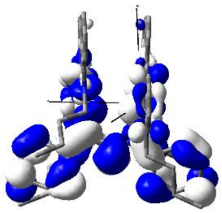

MO307 (L+2) -1.56 eV
Ni1 (7%) CCC1 (15%)
Ni2 (7%) CCC2 (14%)
NN (56%)

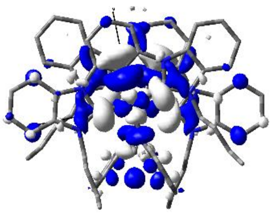

MO311 (L+6) -1.11 eV
Ni1 (22%) CCC1 (21%)
Ni2 (25%) CCC2 (22%)
NN (10%)

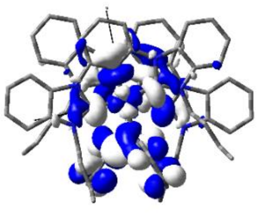

MO312 (L+7) -1.11 eV
Ni1 (22%) CCC1 (21%)
Ni2 (25%) CCC2 (22%)
NN (10%)

Figure S29. Frontier molecular orbital (MO) surfaces for **2** obtained from TPSSh-GD3BJ calculations at the M06L-optimzed ground state geometry (surface isovalue = 0.02 a.u.; CCC1 and CCC2 = carbene-phenyl-carbene pincer ligand coordinated to Ni1 and Ni2 respectively).

Table S14. Cartesian coordinates (Å) of **2** at the M06L-optimized ground state in THF solution.

	X	Y	Z
Ni1	-0.00789	0.037589	-0.00507
Ni2	-0.01622	0.037158	3.070209
C3	1.804252	0.092948	-0.78154
N4	2.261187	1.368219	-1.06356
C5	3.552141	1.351053	-1.56855
C6	4.456216	2.343105	-1.9472
H7	4.209514	3.398962	-1.90427
C8	5.715031	1.926881	-2.3681
H9	6.443686	2.676498	-2.66701
C10	6.070103	0.570573	-2.41008
H11	7.067356	0.289535	-2.73923
C12	5.170653	-0.42228	-2.03706
H13	5.444286	-1.47462	-2.06333
C14	3.908826	-0.00648	-1.62266
N15	2.819899	-0.73124	-1.14826
C16	2.809986	-2.18765	-1.0985
H17	1.982667	-2.47534	-0.44924
H18	3.740369	-2.51336	-0.61071
C19	2.646053	-2.82872	-2.46873
H20	3.542361	-2.65754	-3.08318
H21	1.813802	-2.33404	-2.99028
C22	2.356768	-4.31801	-2.35146
H23	3.177778	-4.81296	-1.81061
H24	1.456657	-4.45222	-1.72978
C25	2.148661	-4.98116	-3.70055
H26	1.912672	-6.04583	-3.59364
H27	3.043551	-4.89937	-4.33034
H28	1.318505	-4.51158	-4.24367
C29	0.122601	1.85861	-0.38204
C30	1.314545	2.398234	-0.84469
C31	1.465285	3.768411	-1.05058
H32	2.395158	4.208337	-1.39946
C33	0.371528	4.591179	-0.77053
H34	0.472439	5.664721	-0.91346
C35	-0.8443	4.082878	-0.30389

H36	-1.66673	4.760567	-0.09252
C37	-0.94571	2.705067	-0.11947
C38	-1.81702	0.597906	0.462393
N39	-2.05317	1.955848	0.338049
C40	-3.34478	2.285268	0.713878
C41	-4.05291	3.482401	0.797595
H42	-3.61263	4.436525	0.526188
C43	-5.36056	3.415311	1.267405
H44	-5.93772	4.333388	1.349174
C45	-5.95075	2.199129	1.642
H46	-6.97417	2.191647	2.009776
C47	-5.25207	1.000013	1.547311
H48	-5.70542	0.053195	1.835632
C49	-3.9448	1.068436	1.075916
N50	-2.98861	0.073159	0.899255
C51	-3.27572	-1.33138	1.157679
H52	-3.86319	-1.36962	2.086563
H53	-2.3211	-1.82914	1.351924
C54	-4.03989	-2.01599	0.03682
H55	-4.89875	-1.39548	-0.26093
H56	-3.39251	-2.10096	-0.8489
C57	-4.52336	-3.3915	0.474102
H58	-5.20666	-3.27671	1.331005
H59	-3.66544	-3.97117	0.848986
C60	-5.20804	-4.15678	-0.64297
H61	-5.55024	-5.14244	-0.30822
H62	-4.5238	-4.31022	-1.48745
H63	-6.08222	-3.61229	-1.02179
C64	1.379627	1.316302	2.599993
N65	1.014273	2.645114	2.72638
C66	2.04053	3.494412	2.34744
C67	2.1696	4.879277	2.262542
H68	1.365624	5.5542	2.538325
C69	3.378148	5.376303	1.785542
H70	3.507804	6.45284	1.70229
C71	4.428875	4.528117	1.405629
H72	5.355434	4.958044	1.032309

C73	4.309814	3.145557	1.502576
H74	5.122322	2.482763	1.209506
C75	3.101008	2.650014	1.981131
N76	2.661723	1.341946	2.159406
C77	3.519441	0.195086	1.893481
H78	4.062018	0.415106	0.962789
H79	2.867545	-0.66172	1.698887
C80	4.51109	-0.10096	3.006247
H81	5.018743	0.827822	3.307917
H82	3.970699	-0.46499	3.892866
C83	5.539977	-1.12752	2.553637
H84	6.094389	-0.72405	1.690778
H85	5.01624	-2.02257	2.183079
C86	6.503267	-1.5206	3.65814
H87	7.237247	-2.25707	3.312443
H88	5.964806	-1.95965	4.507979
H89	7.056567	-0.65008	4.032698
C90	-0.91159	1.628096	3.44688
C91	-0.30674	2.84957	3.184432
C92	-0.98653	4.052291	3.368498
H93	-0.53227	5.015972	3.155634
C94	-2.30219	3.993446	3.837503
H95	-2.8512	4.921272	3.981825
C96	-2.93967	2.78254	4.118748
H97	-3.9664	2.783488	4.473176
C98	-2.21975	1.607581	3.909841
C99	-1.6792	-0.68601	3.844515
N100	-2.6369	0.272007	4.126912
C101	-3.79855	-0.2953	4.627585
C102	-5.04103	0.214775	5.00323
H103	-5.26977	1.274626	4.959428
C104	-6.00222	-0.69999	5.420905
H105	-6.98228	-0.33374	5.716973
C106	-5.74367	-2.07801	5.462837
H107	-6.52597	-2.75863	5.789318
C108	-4.50534	-2.59086	5.092072
H109	-4.30295	-3.65927	5.1171

C110	-3.5417	-1.6753	4.680087
N111	-2.24712	-1.86542	4.207099
C112	-1.61538	-3.17779	4.158622
H113	-0.75164	-3.08756	3.499339
H114	-2.32234	-3.87334	3.682884
C115	-1.17731	-3.67673	5.527892
H116	-2.05318	-3.90039	6.154837
H117	-0.63118	-2.86909	6.0365
C118	-0.27893	-4.89933	5.41168
H119	-0.81591	-5.70506	4.888357
H120	0.582661	-4.6425	4.774166
C121	0.213292	-5.39202	6.760068
H122	0.882022	-6.25358	6.654457
H123	-0.6208	-5.69415	7.405903
H124	0.769165	-4.60446	7.284905
N125	-0.0968	-1.90335	0.474394
C126	-0.60467	-2.80736	-0.47155
C127	-1.04622	-4.10025	-0.13904
H128	-1.0448	-4.42317	0.901942
C129	-1.52643	-4.95663	-1.12582
H130	-1.87115	-5.95101	-0.84564
C131	-1.59628	-4.54275	-2.45505
H132	-1.978	-5.21472	-3.22079
C133	-1.19183	-3.24967	-2.78825
H134	-1.24963	-2.90725	-3.82022
C135	-0.70616	-2.39108	-1.80976
H136	-0.38073	-1.38041	-2.06573
C137	0.535437	-2.39768	1.529681
H138	0.779649	-3.47264	1.528366
N139	0.892583	-1.68043	2.585721
C140	1.745424	-2.27477	3.52896
C141	2.702566	-3.24921	3.194797
H142	2.835963	-3.546	2.154692
C143	3.513295	-3.80781	4.178887
H144	4.253964	-4.55477	3.896806
C145	3.404999	-3.39932	5.507132
H146	4.045733	-3.8354	6.270618

C147	2.480633	-2.40938	5.842062
H148	2.390517	-2.07133	6.873139
C149	1.664434	-1.8508	4.866281
H150	0.935106	-1.07991	5.124471

Table S15. Cartesian coordinates (Å) of **2** at the TDA-M06L-optimized S_1 excited state in THF solution.

	X	Y	Z
Ni1	-0.35275	-0.04811	0.214081
Ni2	0.320618	0.109649	2.844162
C3	1.426484	0.360996	-0.53528
N4	1.656995	1.709395	-0.76511
C5	2.930653	1.932316	-1.26577
C6	3.639927	3.075299	-1.62632
H7	3.215495	4.070733	-1.54764
C8	4.946354	2.897722	-2.07707
H9	5.526919	3.772841	-2.35999
C10	5.53304	1.629382	-2.15987
H11	6.557983	1.535054	-2.51044
C12	4.828152	0.482898	-1.79881
H13	5.279174	-0.50526	-1.86235
C14	3.518924	0.659405	-1.3595
N15	2.576596	-0.26302	-0.91625
C16	2.793183	-1.7017	-0.99805
H17	2.045629	-2.17367	-0.35868
H18	3.779533	-1.92377	-0.56319
C19	2.688994	-2.24019	-2.41758
H20	3.525549	-1.86954	-3.02824
H21	1.77232	-1.846	-2.87932
C22	2.652182	-3.7613	-2.43272
H23	3.561955	-4.15785	-1.95708
H24	1.809722	-4.09903	-1.80737
C25	2.508616	-4.32815	-3.83322
H26	2.461883	-5.4227	-3.82253
H27	3.352512	-4.03685	-4.47114
H28	1.591768	-3.96204	-4.31267
C29	-0.52995	1.797816	-0.06109
C30	0.551188	2.545241	-0.50908
C31	0.454117	3.92737	-0.66591
H32	1.287611	4.531942	-1.01174
C33	-0.77019	4.533572	-0.37337
H34	-0.86511	5.61014	-0.49524
C35	-1.87765	3.80532	0.071872

H36	-2.81003	4.31913	0.286656
C37	-1.73335	2.429942	0.229893
C38	-2.24996	0.17603	0.670193
N39	-2.69205	1.490526	0.655866
C40	-4.02937	1.58369	1.011878
C41	-4.91181	2.647411	1.19004
H42	-4.60915	3.680447	1.051281
C43	-6.21682	2.335105	1.569027
H44	-6.92914	3.144091	1.713575
C45	-6.62947	1.012847	1.7724
H46	-7.65474	0.812508	2.074874
C47	-5.74793	-0.05251	1.607327
H48	-6.05984	-1.0807	1.78112
C49	-4.44688	0.259573	1.227841
N50	-3.34292	-0.55983	1.019204
C51	-3.43112	-2.00635	1.145529
H52	-4.02198	-2.2075	2.052416
H53	-2.42007	-2.38442	1.323904
C54	-4.07926	-2.68412	-0.05254
H55	-4.96916	-2.11366	-0.35885
H56	-3.38657	-2.6639	-0.90735
C57	-4.47723	-4.11608	0.274941
H58	-5.2156	-4.10426	1.092419
H59	-3.60119	-4.65467	0.669086
C60	-5.03945	-4.85656	-0.92403
H61	-5.32173	-5.88448	-0.67038
H62	-4.30152	-4.90235	-1.73542
H63	-5.93163	-4.35412	-1.31872
C64	1.940955	1.119518	2.386996
N65	1.781093	2.497403	2.403645
C66	2.952611	3.152558	2.054165
C67	3.299042	4.491633	1.884138
H68	2.586169	5.297568	2.027082
C69	4.613669	4.766244	1.509147
H70	4.914728	5.802153	1.371394
C71	5.55003	3.746204	1.30227
H72	6.56368	4.002383	1.002794

C73	5.205512	2.406263	1.460676
H74	5.92544	1.609437	1.283416
C75	3.89471	2.133066	1.836888
N76	3.244639	0.920708	2.040431
C77	3.94105	-0.34907	1.901122
H78	4.547091	-0.27597	0.984846
H79	3.185912	-1.12232	1.732094
C80	4.837187	-0.68937	3.082454
H81	5.408745	0.203316	3.378449
H82	4.218285	-0.96504	3.949244
C83	5.796388	-1.81853	2.733666
H84	6.444667	-1.49387	1.90414
H85	5.222287	-2.67607	2.348749
C86	6.641935	-2.25562	3.91518
H87	7.325933	-3.06829	3.646118
H88	6.008005	-2.60987	4.73835
H89	7.246979	-1.42491	4.299901
C90	-0.30712	1.854287	3.116548
C91	0.513123	2.938776	2.828515
C92	0.05805	4.244465	2.988521
H93	0.682659	5.106182	2.772633
C94	-1.25347	4.43165	3.435806
H95	-1.62573	5.446073	3.559225
C96	-2.10259	3.361534	3.72899
H97	-3.11356	3.553057	4.077143
C98	-1.60224	2.070034	3.568571
C99	-1.46146	-0.2783	3.595396
N100	-2.24522	0.842486	3.826576
C101	-3.49055	0.5004	4.331229
C102	-4.61889	1.231028	4.695297
H103	-4.66062	2.31235	4.615163
C104	-5.72181	0.512597	5.152438
H105	-6.61954	1.055943	5.4384
C106	-5.70884	-0.88457	5.239342
H107	-6.59272	-1.40724	5.597013
C108	-4.58317	-1.62025	4.874365
H109	-4.56748	-2.70607	4.942993

C110	-3.47827	-0.90186	4.425927
N111	-2.23434	-1.33355	3.977104
C112	-1.81532	-2.72682	4.053326
H113	-0.95539	-2.83817	3.391372
H114	-2.62531	-3.34766	3.642147
C115	-1.45281	-3.16448	5.464863
H116	-2.35075	-3.18367	6.100105
H117	-0.77864	-2.41549	5.904793
C118	-0.77067	-4.52455	5.46567
H119	-1.43435	-5.27167	5.004325
H120	0.121494	-4.47073	4.820589
C121	-0.36871	-4.97594	6.857855
H122	0.144761	-5.94352	6.835624
H123	-1.24281	-5.07756	7.513249
H124	0.310379	-4.25106	7.324575
N125	-0.13046	-2.0104	0.510445
C126	-0.57822	-2.90533	-0.47749
C127	-0.94243	-4.23406	-0.20153
H128	-0.92162	-4.6026	0.824319
C129	-1.37733	-5.07036	-1.22519
H130	-1.66777	-6.09306	-0.98882
C131	-1.47461	-4.60276	-2.53506
H132	-1.82282	-5.26051	-3.32846
C133	-1.13988	-3.27775	-2.81235
H134	-1.21562	-2.89401	-3.82825
C135	-0.69996	-2.43802	-1.79577
H136	-0.42462	-1.40479	-2.01544
C137	0.554377	-2.49293	1.535855
H138	0.795593	-3.56555	1.540066
N139	0.963965	-1.75523	2.556279
C140	1.762849	-2.35512	3.54464
C141	2.646126	-3.41582	3.280504
H142	2.764838	-3.78499	2.26163
C143	3.409271	-3.9661	4.305561
H144	4.097091	-4.77938	4.07832
C145	3.325697	-3.46566	5.604216
H146	3.931104	-3.89657	6.398694

C147	2.47459	-2.39281	5.867903
H148	2.405025	-1.98267	6.873838
C149	1.705533	-1.841	4.850003
H150	1.03596	-1.00303	5.056052

Triplet excited states

Both UM06L and TDA-M06L methods predicted that the lowest energy triplet excited state of **1** is the ^{3}dd excited state.

Table S16. Selected bond distances (r/Å) and angles (°) of **1** at optimized S_0 and ^{3}dd excited state geometries

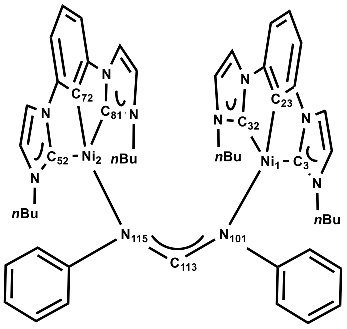

	S_0	^{3}dd		S_0	^{3}dd
r_{Ni1-C3}	1.96903 Å	1.97639 Å	$r_{Ni2-C52}$	1.96225 Å	2.08335 Å
$r_{Ni1-C23}$	1.86443 Å	1.87429 Å	$r_{Ni2-C72}$	1.86482 Å	1.95639 Å
$r_{Ni1-C32}$	1.96088 Å	1.97909 Å	$r_{Ni2-C81}$	1.96949 Å	2.10916 Å
$r_{Ni1-N101}$	1.99842 Å	1.98854 Å	$r_{Ni2-N115}$	1.99743 Å	2.07747 Å
$r_{Ni1-Ni2}$	2.90361 Å	2.76371 Å			
∠C3-Ni1-C32	159.526º	159.732º	∠C52-Ni2-C81	159.382º	151.017º
∠C23-Ni1-N101	177.903º	178.268º	∠C72-Ni2-N115	177.947º	169.567º
∠C3-Ni1-C23	79.988º	79.884º	∠C52-Ni2-C72	80.124º	78.609º
∠C23-Ni1-C32	80.136º	79.958º	∠C81-Ni2-C72	79.975º	78.304º
δC3-Ni1-C23-C32	174.658º	177.883º	δC52-Ni2-C72-C81	–174.581º	–162.329º
δC3-Ni1-N101-C113	77.771º	72.786º	δC52-Ni2-N115-C113	–106.824º	–119.734º

The emission spectra of the triplet excited states were simulated employing the time-dependent (TD) approach at 77K with the Franck-Condon assumption and harmonic oscillator approximation using the program, *FCclasses* (version 3.0.2); [25] the transition dipole moment of the triplet excited state emission is assumed to be unity. Since there is a large structural distortion between the ground state and the ^{3}dd excited state, the vertical hessian (VH) method is employed using internal coordinates.[26] The hwhm (half-width-at-half-maximum) for the spectrum simulation was determined from the solvent reorganization energy estimated using the corrected linear-response PCM approach[27-28]

$$hwhm = \sqrt{2(E^{neq} - E^{eq})k_B T ln2}$$

where E^{neq} and E^{eq} are respectively the non-equilibrium and equilibrium energies of the ground state at the optimized emitting triplet excited state geometry, k_B is the Boltzmann constant, and T is the temperature in K.

Table S17. Cartesian coordinates (Å) of **1** at the UM06L-optimized triplet excited state in THF solution.

	X	Y	Z
Ni1	-0.00105	0.073017	-0.14768
Ni2	0.21347	-0.17038	2.627253
C3	1.957285	0.152685	-0.40551
N4	3.056367	-0.63162	-0.55085
C5	4.193514	0.128753	-0.76636
H6	5.16176	-0.32947	-0.91175
C7	3.815938	1.43477	-0.74695
H8	4.389055	2.342813	-0.86337
N9	2.460178	1.429075	-0.52569
C10	3.057057	-2.09184	-0.54603
H11	2.440052	-2.41351	0.298379
H12	4.083617	-2.4144	-0.33459
C13	2.543529	-2.67689	-1.85009
H14	3.27918	-2.50562	-2.64994
H15	1.635668	-2.13125	-2.14341
C16	2.219945	-4.15776	-1.73081
H17	3.116345	-4.71541	-1.41914
H18	1.484091	-4.29342	-0.92063
C19	1.658459	-4.733	-3.01803
H20	1.394568	-5.79079	-2.90757
H21	2.382029	-4.65361	-3.8392
H22	0.750974	-4.19355	-3.32059
C23	0.239577	1.926488	-0.26732
C24	1.502844	2.465732	-0.43623
C25	1.71636	3.840382	-0.49615
H26	2.71211	4.263412	-0.61811
C27	0.598319	4.67522	-0.39637
H28	0.739268	5.752653	-0.44148
C29	-0.69644	4.166383	-0.24413
H30	-1.54702	4.841016	-0.16514
C31	-0.84449	2.784762	-0.18124
C32	-1.87652	0.674909	0.026887
N33	-2.03337	2.042771	-0.01648
C34	-3.34155	2.421939	0.163602
H35	-3.65844	3.454447	0.175346
C36	-4.04365	1.268282	0.312079

H37	-5.0976	1.094996	0.479829
N38	-3.14067	0.220614	0.225605
C39	-3.56656	-1.17034	0.348927
H40	-4.22144	-1.22137	1.22871
H41	-2.67449	-1.76495	0.568547
C42	-4.30622	-1.68825	-0.8727
H43	-3.61403	-1.76634	-1.72412
H44	-5.08086	-0.9624	-1.1644
C45	-4.95269	-3.03763	-0.59259
H46	-4.18096	-3.74222	-0.24566
H47	-5.66218	-2.93257	0.24317
C48	-5.66136	-3.60751	-1.80735
H49	-6.11071	-4.58402	-1.59477
H50	-4.96082	-3.73529	-2.64269
H51	-6.46231	-2.93996	-2.14963
C52	2.082284	0.81674	2.732917
N53	3.417814	0.652243	2.567897
C54	4.085576	1.864144	2.522602
H55	5.158233	1.9283	2.397442
C56	3.140687	2.835621	2.644187
H57	3.228639	3.912759	2.630746
N58	1.93163	2.184459	2.768662
C59	4.053789	-0.65801	2.605762
H60	4.89832	-0.65126	1.903548
H61	3.311069	-1.37451	2.231475
C62	4.499582	-1.05155	4.003315
H63	3.6386	-0.95707	4.682635
H64	5.262407	-0.34492	4.364464
C65	5.027922	-2.47674	4.042281
H66	4.248296	-3.15108	3.654874
H67	5.883032	-2.571	3.355273
C68	5.433642	-2.91537	5.437345
H69	6.241263	-2.28865	5.836817
H70	5.781708	-3.95468	5.448413
H71	4.586309	-2.84448	6.131503
C72	-0.33937	1.702527	2.900262
C73	0.615334	2.702057	2.969409
C74	0.290979	4.01929	3.28598

H75	1.037706	4.811741	3.328335
C76	-1.05015	4.303904	3.570482
H77	-1.33294	5.326228	3.812997
C78	-2.02826	3.302924	3.605987
H79	-3.04971	3.549126	3.895037
C80	-1.63103	2.003616	3.291003
C81	-1.81735	-0.39322	3.252622
N82	-2.43767	0.826669	3.406043
C83	-3.77034	0.685044	3.725296
H84	-4.43164	1.526003	3.879583
C85	-4.01205	-0.65406	3.775364
H86	-4.91761	-1.2054	3.99109
N87	-2.81531	-1.28255	3.474994
C88	-2.60579	-2.72632	3.453299
H89	-1.77589	-2.90788	2.759726
H90	-3.50315	-3.19572	3.027085
C91	-2.27561	-3.29657	4.822917
H92	-3.15198	-3.21547	5.483613
H93	-1.48512	-2.67926	5.275674
C94	-1.80224	-4.73989	4.737397
H95	-2.56691	-5.35175	4.234842
H96	-0.90754	-4.77956	4.094879
C97	-1.47932	-5.32937	6.098276
H98	-0.70997	-4.73627	6.609126
H99	-1.1027	-6.35512	6.014787
H100	-2.36502	-5.35029	6.74591
N101	-0.26784	-1.89778	-0.10367
C102	-0.89356	-2.50907	-1.20057
C103	-1.53899	-3.7575	-1.14538
H104	-1.61103	-4.29666	-0.20148
C105	-2.13865	-4.29251	-2.28093
H106	-2.64191	-5.25607	-2.2101
C107	-2.13356	-3.59637	-3.48914
H108	-2.61447	-4.01802	-4.3692
C109	-1.51786	-2.34605	-3.54907
H110	-1.50942	-1.78336	-4.48072
C111	-0.90659	-1.81113	-2.42142
H112	-0.41179	-0.83816	-2.46776

C113	0.132613	-2.64151	0.91766
H114	0.058499	-3.73493	0.825285
N115	0.630963	-2.13119	2.032782
C116	1.183445	-3.01771	2.968425
C117	1.208808	-2.62786	4.318839
H118	0.800538	-1.65301	4.593604
C119	1.714654	-3.46928	5.30179
H120	1.71051	-3.14156	6.340639
C121	2.215535	-4.7269	4.964917
H122	2.609665	-5.38704	5.734825
C123	2.23114	-5.11297	3.625715
H124	2.64972	-6.0771	3.341431
C125	1.740516	-4.2676	2.634343
H126	1.809602	-4.57359	1.590672

Table S18. Cartesian coordinates (Å) of **1** at the TDA-M06L-optimized triplet excited state in THF solution.

	X	Y	Z
Ni1	0.033314	0.083719	-0.12107
Ni2	0.200416	-0.17339	2.625575
C3	1.99282	0.180344	-0.36008
N4	3.097472	-0.5968	-0.4945
C5	4.232182	0.171753	-0.69438
H6	5.205149	-0.27966	-0.82944
C7	3.846072	1.475216	-0.6754
H8	4.414892	2.387121	-0.78199
N9	2.487498	1.460459	-0.4699
C10	3.10458	-2.05706	-0.50243
H11	2.465363	-2.38837	0.321481
H12	4.126262	-2.37773	-0.26646
C13	2.630249	-2.63107	-1.82598
H14	3.384866	-2.44368	-2.60417
H15	1.725459	-2.09006	-2.13679
C16	2.317208	-4.11603	-1.73338
H17	3.2111	-4.67005	-1.40853
H18	1.5638	-4.26892	-0.94279
C19	1.791233	-4.6781	-3.04125
H20	1.53531	-5.73985	-2.95202
H21	2.532653	-4.57998	-3.84428
H22	0.885589	-4.14314	-3.35702
C23	0.258504	1.94019	-0.24668
C24	1.520247	2.489063	-0.3947
C25	1.72203	3.865626	-0.45343
H26	2.715856	4.297633	-0.55839
C27	0.594784	4.690311	-0.37683
H28	0.726842	5.768843	-0.42181
C29	-0.69833	4.170282	-0.2493
H30	-1.5564	4.837379	-0.19011
C31	-0.83579	2.787709	-0.18558
C32	-1.85329	0.669179	0.000344
N33	-2.02116	2.035323	-0.04685
C34	-3.3378	2.401679	0.097868
H35	-3.66503	3.431019	0.101209

C36	-4.03237	1.241227	0.226097
H37	-5.08875	1.05788	0.364788
N38	-3.11744	0.201777	0.163223
C39	-3.53282	-1.19412	0.268552
H40	-4.20812	-1.25692	1.13202
H41	-2.64063	-1.78161	0.50634
C42	-4.23745	-1.7113	-0.974
H43	-3.52555	-1.77027	-1.81053
H44	-5.01681	-0.99467	-1.27591
C45	-4.86922	-3.07308	-0.72194
H46	-4.09437	-3.77044	-0.36757
H47	-5.59701	-2.98814	0.100238
C48	-5.54469	-3.63916	-1.95726
H49	-5.98381	-4.62445	-1.76477
H50	-4.82553	-3.74722	-2.77949
H51	-6.34832	-2.97941	-2.30829
C52	2.041833	0.790717	2.767155
N53	3.378179	0.603428	2.621151
C54	4.064762	1.805126	2.58402
H55	5.139998	1.851361	2.474295
C56	3.134958	2.792856	2.688113
H57	3.239921	3.868417	2.671848
N58	1.914004	2.161316	2.793596
C59	3.996854	-0.71376	2.682599
H60	4.854989	-0.72286	1.9968
H61	3.253007	-1.42485	2.300616
C62	4.41221	-1.09798	4.09234
H63	3.540927	-0.98249	4.755047
H64	5.179255	-0.39839	4.458301
C65	4.918245	-2.53011	4.158748
H66	4.134564	-3.19795	3.768522
H67	5.782288	-2.64686	3.486606
C68	5.295626	-2.95485	5.566023
H69	6.106608	-2.33485	5.969155
H70	5.627302	-3.9991	5.597318
H71	4.438916	-2.86086	6.245796
C72	-0.35493	1.687687	2.861072

C73	0.601678	2.68558	2.968835
C74	0.274322	3.998784	3.295816
H75	1.024678	4.786	3.362525
C76	-1.07084	4.285839	3.55774
H77	-1.35529	5.305721	3.807914
C78	-2.05032	3.285724	3.564225
H79	-3.07661	3.52787	3.838829
C80	-1.65202	1.98925	3.241595
C81	-1.81385	-0.3988	3.209069
N82	-2.45496	0.813321	3.338367
C83	-3.79396	0.657567	3.620347
H84	-4.46899	1.491479	3.751537
C85	-4.01984	-0.68422	3.668079
H86	-4.92447	-1.24622	3.858902
N87	-2.80803	-1.30051	3.404282
C88	-2.58696	-2.74231	3.414157
H89	-1.71111	-2.92623	2.781541
H90	-3.45025	-3.22398	2.934044
C91	-2.34743	-3.29306	4.810309
H92	-3.26457	-3.20235	5.411859
H93	-1.58675	-2.66986	5.304142
C94	-1.87187	-4.73764	4.774343
H95	-2.61221	-5.35823	4.246755
H96	-0.9478	-4.78885	4.176008
C97	-1.61454	-5.30257	6.159305
H98	-0.86693	-4.7025	6.693864
H99	-1.23865	-6.33093	6.11211
H100	-2.52905	-5.30864	6.765942
N101	-0.22088	-1.8885	-0.11639
C102	-0.81068	-2.4873	-1.23857
C103	-1.43833	-3.74618	-1.22597
H104	-1.52199	-4.3093	-0.29735
C105	-2.00563	-4.26332	-2.38609
H106	-2.49624	-5.23521	-2.3475
C107	-1.98353	-3.54104	-3.57876
H108	-2.43894	-3.94968	-4.47827
C109	-1.38333	-2.28189	-3.59767

H110	-1.3613	-1.69889	-4.51652
C111	-0.80602	-1.76358	-2.44484
H112	-0.32493	-0.78256	-2.45926
C113	0.149992	-2.63526	0.913915
H114	0.074183	-3.72812	0.820356
N115	0.618562	-2.12229	2.040073
C116	1.117805	-3.00551	3.0092
C117	1.110582	-2.58563	4.351084
H118	0.71951	-1.59338	4.589438
C119	1.56299	-3.41673	5.368392
H120	1.535105	-3.06516	6.399072
C121	2.04096	-4.69403	5.075544
H122	2.393167	-5.34628	5.87208
C123	2.086351	-5.11186	3.746458
H124	2.486646	-6.09334	3.497395
C125	1.648668	-4.27791	2.721132
H126	1.740181	-4.61215	1.688292

Photooxidation of cyclo-dienes

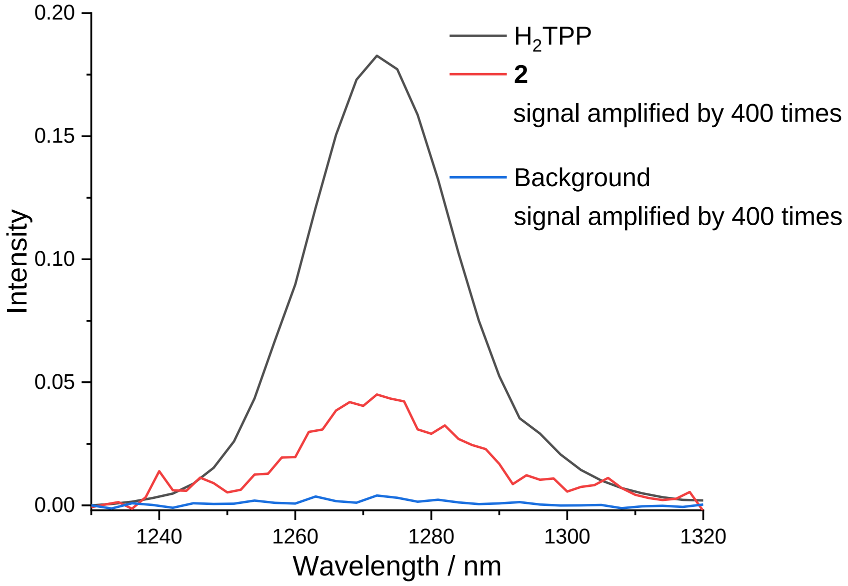

Figure S30. The emission spectra of $^1O_2$ generated by **2** in $CDCl_3$ compared with H_2TPP and background (only $CDCl_3$, without **2**). The $^1O_2$ signal intensity of **2** and background were amplified by 400 times

.

General procedure of photo-induced oxidation of cyclo-dienes

An NMR tube was charged with the cyclo-diene substrate (0.01 mmol), **2** (5 mol%), 1,3,5-trimethoxybenzene (0.01 mmol) and $CDCl_3$ (0.6 mL). Then the mixture solution was irradiated with a 550 nm LED (6 W) under oxygen atmosphere. The substrate conversion and product yield were determined by ^1H NMR. The ^1H NMR spectral data of **2a** and **2c** are in agreement with the published ones.[29] Control experiments show that no reaction occurred in the absence of light at room temperature or under reflux.

2b ^1H NMR (400 MHz, $CDCl_3$) δ 2.01 (q, J = 6.6 Hz, 1H), 1.68 (s, 6H), 1.37 (s, 6H), 0.74 (d, J = 6.5 Hz, 3H). ^{13}C NMR (101 MHz, $CDCl_3$) δ 136.17, 94.63, 61.96, 11.25, 10.69, 9.67. HRMS(EI) for $C_{10}H_{16}O_2$, calcd. 168.1145, found 168.1146.

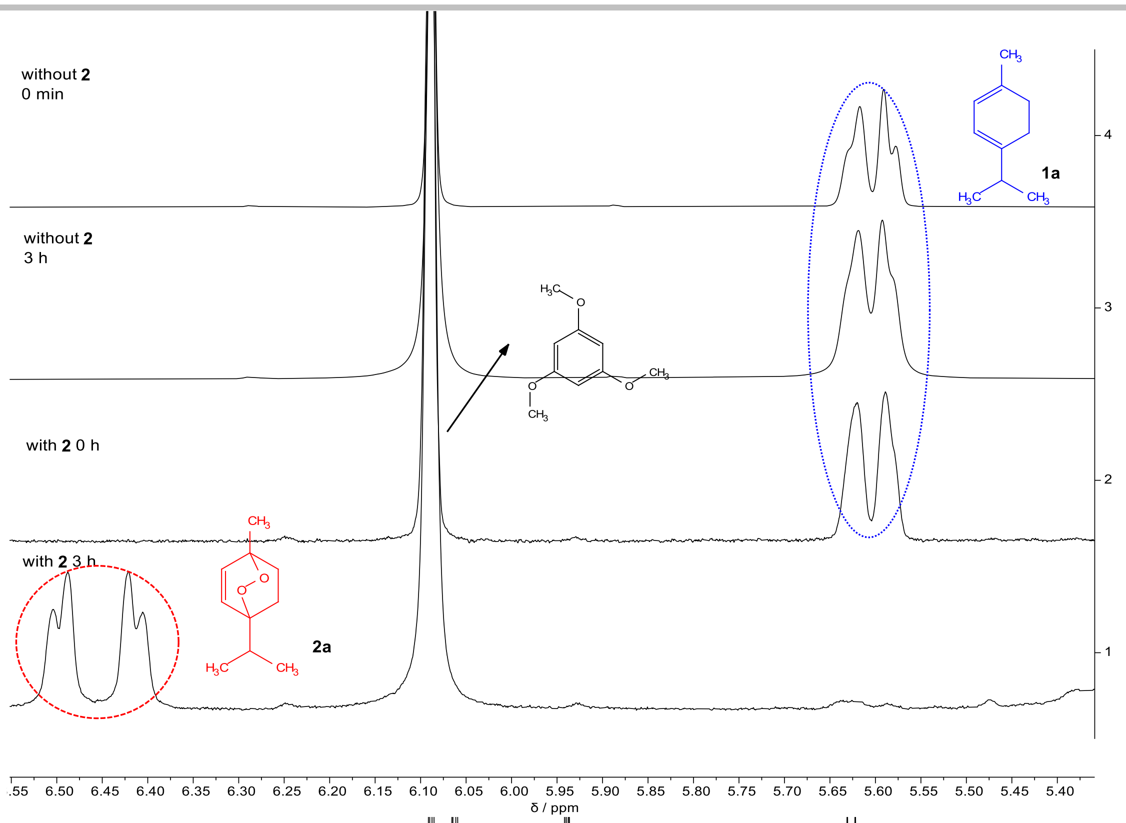
without 2
0 min
without 2
3 h
with 2 0 h
with 2 3 h
1a
2a
δ / ppm

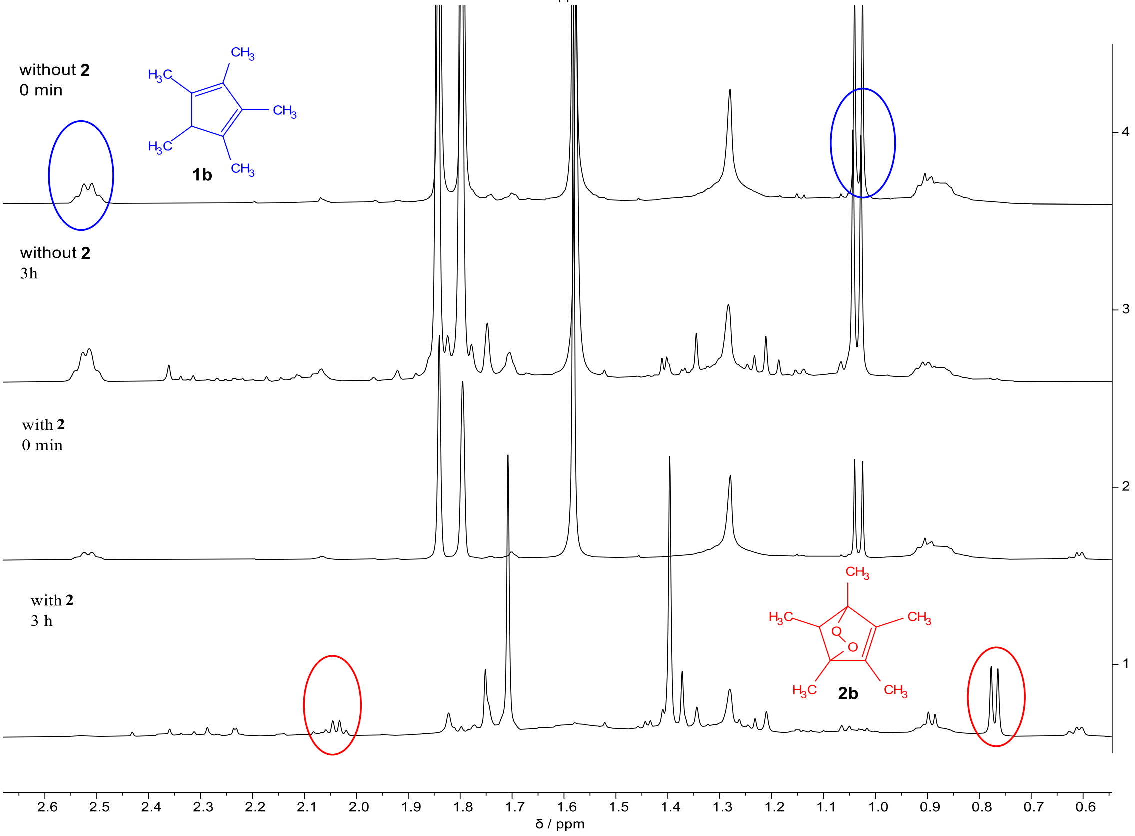
without 2
0 min
1b
without 2
3h
with 2
0 min
with 2
3 h
2b
δ / ppm

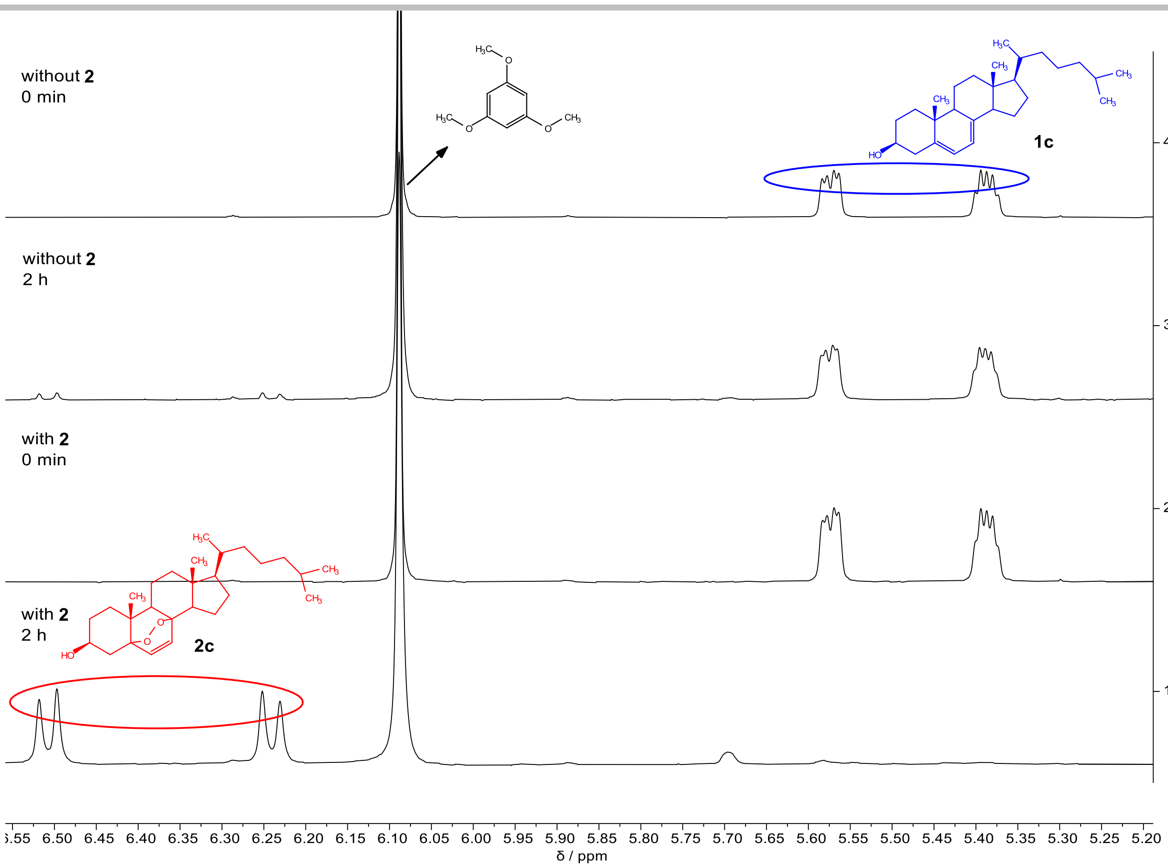

Figure S31. A part of ^{1}H NMR spectra of the photo-oxidation of cyclo-dienes in $CDCl_3$.

References

[1] Y. Zhao, G. W. Trucks, *J. Chem. Phys.* **2006**, *125*, 194101.
[2] M. J. Frisch, G. W. Trucks, H. B. Schlegel, G. E. Scuseria, M. A. Robb, J. R. Cheeseman, G. Scalmani, V. Barone, G. A. Petersson, H. Nakatsuji, X. Li, M. Caricato, A. V. Marenich, J. Bloino, B. G. Janesko, R. Gomperts, B. Mennucci, H. P. Hratchian, J. V. Ortiz, A. F. Izmaylov, J. L. Sonnenberg, D. Williams-Young, F. Ding, F. Lipparini, F. Egidi, J. Goings, B. Peng, A. Petrone, T. Henderson, D. Ranasinghe, V. G. Zakrzewski, J. Gao, N. Rega, G. Zheng, W. Liang, M. Hada, M. Ehara, K. Toyota, R. Fukuda, J. Hasegawa, M. Ishida, T. Nakajima, Y. Honda, O. Kitao, H. Nakai, T. Vreven, K. Throssell, J. A. Montgomery Jr, J. E. Peralta, F. Ogliaro, M. Bearpark, J. J. Heyd, E. Brothers, K. N. Kudin, V. N. Staroverov, T. A. Keith, R. Kobayashi, J. Normand, K. Raghavachari, A. Rendell, J. C. Burant, S. S. Iyengar, J. Tomasi, M. Cossi, J. M. Millam, M. Klene, C. Adamo, R. Cammi, J. W. Ochterski, R. L. Martin, K. Morokuma, O. Farkas, J. B. Foresman, D. J. Fox, Revision C.01 ed., Gaussian, Inc., Wallingford CT, **2016**.
[3] M. M. Francl, W. J. Petro, W. J. Hehre, J. S. Binkley, M. S. Gordon, D. J. DeFree, J. A. Pople, *J. Chem. Phys.* **1982**, *77*, 3654.
[4] P. C. Hariharan, J. A. Pople, *Theor. Chim. Acta* **1973**, *28*, 213.
[5] F. Weigend, R. Ahlrichs, *Phys. Chem. Chem. Phys.* **2005**, *7*, 3297-3305.
[6] D. Feller, *J. Comp. Chem.* **1996**, *17*, 1571-1588.
[7] B. P. Pritchard, D. Altarawy, B. Didier, T. D. Gibson, T. L. Windus, *J. Chem. Inf. Model.* **2019**, *59*, 4814-4820.
[8] K. I. Schuchardt, B. T. Didier, T. Elsethagen, L. Sun, V. Gurumoorthi, H. J. Chase, J. Li, T. L. Windus, *J. Chem. Inf. Model.* **2007**, *47*, 1045-1052.
[9] M. Cossi, G. Scalmani, N. Rega, V. Barone, *J. Chem. Phys.* **2002**, *117*, 43-54.
[10] S. Hirata, M. Head-Gordon, *Chem. Phys. Lett.* **1999**, *314*, 291-299.
[11] M. E. Casida, F. Gutierrez, J. Guan, F.-X. Gadea, d. Salahub, J.-P. Daudey, *J. Chem. Phys.* **2000**, *113*, 7062-7071.
[12] M. J. G. Peach, M. J. Williamson, D. J. Tozer, *J. Chem. Theory Comput.* **2011**, *7*, 3578-3585.
[13] T. Lu, F. Chen, *J. Comput. Chem.* **2012**, *33*, 580-592.
[14] R. Dennington, T. A. Keith, J. M. Millam, 6.016 ed., Semichem Inc., Shawnee Mission, KS, **2016**.
[15] N. M. O'Boyle, A. L. Tenderholt, K. M. Langner, *J. Comp. Chem.* **2008**, *29*, 839-845.
[16] a) B. A. Dar, S. N. Ahmad, M. A. Wagay, A. Hussain, N. Ahmad, K. A. Bhat, M. A. Khuroo, M. Sharma, B. Singh, *Tetrahedron Lett.* **2013,** *54*, 4880-4884; b) A. J. Huckaba, T. K. Hollis, T. O. Howell, H. U. Valle, Y. Wu, *Organometallics* **2013,** *32*, 63-69. c) J. D. Cope, J. A. Denny, R. W. Lamb, L. E. McNamara, N. I. Hammer, C. E. Webster, T. K. Hollis, *J. Organomet. Chem.* **2017,** *845*, 258-265; d) J. Liu, T.-L. Lam, M.-K. Sit, Q. Wan, C. Yang, G. Cheng, C.-M. Che, *J. Mater. Chem. C* **2022,** *10*, 10271-10283.
[17] Y. Zhao, D. G. Truhlar, *Theor. Chim. Acta* **2008**, *120*, 215-241.
[18] H. S. Yu, X. He, S. L. Li, D. G. Truhlar, *Chem. Sci.* **2016**, *7*, 5032-5051.
[19] C. Adamo, V. Barone, *J. Chem. Phys.* **1999**, *110*, 6158-6169.
[20] S. Grimme, S. Ehrlich, L. Goerigk, *J. Comput. Chem.* **2011**, *32*, 1456-1465.
[21] V. N. Staroverov, G. E. Scuseria, J. Tao, J. P. Perdew, *J. Chem. Phys.* **2004**, *121*, 11507.
[22] J. Tao, J. P. Perdew, V. N. Staroverov, G. E. Scuseria, *Phys. Rev. Lett.* **2003**, *91*, 146401.
[23] V. Barone, M. Cossi, *J. Phys. Chem. A* **1998**, *102*, 1995-2001.
[24] M. Cossi, N. Rega, G. Scalmani, V. Barone, *J. Comp. Chem.* **2003**, *24*, 669-681.
[25] J. Cerezo, F. Santoro, *J. Comput. Chem.* **2023**, *44*, 626-643.
[26] J. Cerezo, F. Santoro, *J. Chem. Theory Comput.* **2016**, *12*, 4970-4985.
[27] A. Baiardi, J. Bloino, V. Barone, *J. Chem. Theory Comput.* **2013**, *9*, 4097-4115.
[28] J. Cerezo, F. J. A. Ferrer, G. Prampolini, F. Santoro, *J. Chem. Theory Comput.* **2015**, *11*, 5810-5823.
[29] a) P. Ghorai, P. H. Dussault, *Org. Lett.* **2009,** *11*, 4572-4575; b) K. Feng, L.-Z. Wu, L.-P. Zhang, C.-H. Tung, *Tetrahedron* **2007,** *63*, 4907-4911.

NMR spectra

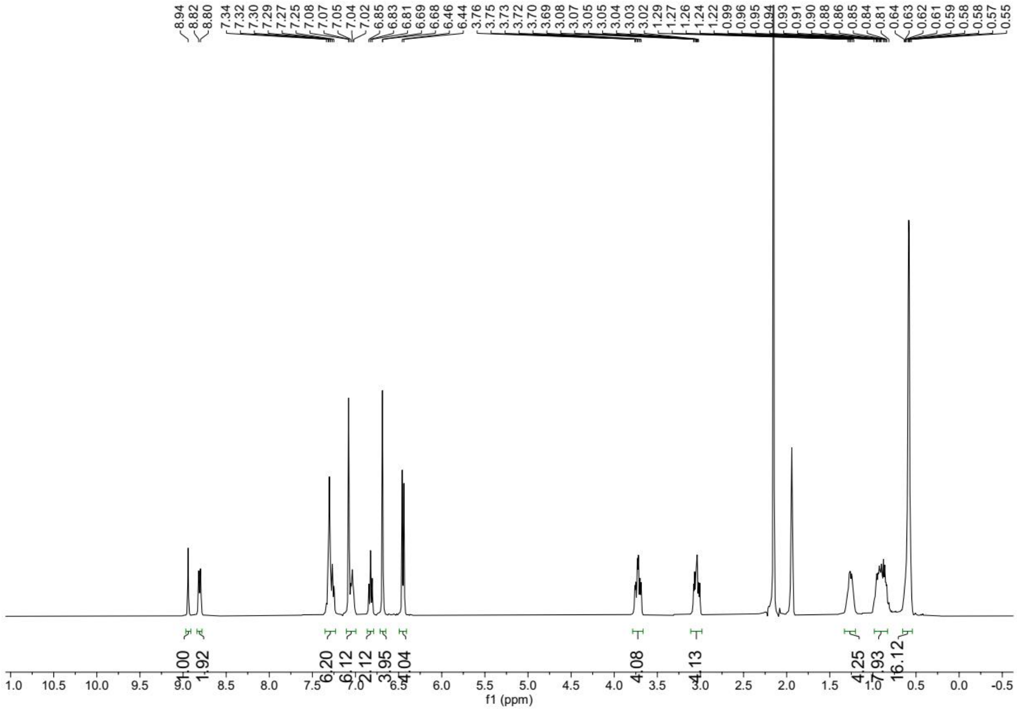

^{1}H NMR spectrum of **1** (CD_3CN).

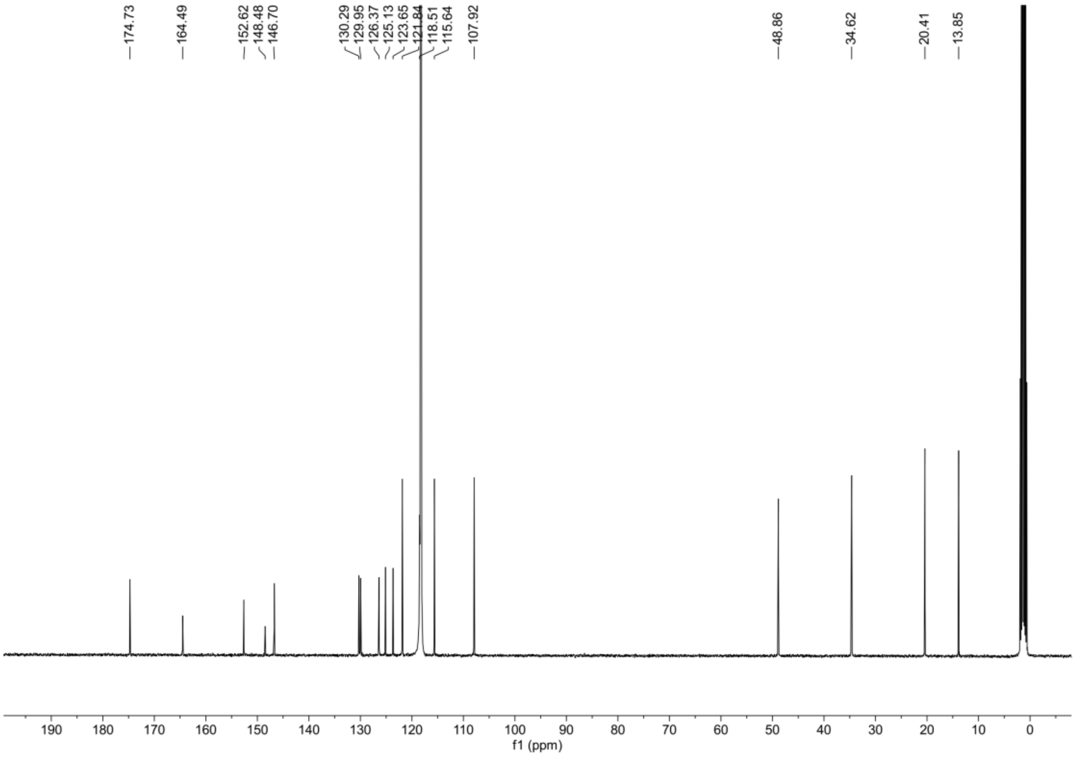

^{13}C NMR spectrum of **1** (CD_3CN).

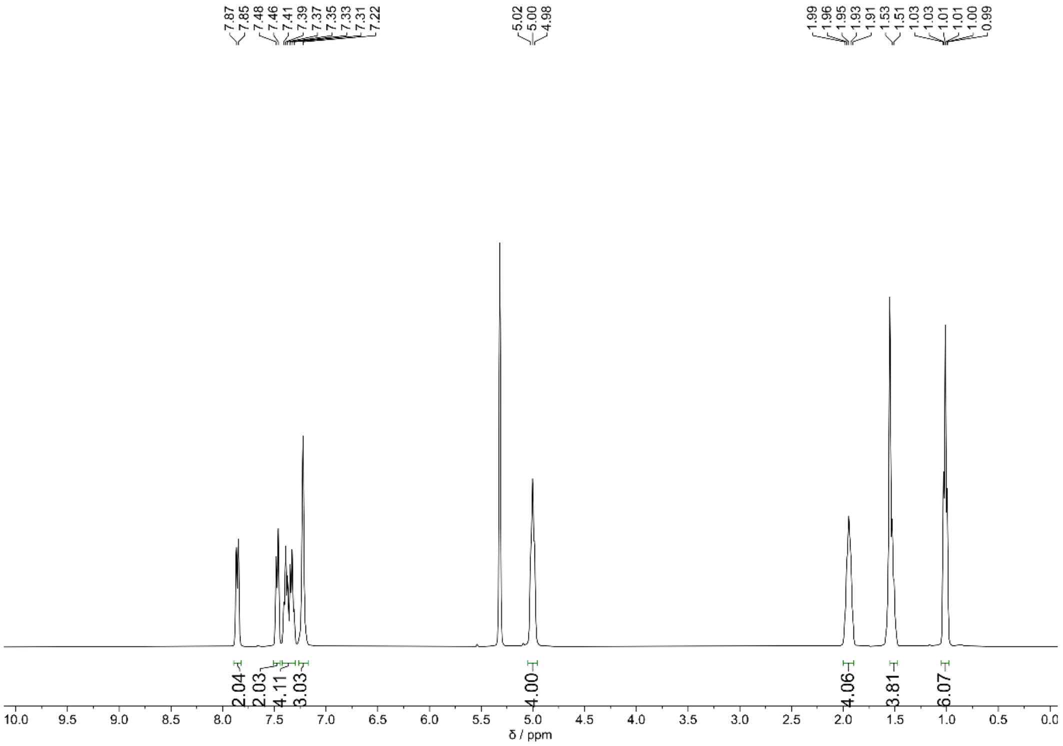

^{1}H NMR spectrum of ^{BIm}CCC-Ni-Cl (CD_2Cl_2).

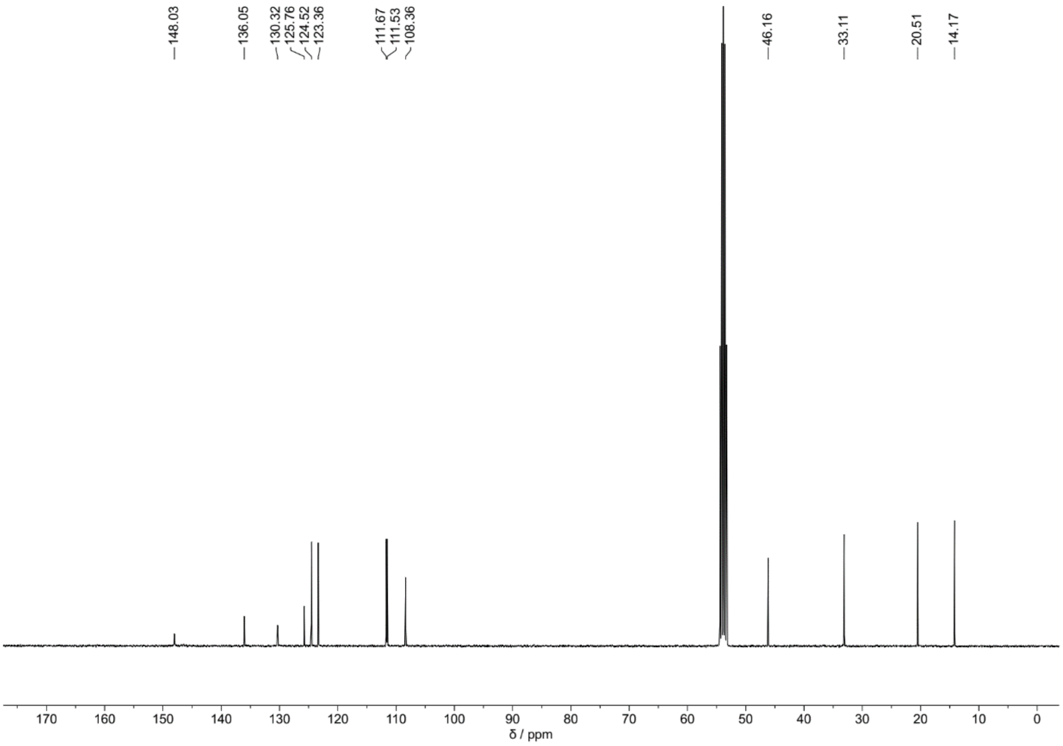

^{13}C NMR spectrum of ^{BIm}CCC-Ni-Cl (CD_2Cl_2).

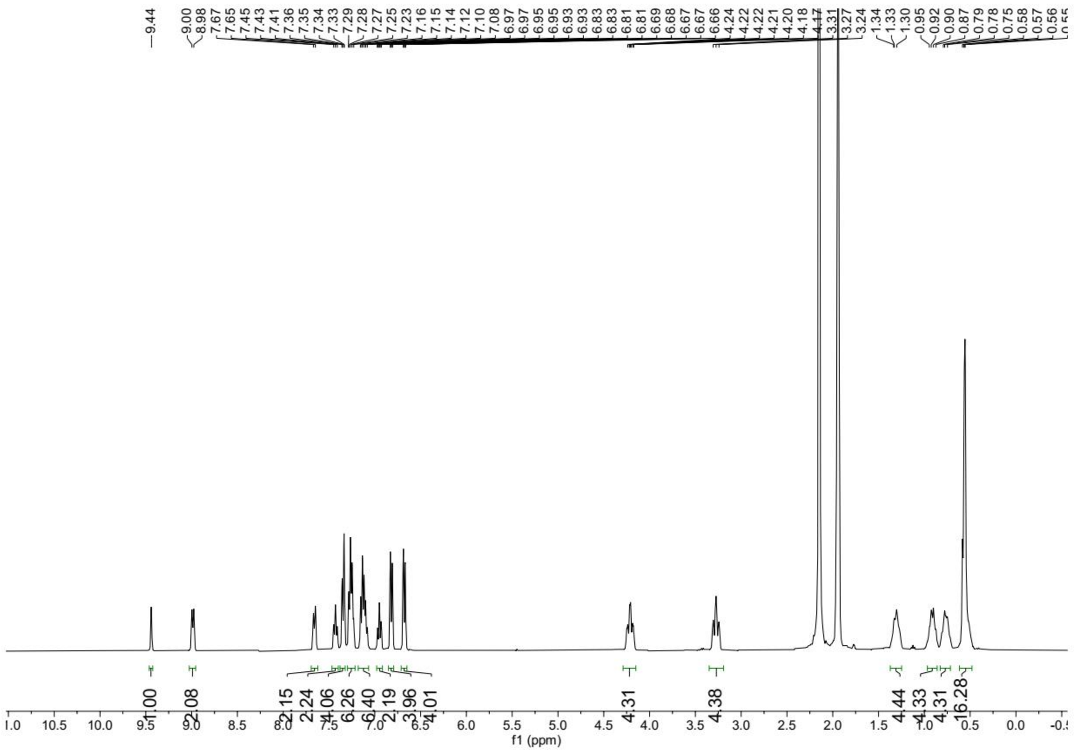

^{1}H NMR spectrum of **2** (CD_3CN).

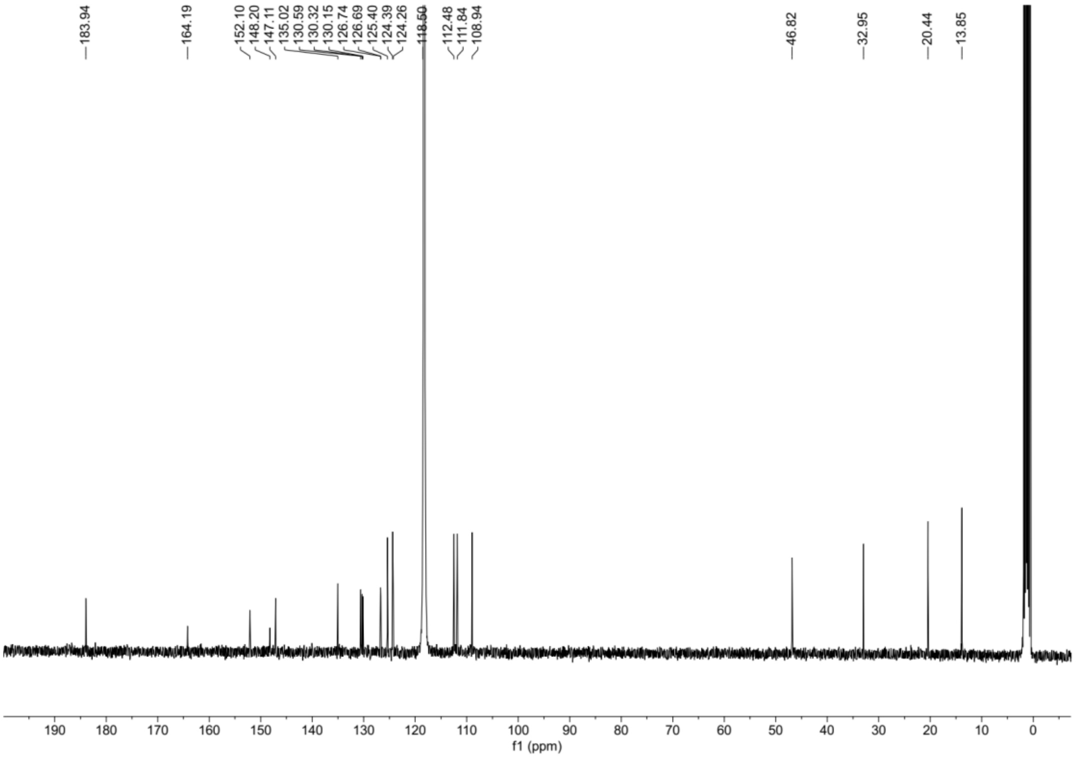

^{13}C NMR spectrum of **2** (CD_3CN).

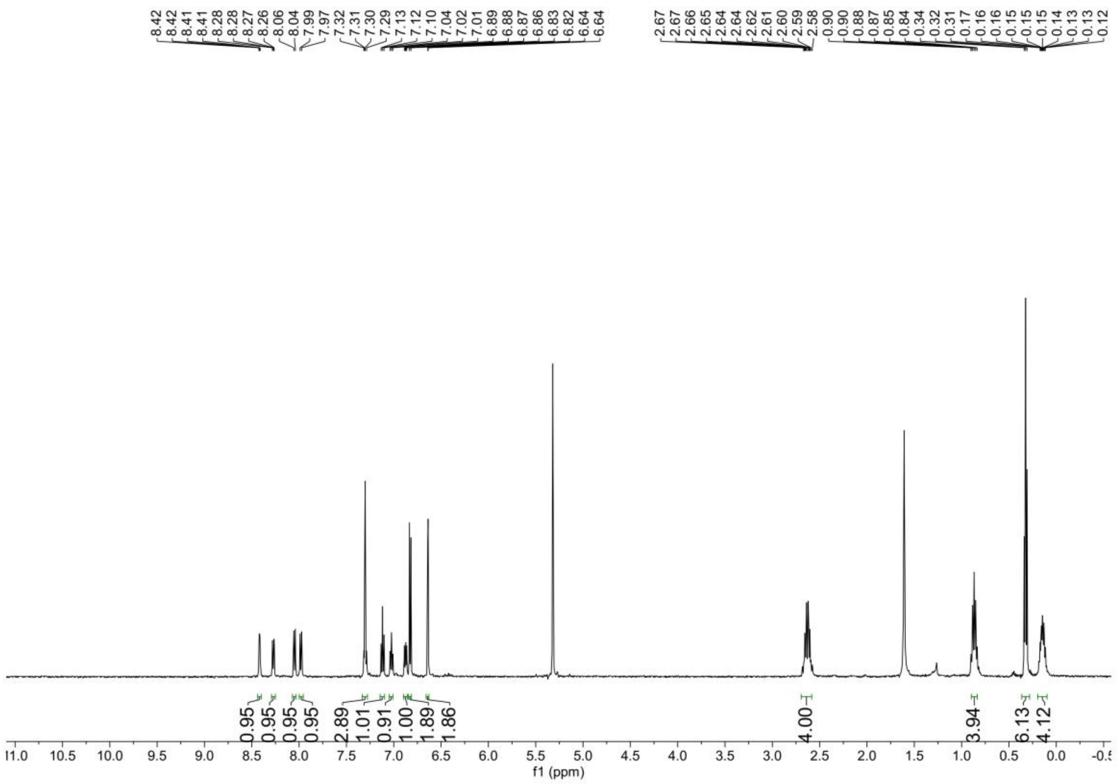

^{1}H NMR spectrum of **ImCCC-Ni-Cb** (CD_2Cl_2).

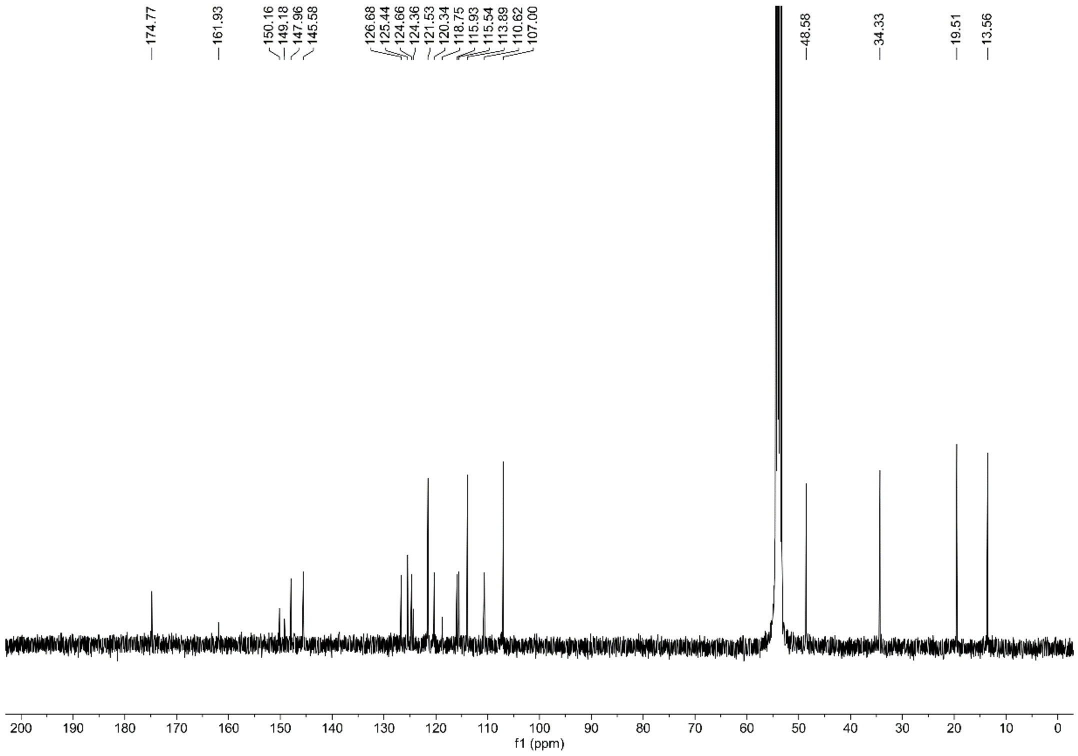

^{13}C NMR spectrum of **ImCCC-Ni-Cb** (CD_2Cl_2).

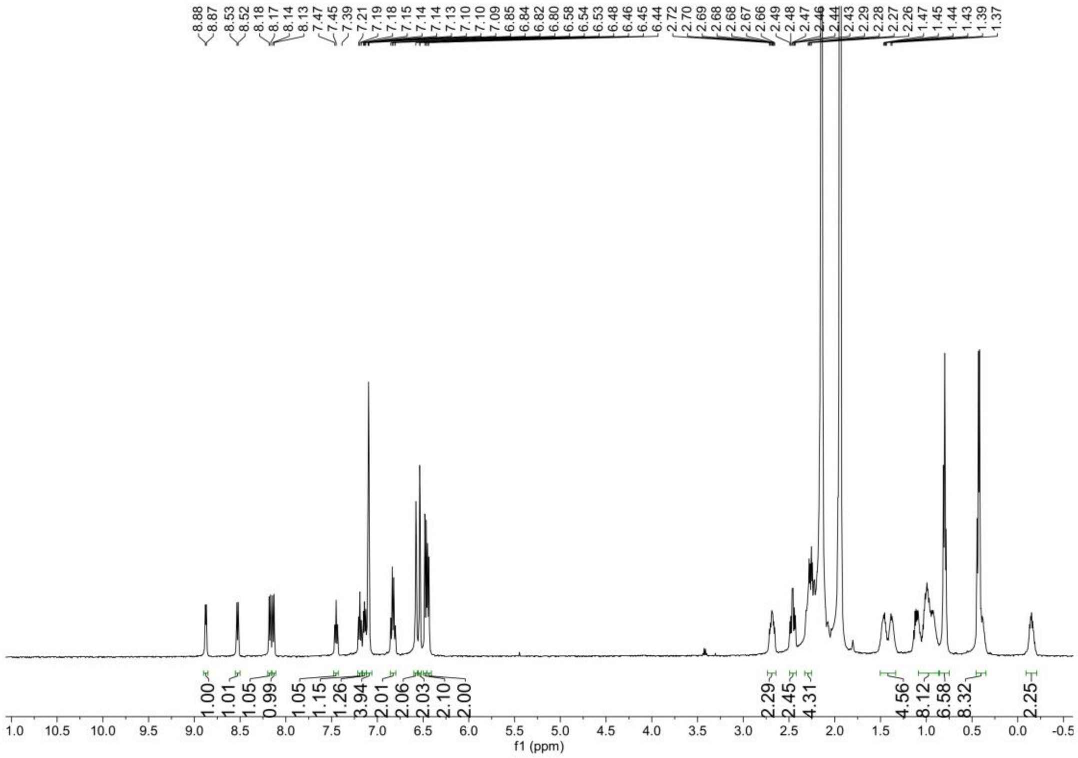

^{1}H NMR spectrum of **3** (CD_3CN).

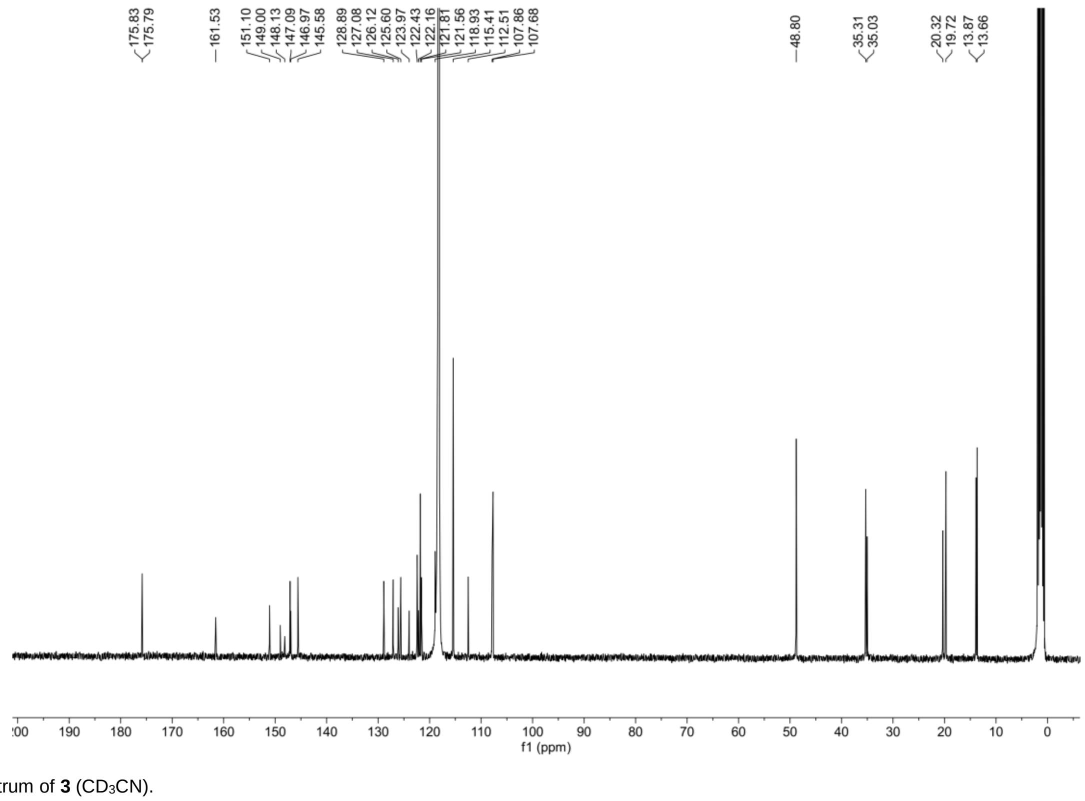

^{13}C NMR spectrum of **3** (CD_3CN).

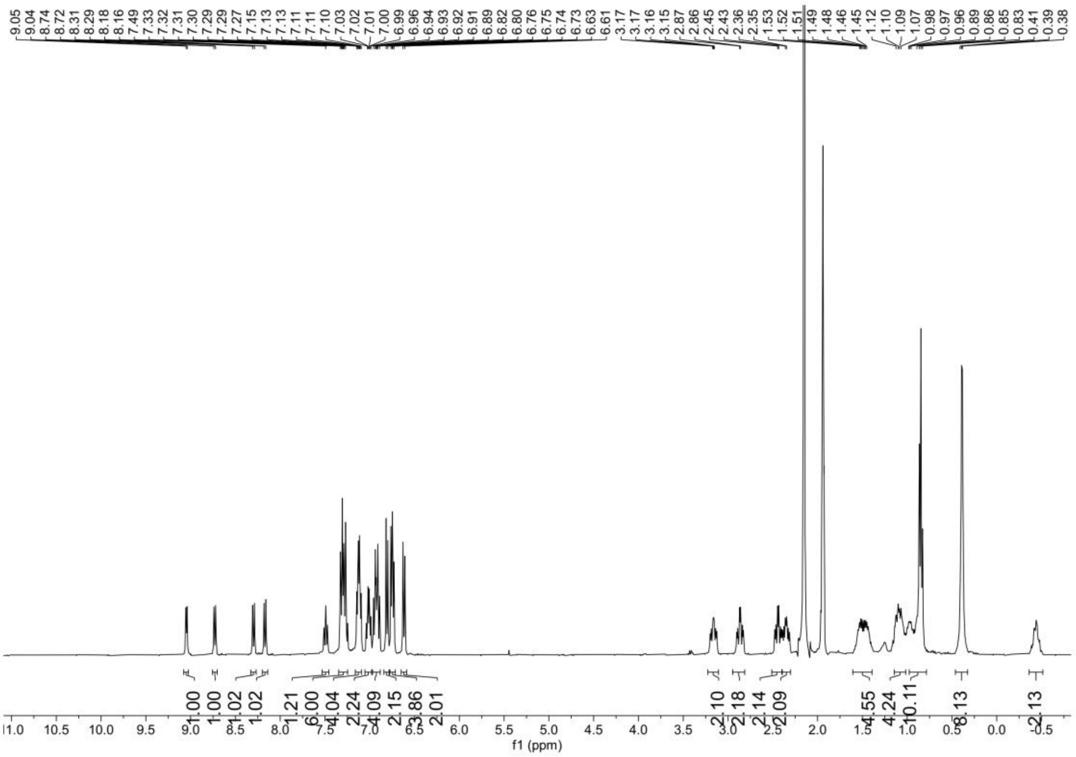

^{1}H NMR spectrum of **4** (CD_3CN).

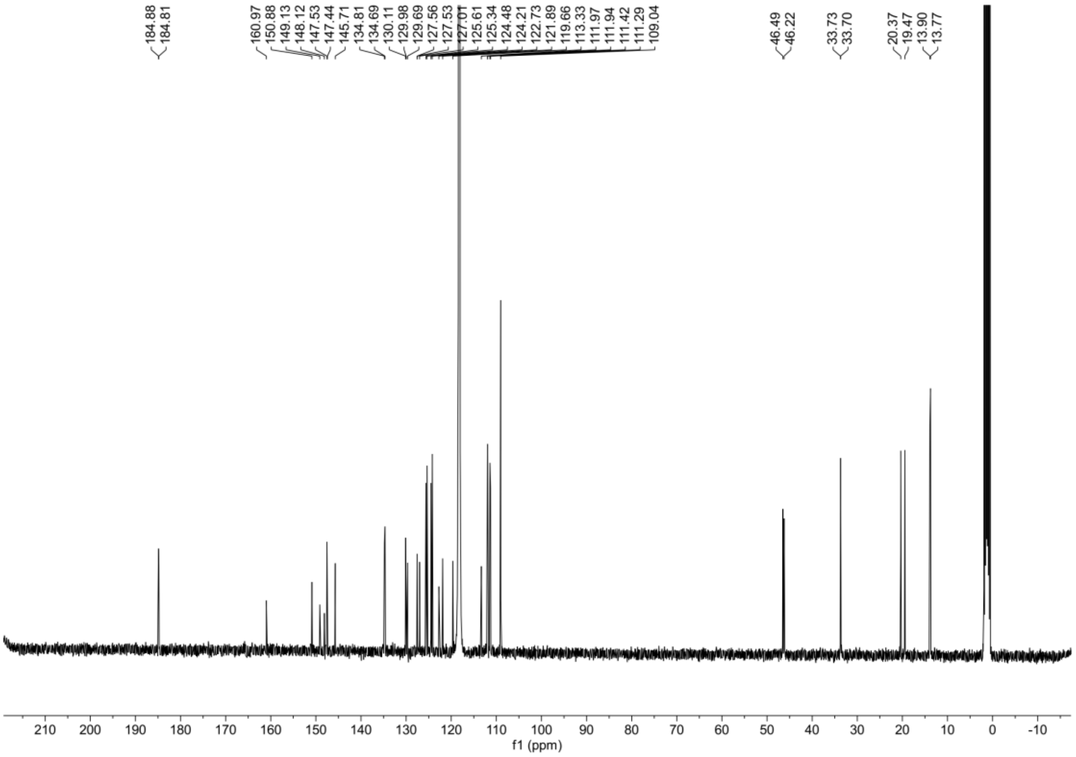

^{13}C NMR spectrum of **4** (CD_3CN).

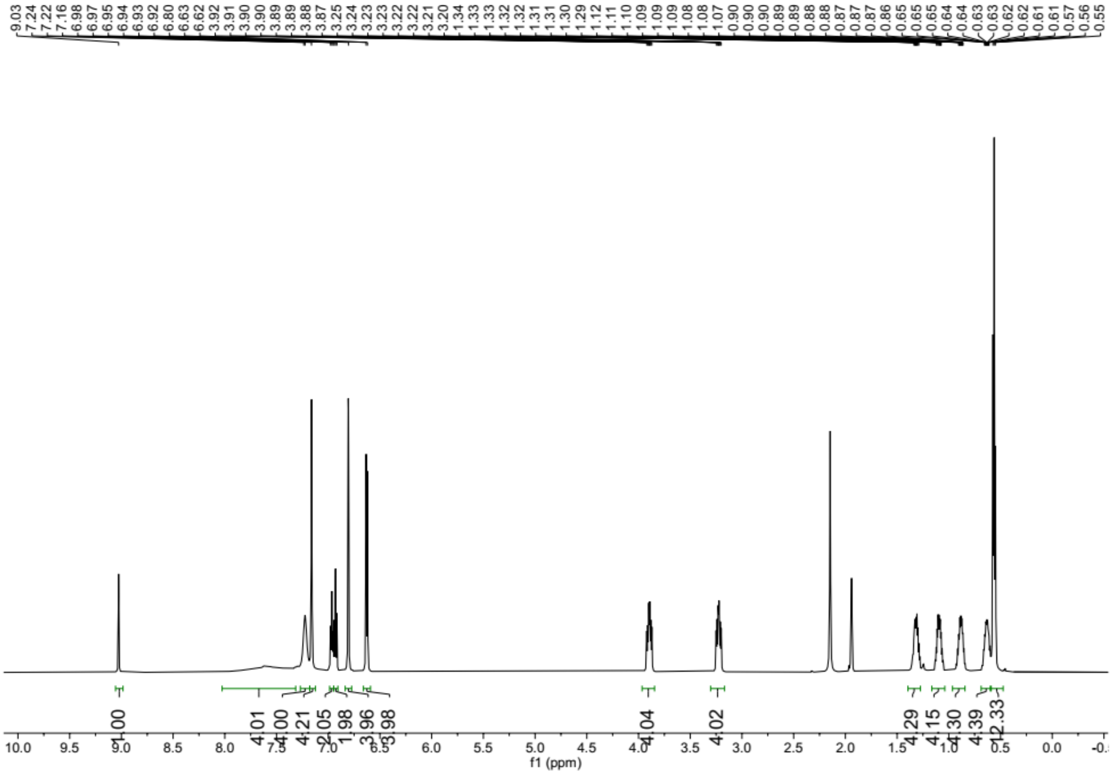

^{1}H NMR spectrum of **1-Pd** (CD_3CN).

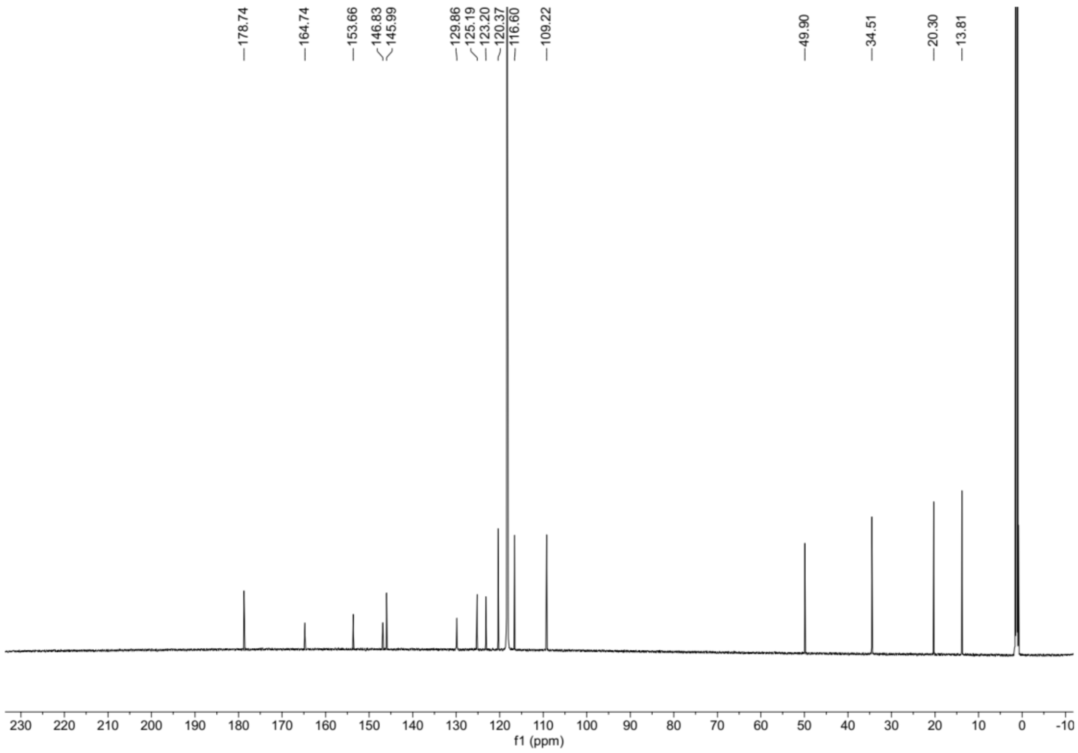

^{13}C NMR spectrum of **1-Pd** (CD_3CN).

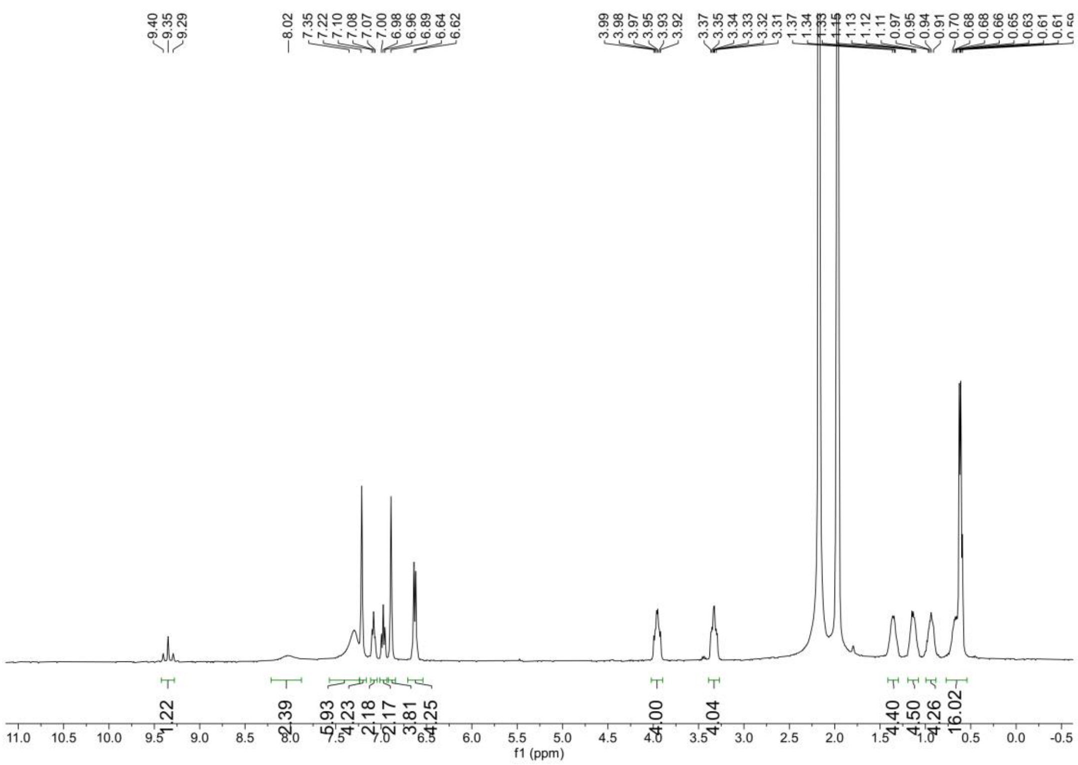

^{1}H NMR spectrum of **1-Pt** (CD_3CN).

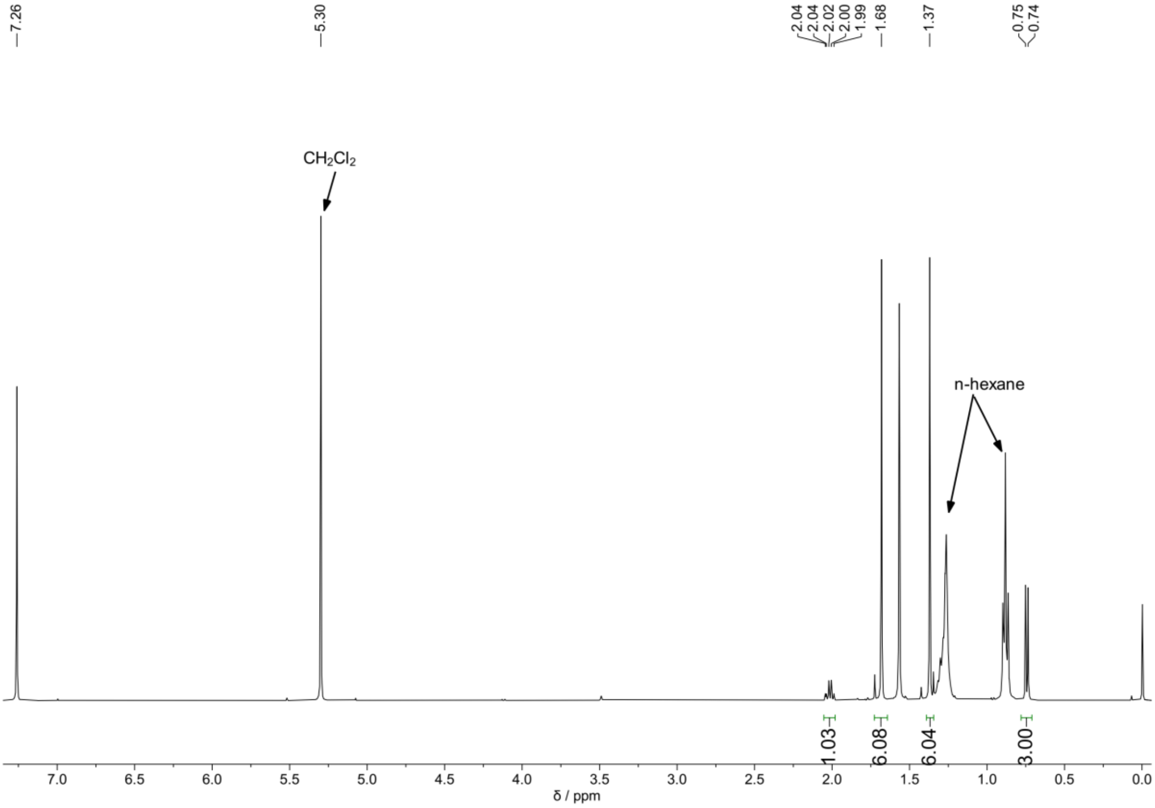

^{1}H NMR spectrum of **2b** ($CDCl_3$).

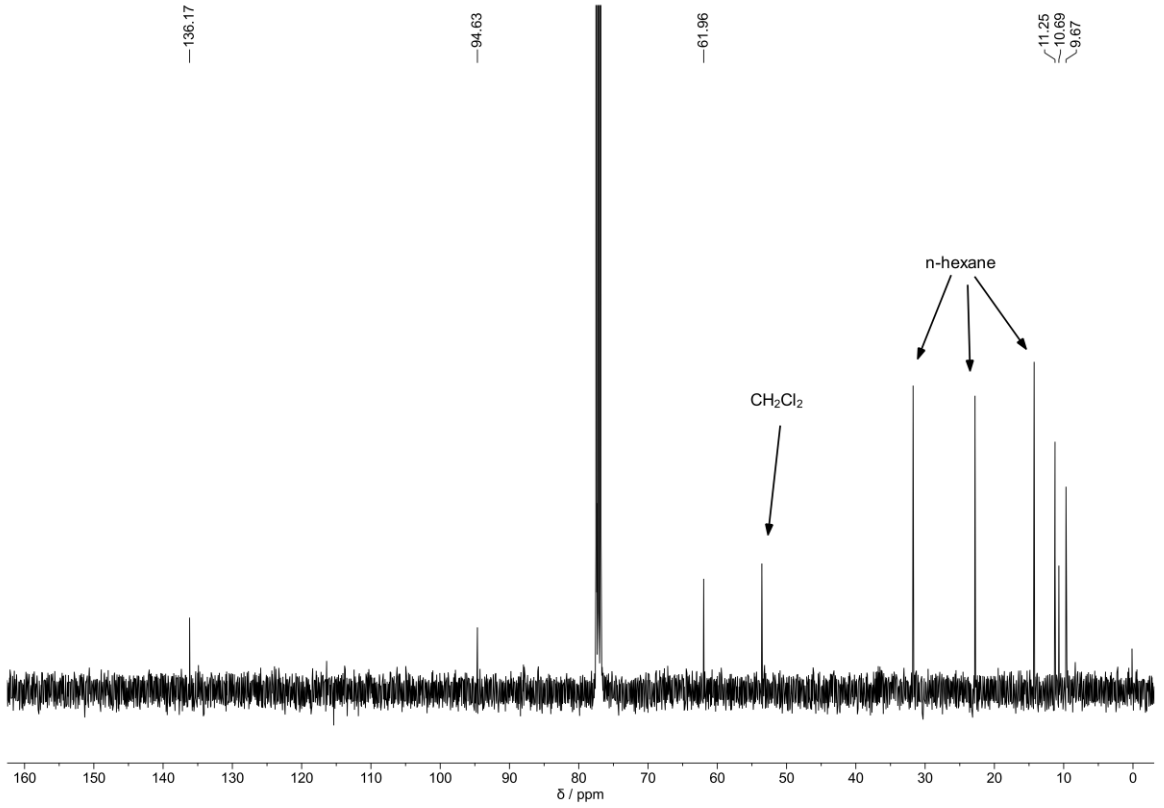

^{13}C NMR spectrum of **2b** ($CDCl_3$).
